# SMYD3 Impedes Small Cell Lung Cancer Sensitivity to Alkylation Damage through RNF113A Methylation–Phosphorylation Cross-talk

**DOI:** 10.1158/2159-8290.CD-21-0205

**Published:** 2022-07-12

**Authors:** Valentina Lukinović, Simone Hausmann, Gael S. Roth, Clement Oyeniran, Tanveer Ahmad, Ning Tsao, Joshua R. Brickner, Alexandre G. Casanova, Florent Chuffart, Ana Morales Benitez, Jessica Vayr, Rebecca Rodell, Marianne Tardif, Pascal W.T.C. Jansen, Yohann Couté, Michiel Vermeulen, Pierre Hainaut, Pawel K. Mazur, Nima Mosammaparast, Nicolas Reynoird

**Affiliations:** 1 Institute for Advanced Biosciences, Grenoble Alpes University, CNRS UMR5309, INSERM U1209, Grenoble, France.; 2 Department of Experimental Radiation Oncology, The University of Texas MD Anderson Cancer Center, Houston, Texas.; 3 Clinique universitaire d'Hépato-gastroentérologie et Oncologie digestive, CHU Grenoble Alpes, Grenoble, France.; 4 Department of Pathology and Immunology and Department of Medicine, Center for Genome Integrity, Washington University in St. Louis School of Medicine, St. Louis, Missouri.; 5 Univ. Grenoble Alpes, CEA, INSERM, IRIG, BGE, Grenoble, France.; 6 Department of Molecular Biology, Faculty of Science, Radboud Institute for Molecular Life Sciences, Oncode Institute, Radboud University Nijmegen, Nijmegen, the Netherlands.

## Abstract

Small cell lung cancer (SCLC) is the most fatal form of lung cancer, with dismal survival, limited therapeutic options, and rapid development of chemoresistance. We identified the lysine methyltransferase SMYD3 as a major regulator of SCLC sensitivity to alkylation-based chemotherapy. RNF113A methylation by SMYD3 impairs its interaction with the phosphatase PP4, controlling its phosphorylation levels. This cross-talk between posttranslational modifications acts as a key switch in promoting and maintaining RNF113A E3 ligase activity, essential for its role in alkylation damage response. In turn, SMYD3 inhibition restores SCLC vulnerability to alkylating chemotherapy. Our study sheds light on a novel role of SMYD3 in cancer, uncovering this enzyme as a mediator of alkylation damage sensitivity and providing a rationale for small-molecule SMYD3 inhibition to improve responses to established chemotherapy.

**Significance:**

SCLC rapidly becomes resistant to conventional chemotherapy, leaving patients with no alternative treatment options. Our data demonstrate that SMYD3 upregulation and RNF113A methylation in SCLC are key mechanisms that control the alkylation damage response. Notably, SMYD3 inhibition sensitizes cells to alkylating agents and promotes sustained SCLC response to chemotherapy.

*This article is highlighted in the In This Issue feature, p. 2007*

## Teaser

RNF113A methylation by SMYD3 improves alkylation damage response and impairs small cell lung cancer sensitivity to alkylation-based chemotherapy.

## INTRODUCTION

Small cell lung cancer (SCLC) accounts for approximately 15% of lung cancer and is a highly malignant and nearly uniformly fatal disease (1). To date, no targeted therapy has been approved for SCLC, which remains commonly treated with conventional chemotherapy. In the last decades, first-line platinum-based chemotherapy (cisplatin or carboplatin with etoposide) replaced previously used alkylating-based chemotherapy (cyclophosphamide + doxorubicin + vincristine) in SCLC due to lower toxicity, but without better efficacy. Interestingly, a combination of both platinum- and alkylating-based chemotherapies might improve SCLC progression-free survival (2–5). Notably, alkylation-based chemotherapy remains frequently used after initial treatments have failed, and combination therapies using alkylators remain under investigation in SCLC (2, 6). Furthermore, although both regimens inevitably lead to acquired resistance, studies demonstrate that alkylating chemotherapy is still modestly efficacious in SCLC resistant to platinum-based agents, whereas the opposite is not true (2). Regardless, systemic treatment for patients with SCLC has not changed significantly in the past decades, and the efficacy of both cisplatin- and alkylating-based regimens remains insufficient, with a 5-year survival rate below 7% (7). Indeed, SCLC is initially sensitive to first-line therapy, but most patients rapidly relapse with chemotherapy-resistant disease and rarely survive beyond one year because of the absence of alternative treatment options (8, 9). Therefore, having a better understanding of the molecular mechanisms that drive therapeutic resistance is of great clinical interest and necessary to develop and improve novel therapies effective for SCLC.

Here, we seek to identify mechanisms that promote SCLC tolerance to platinum-containing drugs such as cisplatin and alkylating agents such as cyclophosphamide (CP). We perform pharmacologic screening of 285 clinically approved and experimental small-molecule inhibitors to facilitate the potential implementation of promising combination therapeutic strategies. Interestingly, our synthetic lethality screening reveals that the small-molecule inhibition of the SMYD3 (SET and MYND domain containing protein 3; KMT3E) lysine methyltransferase potentiates alkylating agent efficacy in SCLC. We further validate the capacity of SMYD3 to sensitize SCLC cells to alkylating damage in subsequent genetic and pharmacologic studies, both *in vitro* and *in vivo*.

Lysine methylation signaling contributes to numerous aspects of cell physiology and is an important source of functional diversity in mammalian cells (10). To date, the most studied and well-characterized function of protein lysine methylation is its contribution to the posttranslational modifications pattern of histones, regulating chromatin and gene expression (11). However, histones are not the only substrates of lysine methyltransferases, and there is growing evidence of nonhistone protein methylation events. Deregulation in protein methylation signaling may play a role in cancer initiation and progression, as well as therapeutic resistance (12, 13). Based on the reversibility and specific mechanisms underlying lysine methylation signaling, factors involved in such signaling have attractive characteristics as potential therapeutic targets (14, 15). The enzyme SMYD3 was the first lysine methyltransferase (KMT) to be linked to cancer etiology (16). It is overexpressed in various cancers, and its expression level frequently correlates with the proliferation and invasiveness of tumors (17). However, the molecular mechanisms underlying the oncogenic activity of SMYD3 remain elusive. In previous work, we identified a specific mechanism in which the SMYD3-mediated methylation of MAP3K2 potentiates the oncogenic KRAS-driven pathway in lung adenocarcinomas (18). Here, we observe that SMYD3 expression is highly upregulated in human SCLC, a cancer that is not induced by KRAS and nearly universally driven by inactivation of the *TP53* and *RB* tumor suppressor genes, thereby suggesting additional and unidentified targets in this context.

To study the implication of SMYD3 in SCLC, we have extended our observation to patient-derived tumor xenografts and mouse models. Using these models, we find that genetic depletion or pharmacologic inhibition of SMYD3 sensitizes cancer cells to alkylating therapeutics. To decipher the relevant molecular mechanisms of SMYD3 in SCLC, we performed biochemical screening, which identified the E3-ubiquitin ligase RING finger protein 113A (RNF113A) as a novel substrate. Notably, RNF113A was recently described as critical for the function of the activating signal cointegrator complex (ASCC) in dealkylation repair (19, 20). Biochemical assays indicate that RNF113A activity is regulated by phosphorylation in response to alkylating damage. Our proteomic analysis shows that SMYD3-mediated methylation of RNF113A prevents the binding of the phosphatase PP4, maintaining RNF113A active to sustain its role in the alkylation damage response. Finally, we observe that cells harboring active SMYD3–RNF113A signaling are more resistant to DNA alkylation damage. Therefore, this work reveals a new mechanism of cell tolerance to alkylation-based chemotherapy in SCLC, through the promotion of a dealkylation repair pathway induced by the elevation of RNF113A E3 ligase activity. We propose a rationale for targeting SMYD3 as a novel strategy to overcome the development of resistance in SCLC.

## RESULTS

### SMYD3 Is a Candidate Regulator of SCLC Susceptibility to Alkylating Chemotherapy

**Figure 1.**
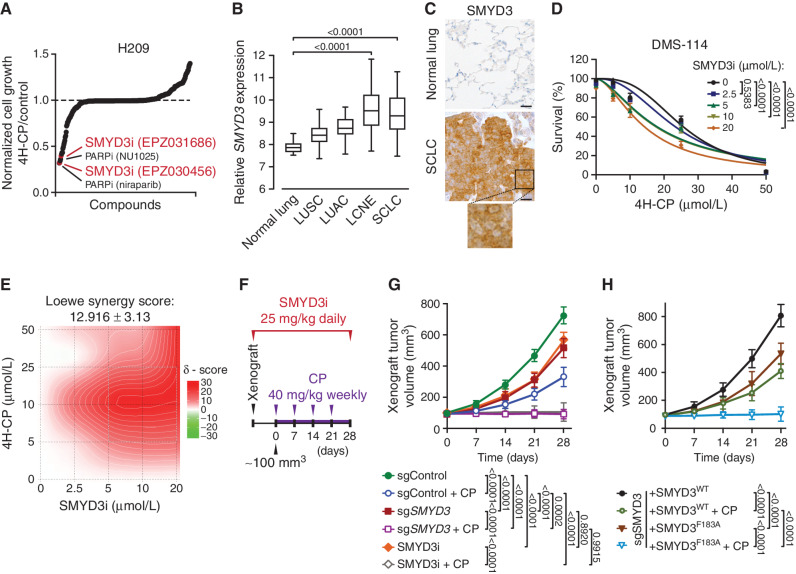
SMYD3 is a candidate regulator of SCLC susceptibility to alkylating chemotherapy. **A,** Synthetic lethality screening using a library composed of 285 characterized inhibitors, testing H209 SCLC cell sensitivity to alkylation damage by preactivated form of CP (4H-CP). Data represent the relative growth of H209 cells treated with a combination of 4H-CP (2.5 μmol/L) and different inhibitors (1 μmol/L each) compared with 4H-CP only (see Supplementary Table S1 and detailed description in the Methods). **B,***SMYD3* expression in different histologic subtypes of human lung cancer (GSE30219). The box plots show the distribution of *SMYD3* expression in indicated lung cancer subtypes: lung squamous cell carcinomas (LUSC; *n* = 61), lung adenocarcinomas (LUAC; *n* = 85), large cell neuroendocrine tumors (LCNE; *n* = 56), SCLC (*n* = 20), and in adjacent normal lung tissue (*n* = 14). *P* values were calculated using the Kruskal−Wallis test. **C,** Representative IHC staining of SMYD3 in normal human lung (*n* = 8) and tumor biopsies obtained from patients with confirmed SCLC (*n* = 24). A magnification is provided. All 24 analyzed SCLC biopsies showed positive nuclear and cytoplasmic SMYD3 staining with H-score >180 in 20 samples and H-score >100 in 4 samples. Scale bars, 50 μm. **D,** Analysis of DMS-114 SCLC cell line growth response to increasing concentrations of 4H-CP with or without SMYD3i (EPZ031686) at the indicated concentrations. The percentage of viable cells was normalized to control vehicle-treated cells. *P* values were calculated by two-way ANOVA with the Tukey test for multiple comparisons. Data are represented as nonlinear regression with mean ± SEM. **E,** Quantification of 4H-CP and SMYD3i combination treatment synergy using the Loewe model. Loewe synergy score was calculated from DMS-114 cell survival assays (as in **D**, SynergyFinder 2.0). **F,** Schematic of xenografts and CP treatment schedule using SCLC H1092 cells modified to express a control nontargeting sgRNA (sgControl) or a Cas9/sgRNA targeting SMYD3 (sg*SMYD3*) complemented or not using either WT or F183 inactive mutant SMYD3, or treated with SMYD3i (EPZ031686). The cells were grafted subcutaneously to immunocompromised *NOD. SCID-IL2Rg*^−/−^ (NSG) mice. **G,** Quantification of H1092 xenograft tumor volume (*n* = 5 mice, for each treatment group) is shown. Animals in control groups received placebo (vehicle) treatment. values were calculated by two-way ANOVA with Tukey testing for multiple comparisons. Data are represented as mean ± SEM. **H,** Quantification of H1092 xenograft tumor volume (*n* = 5 mice, for each treatment group) is shown. *P* values were calculated by two-way ANOVA with Tukey testing for multiple comparisons. Data are represented as mean ± SEM. In all panels, representative of at least three independent experiments is shown unless stated otherwise.

To identify clinically relevant factors that render SCLC vulnerable to commonly used chemotherapies, we performed two comparative cell-based screens using either cisplatin or 4-hydroperoxy-cyclophosphamide (4H-CP), an active metabolite of the clinically approved alkylating agent CP. These agents were combined and tested in the H209 SCLC cell line with a library of 285 characterized inhibitors covering ∼170 targets. The cisplatin screen did not lead to the identification of any novel candidates but confirmed previously described targets ameliorating cisplatin response in SCLC (Supplementary Fig. S1A; Supplementary Table S1), including inhibitors of EZH2 and the canonical DNA damage regulators (e.g., CHK1/2 and ATR; refs. 21, 22). In parallel, the 4H-CP screen revealed 10 compounds that elicited a 50% or greater increased cytotoxicity (Fig. 1A; Supplementary Table S1). Here again, these drugs included previously recognized chemosensitizers involved in DNA damage response (e.g., PARP1/2 inhibitors) and drug metabolism (e.g., GSH and ALDH1A1 inhibitors), validating the specificity of these screens (6, 23). However, among the top identified compounds potentiating anticancer activity of 4H-CP were two recently developed specific inhibitors of the SMYD3 lysine methyltransferase (EPZ031686 and EPZ030456; ref. 24; Fig. 1A; Supplementary Table S1). SMYD3 was not previously associated with SCLC tumorigenesis or response to chemotherapy, therefore potentially representing a novel regulator of SCLC pathogenies.

Computational analysis of publicly available gene-expression data revealed that *SMYD3* is particularly highly expressed in neuroendocrine lung cancer subtypes [SCLC and large cell neuroendocrine lung cancer (LCNE)] compared with other cancer subtypes and normal lung epithelium (Fig. 1B). Importantly, analysis of the human lung from single-cell RNA sequencing (RNA-seq; ref. 25) revealed the absence or low expression of SMYD3 in lung cell types, including pulmonary neuroendocrine cells (PNEC), likely cells of SCLC origin. This analysis also suggests that potential therapeutic inhibition of SMYD3 should not perturb normal lung and PNEC homeostasis (Supplementary Fig. S1B). Next, we confirmed high SMYD3 protein expression in SCLC using IHC staining of human cancer biopsies (Fig. 1C). Based on these observations, we postulated a role for SMYD3 in SCLC pathology and susceptibility to alkylating chemotherapy.

To further validate the SMYD3 role in mitigating alkylating chemotherapy efficacy, we tested a panel of SCLC cell lines (H209, H1092, and DMS-114) using the SMYD3 inhibitor EPZ031686 (SMYD3i) and two established alkylating drugs, 4H-CP and methyl methanesulfonate (MMS; Supplementary Fig. S1C–S1H). These experiments confirmed that SMYD3 suppression increases the sensitivity of multiple SCLC cell lines to both alkylating agents, suggesting a potential common mechanism. Next, using the dose–response matrix of SMYD3i and 4H-CP in the DMS-114 cell line, we calculated drug combination effects, which demonstrated synergistic efficacy (Loewe score of 12.9; ref. 26; Fig. 1D and E; Supplementary Fig. S1I). Finally, we performed xenograft tumor growth studies using H1092 SCLC cells either depleted for SMYD3 via CRISPR/Cas9 or treated with SMYD3i, with and without CP treatment (Fig. 1F; Supplementary Fig. S1J). We noted that the ablation and inhibition of SMYD3 in SCLC cells partially delayed tumor growth (Fig. 1G), whereas additional CP treatment significantly halted tumor growth or caused some tumors to regress in size. On the contrary, the control xenograft tumors expanded rapidly, and CP monotherapy was only modestly effective in delaying tumor growth (Fig. 1G). To fully validate that the methyltransferase activity of SMYD3 is required for its function in SCLC, we performed similar xenograft assays with engineered H1092 depleted for SMYD3 and complemented with either wild-type (WT) or F183A catalytically inactive SMYD3 (Fig. 1H; Supplementary Fig. S1J). Remarkably, we noticed that the inactive form of SMYD3 was unable to induce cellular resistance to CP therapy compared with cells complemented with the WT SMYD3 (Fig. 1H), suggesting that the methyltransferase activity of SMYD3 is required to promote SCLC cells’ resistance to CP *in vivo.* Together, these results support a model where the function of SMYD3 is involved in SCLC response to alkylation therapy.

### Identification of RNF113A as a Novel Methylated Substrate of SMYD3

**Figure 2.**
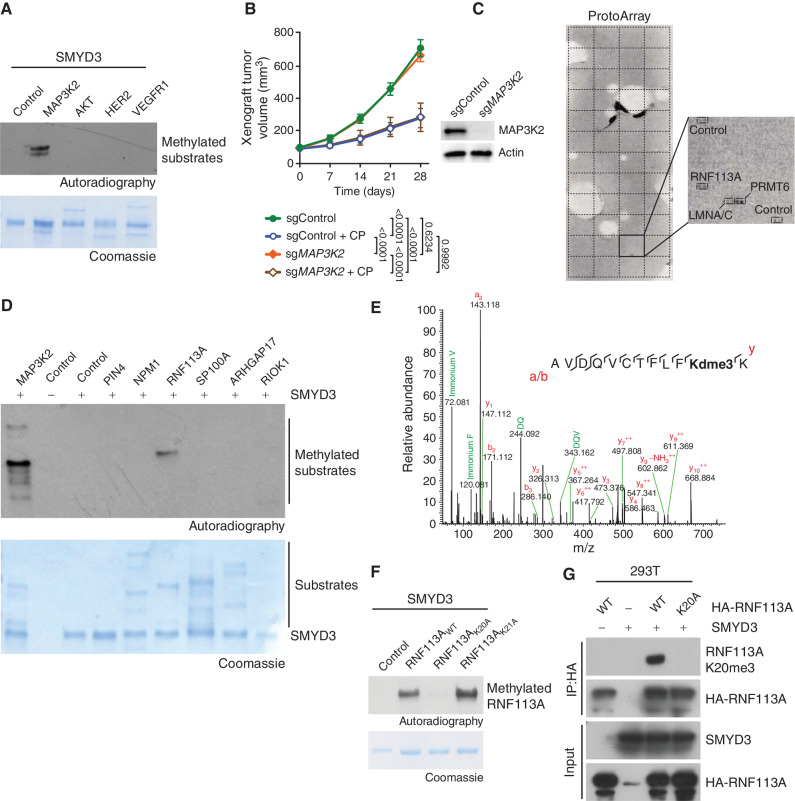
Identification of RNF113A as a novel methylated substrate of SMYD3. **A,** Recombinant SMYD3 was used for *in vitro* methylation reactions using radiolabeled S-adenosylmethionine and potential substrates. Top, autoradiogram of the methylation assay. Bottom, Coomassie stain of proteins in the reaction. **B,** H1092 SCLC cells were modified to express Cas9/sgRNA targeting MAP3K2 (sg*MAP3K2*) or control nontargeting sgRNA (sgControl). The cells were grafted subcutaneously to immunocompromised NSG mice. Once tumor volume reached 100 mm^3^, indicated animal groups were treated with CP and control groups received placebo (vehicle) treatment. Quantification of xenograft tumor volume growth is shown (*n* = 5 mice for each treatment group). *P* values were calculated by two-way ANOVA with the Tukey test for multiple comparisons. Data are represented as mean ± SEM. Representative immunoblot analysis of indicated cell lysates is shown. Tubulin is used as a loading control. **C,** Representative image showing recombinant SMYD3 *in vitro* methylation reaction on protein arrays (ProtoArray—containing more than 9,500 potential substrates) using radiolabeled S-adenosylmethionine as a methyl donor. Magnification shows the signals identified in square 43 of the Protoarray, corresponding to the indicated spotted proteins and controls. **D,***In vitro* methylation assay as in **A** using potential substrates identified by ProtoArray. Top, autoradiogram of the methylation assay. Bottom, Coomassie stain of proteins in the reaction. **E,** Identification of RNF113A K20 trimethylation by bottom-up MS-based proteomic analysis of RNF113A methylated *in vitro* by SMYD3. Note that deuterated SAM was used as a methyl donor. **F,***In vitro* methylation assay as in **A** with recombinant SMYD3 and WT RNF113A as well as K20A or K21A mutant proteins. Top, autoradiogram of the methylation assay. Bottom, Coomassie stain of proteins in the reaction. **G,** Detection of RNF113A methylation in 293T cells using the RNF113A K20me3 antibody after ectopic expression of SMYD3 and WT or K20A mutant RNF113A. In all panels, a representative of at least three independent experiments is shown unless stated otherwise.

To identify the mechanisms of SMYD3-mediated response to alkylating agents in SCLC, we evaluated targets previously associated with SMYD3 methyltransferase activity using *in vitro* assays (17). Consistent with our previous works (18), our analysis indicated that SMYD3 can methylate MAP3K2 but not VEGFR1, HER2, nor AKT (Fig. 2A). We previously identified an oncogenic activity of SMYD3 mediated by MAP3K2 methylation, promoting KRAS-driven lung adenocarcinoma tumorigenesis through ERK1/2 oncogenic activation (18). Because SCLC is not characterized by mutations or aberrant activity of canonical RAS signaling (27), and because MAP3K2 can alternatively regulate other downstream signaling such as the MEK5–ERK5 pathway recently implicated in SCLC pathogenesis (28–30), we aimed to analyze potential SMYD3-dependent MAP3K2 oncogenic signaling in SCLC cells. We tested whether MAP3K2 specifically affects the response of human SCLC to CP *in vivo.* To that end, we performed a xenograft tumor growth study using H1092 SCLC cells depleted for MAP3K2 and treated with CP. We noted that the ablation of MAP3K2 cells had no effect on tumor growth or response to alkylating therapy (Fig. 2B). Altogether, these observations indicated that the phenotype associated with loss of SMYD3 in SCLC is independent of the SMYD3–MAP3K2 pathway or other MAP3K2-related signaling mechanisms.

We therefore hypothesized that an unknown substrate may be responsible for SMYD3 oncogenic function in SCLC. To identify this new potential substrate, we performed an unbiased high-throughput approach using a human protein microarray (ProtoArray) radiolabeled methylation assay (Fig. 2C; ref. 31). Among approximately 9,500 potential candidates, we identified 22 proteins as being methylated in the presence of SMYD3, including the previously characterized substrate MAP3K2 (Supplementary Table S2). A portion of the hits were likely false positives and were discarded, due to being automethylated lysine methyltransferases (such as PRMT6, for example). We further tested several candidates using *in vitro* methylation assays and identified the E3 ubiquitin ligase RNF113A as a genuine substrate methylated by SMYD3 *in vitro* (Fig. 2D). Because RNF113A is a protein involved in alkylation damage repair (19, 20), we decided to further characterize the potential link between its methylation and SMYD3's implication in cell sensitization to alkylation-based chemotherapy. Mass spectrometry–based proteomic analyses revealed specific trimethylation of RNF113A at lysine K20 (RNF113A K20me3; Fig. 2E). Using *in vitro* radiolabel-based methylation assays with purified SMYD3 and RNF113A, we confirmed that K20 is the single SMYD3-mediated methylation site on RNF113A, as the K20A substitution but not the neighboring K21A mutant completely abrogated methylation induced by SMYD3 (Fig. 2F). Furthermore, we verified that the SMYD3 inhibitor EPZ031686 efficiently blocked RNF113A methylation *in vitro* (Supplementary Fig. S2A).

In order to confirm the presence of RNF113A methylation in cells, we raised an antibody against RNF113A K20me3, which demonstrated high specificity against its antigen (Supplementary Fig. S2B and S2C). Using this RNF113A K20me3–specific antibody, we found that ectopically expressed WT RNF113A can be methylated by SMYD3 in human 293T cells (Fig. 2G).

Altogether, our data strongly support RNF113A as a novel methylated substrate of SMYD3.

### SMYD3–RNF113A Methylation Signaling in SCLC Cell Lines

We aimed to investigate if this newly discovered SMYD3–RNF113 methylation event is physiologic and can be detected in cells, notably in relevant SCLC cells where it could explain the role of SMYD3 in alkylation damage sensitivity. First, we observed endogenous methylation of RNF113A in HeLa cells and the specific loss of RNF113A methylation upon inducible genetic repression of SMYD3 (Supplementary Fig. S3A). We further noted that the SMYD3i, in a concentration-dependent manner, was able to repress RNF113A methylation in HeLa cells (Supplementary Fig. S3B). Therefore, we decided to characterize further RNF113A methylation in SCLC.

**Figure 3.**
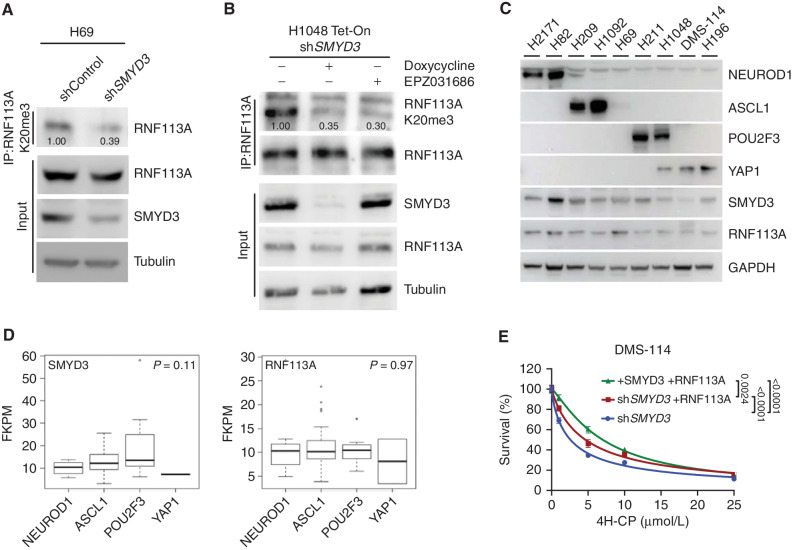
SMYD3–RNF113A methylation signaling in SCLC cell lines. **A,** Immunodetection of endogenous RNF113A K20me3 following immunoprecipitation of total RNF113A in SCLC H69 cells transduced with shRNA targeting *SMYD3* (sh*SMYD3*) and a control nontargeting shRNA (shControl). Tubulin was used as a loading control. **B,** Immunoblot analysis with indicated antibodies as in **A** of H1048 SCLC cells expressing doxycycline-inducible sh*SMYD3* or treated with SMYD3i (EPZ031686). Tubulin was used as a loading control. **C,** Immunoblot analysis with indicated antibodies using lysates obtained from human SCLC cell lines representing all four molecular subtypes (NAPY) classified by expression of specific markers (NEUROD1^+^; ASCL1^+^; POU2F3^+^; YAP1^+^). GAPDH was used as a loading control. **D,***SMYD3* and *RNF113A* expression in human samples representing different molecular SCLC subtypes. Boxes represent 25th to 75th percentiles; whiskers: 10% to 90%; center line: median. *P* values were calculated by the Kruskal–Wallis test. Analyses were performed using FPKM data for each specified gene obtained from ref. 34. NAPY SCLC subclassification was based on the original classification from ref. 32. **E,** Analysis of DMS-114 SCLC cell line growth response to increasing concentrations of 4H-CP. Cells were transduced with doxycycline-inducible sh*SMYD3* and complemented with the expression of RNF113A or both SMYD3 and RNF113A. The percentage of viable cells under each condition was normalized to vehicle-treated (control) cells. Each condition represents the mean of three technical replicates from two independent experiments. *P* values were calculated by two-way ANOVA with the Tukey test for multiple comparisons. Data are represented as nonlinear regression with mean ± SEM. In all panels, representative of at least three independent experiments is shown unless stated otherwise. The numbers below the immunoblot lines represent the relative signal quantification (see also Supplementary Table S5).

We observed that endogenous RNF113A is trimethylated at K20 in both H69 and H1048 SCLC cells (Fig. 3A and B). Remarkably, this methylation event was significantly abrogated upon SMYD3 genetic depletion or pharmacologic inhibition in these two cell lines, which are representative of two different SCLC subtypes. Indeed, SCLC has been recently classified into different subtypes in regard to the exclusive expression of four putative driver transcription factors, NEUROD1, ASCL1, POU2F3, and YAP1 (named NAPY classification; refs. 32, 33). We collected a panel of different SCLC cell lines representing the four SCLC subtypes and determined the expression of SMDY3 and RNF113A. Interestingly, both proteins were detected in all cell lines without a clear subtype specificity (Fig. 3C). To further our study, we performed bioinformatic analysis on available SCLC RNA-seq data (34) and determined SMYD3 and RNF113A expression within the four different NAPY subtypes from primary SCLC samples. Here again, no specific enrichment for a given subtype was identified, suggesting that the potential SMYD3–RNF113A methylation signaling may be relevant within the majority of SCLC (Fig. 3D; Supplementary Fig. S3C). Next, we sought to evaluate the correlation of SMYD3 expression level with cellular response to CP. We used publicly available data (Broad Institute and NCI's Cancer Target Discovery and Development Network), which revealed that SMYD3 expression levels correlate with increased resistance to CP (Pearson correlation coefficient ρ = 0.48; Supplementary Fig. S3D). To relate how SMYD3/CP compares with other known factors regulating the response to chemotherapy in SCLC, we performed a similar analysis for EZH2 and SLFN11, which have been described to drive resistance to platinum-based agents in SCLC (35). Our analysis shows that neither EZH2 nor SLFN11 levels show a significant degree of correlation with resistance to platinum-based chemotherapy (ρ = 0.16 and ρ = −0.26, respectively; Supplementary Fig. S3E). To further test the relevance of SMYD3–RNF113A signaling, we then took advantage of the SCLC cell line DMS-114, originating from a chemotherapy-naïve patient with relatively low expression of RNF113A and SMYD3 compared with other SCLC cell lines (see Fig. 3C). We engineered DMS-114 cells for the differential expression of SMYD3 and RNF113A and tested their response to alkylation damage (Supplementary Fig. S3F). We found that the overexpression of RNF113A in the absence of SMYD3 partially increased resistance to 4H-CP and MMS (Fig. 3E; Supplementary Fig. S3G). Strikingly, combined overexpression of RNF113A and SMYD3 significantly induced cellular tolerance to both 4H-CP and MMS (Fig. 3E; Supplementary Fig. S3G).

Altogether, these data indicate that RNF113A is a bona fide substrate of SMYD3 in SCLC cells and suggest that the SMYD3–RNF113A signaling may participate in SCLC resistance to alkylation-based chemotherapy.

### RNF113A Is a Phosphoprotein and Its Methylation Repels the Phosphatase PP4

RNF113A is a protein involved in alkylation damage repair, in which its E3 ubiquitin ligase activity promotes the proper recruitment of the ASCC with the dealkylase ALKBH3 (19). This process is mediated by the direct binding of the subunit ASCC2 to K63-linked ubiquitin chains formed by RNF113A in nuclear speckle bodies (19). However, how RNF113A is activated and regulated remains unclear, and we sought to determine whether the regulation of RNF113A activity by SMYD3 methylation may be a potential mechanism of tumor resistance to alkylation damage.

**Figure 4.**
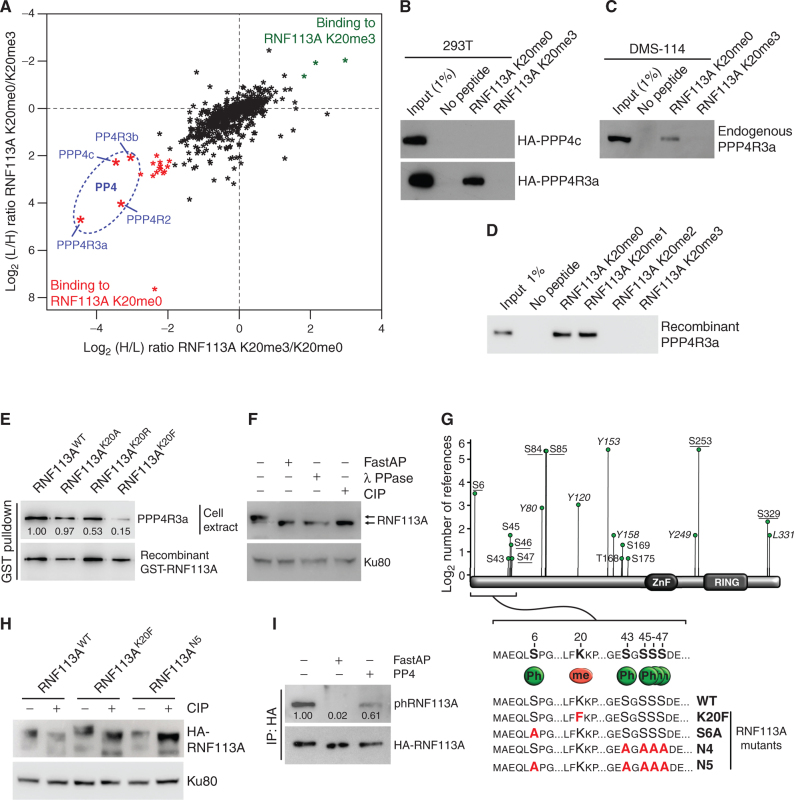
RNF113A is a phosphoprotein and its methylation repels the phosphatase PP4. **A,** SILAC quantitative proteomics analysis of proteins that interact with RNF113A K20me0 and RNF113A K20me3 peptides. Data represent two independent experiments (forward and reverse directions). Proteins are plotted by their SILAC ratios in the forward (*x*-axis) and reverse (*y*-axis) experiments. Specific interactors of RNF113A K20me0 reside in the lower left quadrant. The four PP4 complex subunits are circled in blue. L/H, light over heavy fraction ratio. **B,** 293T cell extracts ectopically expressing HA-tagged PPP4R3a and PPP4c subunits were used for pulldowns with the indicated RNF113A peptides, followed by immunoblot analysis using the indicated antibodies. **C,** Immunoblot analysis of endogenous PPP4R3A following pulldowns with indicated RNF113A peptides using SCLC DMS-114 cell extract. **D,** Immunoblot analysis of recombinant PPP4R3A following pulldowns with the indicated RNF113A peptides. **E,** Immunoblot analysis of endogenous PPP4R3A pulldown using GST labeled recombinant RNF113A WT, K20A, K20R, and K20F mutants. **F,** Phosphorylation-dependent mobility shift of RNF113A on SDS-PAGE immunoblotting (indicated by arrows). HeLa cell extracts were treated with λ phosphatase (λ PPase), FastAP thermosensitive alkaline phosphatase (Fast AP), or calf intestinal alkaline phosphatase (CIP). Ku80 was used as a loading control. **G,** Identification of potential RNF113A phosphorylation sites based on the Phosphosite Plus references (*y*-axis) and confirmed by two independent mass spectrometry analyses (underlined residues; see also Supplementary Table S4). The schematic shows the sequence surrounding the methylated K20 and PPP4R3a binding motif (FxxP). Summary of phosphorylation and methylation site mutants of RNF113A generated in this study (bottom). **H,** Immunoblot confirmation of phosphorylation-dependent mobility shift of the indicated RNF113A mutants expressed in HeLa cells with or without CIP treatment. Ku80 was used as a loading control. **I,** Immunoblot analysis of RNF113A dephosphorylation assays using HA-RNF113A purified from HeLa cells, with either FastAP or PP4 phosphatases treatment followed by immunoblot analysis using a phospho-CDK-consensus motif antibody. In all panels, representative of at least three independent experiments is shown unless stated otherwise. The numbers below the immunoblot lines represent the relative signal quantification (see also Supplementary Table S5).

Lysine methylation predominantly affects signaling by modulating protein–protein interactions (36). Therefore, to identify RNF113A methylation-sensitive interactions specifically altered by SMYD3 activity, we performed a peptide pulldown coupled to stable isotope labeling of amino acid in cell culture (SILAC) quantitative MS-based proteomic analysis using unmodified or trimethylated versions of RNF113A K20. We identified a number of proteins that were strongly associated with RNF113A K20me0 but which were repelled by RNF113A K20me3 (Supplementary Table S3). Interestingly, among the most confident hits identified in three independent experiments were proteins belonging to the serine/threonine–protein phosphatase complex PP4. These included the catalytic subunit PPP4c, the chaperone PPP4R2, as well as the substrate-specific binders PPP4R3a and PPP4R3b (Fig. 4A). PPP4R3a appeared to be the subunit most strongly associated with unmethylated RNF113A, consistent with its function in PP4 substrate recognition. As a confirmation, we observed that ectopically expressed PPP4R3a, but not PPP4c, bound to the unmethylated RNF113A peptide and that the trimethylation of lysine K20 abrogated this interaction (Fig. 4B). We validated that endogenous PPP4R3a from the SCLC cell line DMS-114 bound to unmethylated but not trimethylated RNF113A K20 peptide (Fig. 4C). In addition, we found that this interaction is direct, as recombinant PPP4R3a was able to bind to both unmethylated and monomethylated RNF113A K20 peptides and was repelled by either di- or trimethylation of lysine K20 (Fig. 4D). Interestingly, a recent study on PPP4R3a identified the specific binding motif of this phosphatase subunit as “FxxP,” where the first x is preferentially a lysine (37). This motif matches the “FKKP” sequence of RNF113A, with the first K being lysine K20. Therefore, trimethylation of lysine K20 by SMYD3 within the FxxP recognition motif is likely to interfere with the binding capacity of the phosphatase complex. Indeed, the replacement of K20 with a bulkier, more hydrophobic amino acid such as phenylalanine efficiently blocked PPP4R3a interaction, mimicking the effect of lysine K20 trimethylation by SMYD3, whereas mutation of RNF113A K20 into alanine did not affect this binding (Fig. 4E). Moreover, pulldown assays of endogenous PPP4R3a from 293T cell extracts using ectopic expression of RNF113A demonstrated that PPP4R3a efficiently bound to full-length WT RNF113A, but significantly less to the K20F mutant (Supplementary Fig. S4A).

While performing SDS-PAGE electrophoresis, we observed that RNF113A migrated higher than its theoretical molecular weight, and that preincubation of the cellular extract with different commercially available phosphatases restored its expected molecular weight (Fig. 4F). Moreover, mass spectrometry analysis of RNF113A purified from HeLa cells, together with information collected from the PhosphoSitePlus database (38), demonstrated that RNF113A is phosphorylated at several serine residues surrounding the binding site of the PP4 phosphatase (Fig. 4G; Supplementary Table S4). We observed that RNF113A K20F mutant migrated even more slowly than the WT, whereas a mutant where the five N-terminal serines surrounding the binding sites of PP4 are substituted for alanine (RNF113A N5) migrated at the same size as RNF113A treated with CIP phosphatase (Fig. 4H; Supplementary Fig. S4B). Remarkably, the sequence context of serine 6 corresponds to the cyclin-dependent kinases motif “(K/H)pSP,” and we observed that purified PP4 phosphatase efficiently dephosphorylates RNF113A using a phospho-CDK pan-substrate antibody (Fig. 4I).

Therefore, the identified specific interaction of RNF113A with the PP4 complex suggests that RNF113A is a phosphoprotein, and that SMYD3 and PP4 may regulate RNF113A functions through control of its phosphorylation levels (Supplementary Fig. S4C).

### Methylation–Phosphorylation Cross-talk Regulation of RNF113A Affects Its E3 Ligase Activity

**Figure 5.**
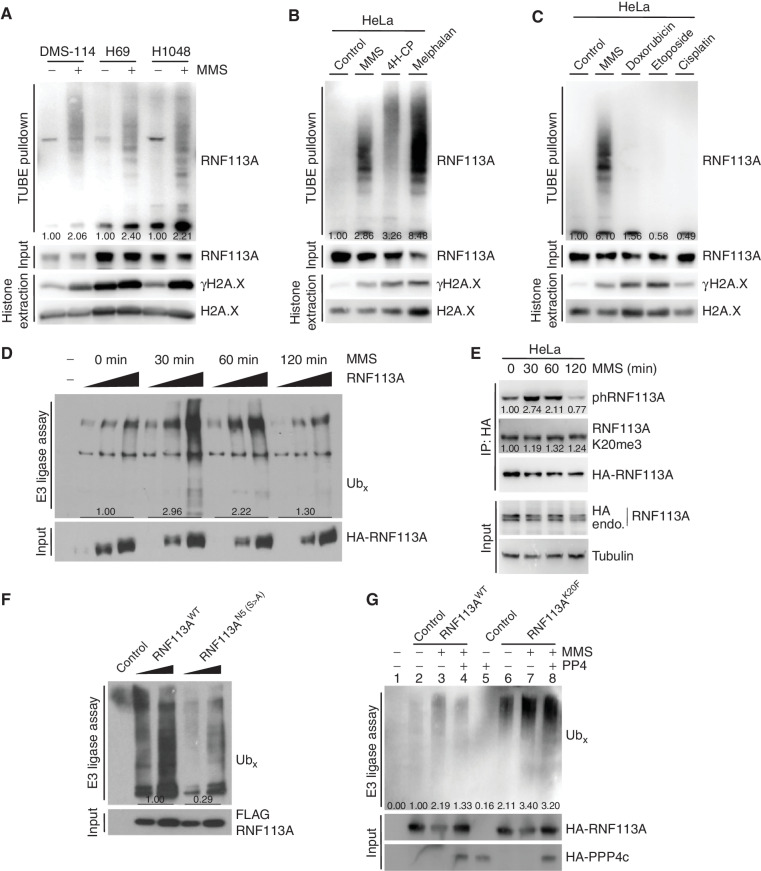
Methylation–phosphorylation cross-talk regulation of RNF113A affects its E3 ligase activity. **A,** Immunodetection of autoubiquitinated RNF113A after TUBE pulldowns using DMS-114, H69, and H1048 SCLC cell extracts following treatment with MMS. γH2A.X is shown as a marker of DNA damage induction. **B,** Immunodetection of autoubiquitinated RNF113A after TUBE pulldowns as in **A**, using HeLa cells extracts after treatment with different alkylating agents. γH2A.X is shown as a marker of DNA damage induction. **C,** Immunodetection of autoubiquitinated RNF113A after TUBE pulldowns as in **A**, using HeLa cells extracts after treatment with MMS versus different nonalkylating DNA-damaging agents. γH2A.X is shown as a marker of DNA damage induction. **D,***In vitro* E3 ubiquitin ligase assays were performed with FLAG-HA-RNF113A purified from HeLa S3 cells with or without prior MMS treatment for the indicated duration. This was followed by immunoblot analysis with the indicated antibodies. **E,** Immunoblot analysis of total, phosphorylated, and methylated RNF113A immunoprecipitated from HeLa cells stably expressing HA-RNF113A, with or without prior MMS treatment for the indicated duration. **F,***In vitro* E3 ubiquitin ligase assays were performed with WT or N5-mutant forms of RNF113A purified from HeLa S3 cells. This was followed by immunoblot analysis with the indicated antibodies. **G,***In vitro* E3 ubiquitin ligase assays were performed as in **F** using WT or K20F-mutant forms of RNF113A purified from HeLa S3 cells treated with or without prior MMS treatment. Where indicated the E3 enzyme was preincubated with PP4 phosphatase. In all panels, representative of at least three independent experiments is shown unless stated otherwise. The numbers below the immunoblot lines represent the relative signal quantification (see also Supplementary Table S5).

RNF113A was recently described as an E3 ubiquitin ligase induced by alkylation damage promoted by the alkylating agent MMS and involved in dealkylation repair response (19). However, how RNF113A activity is regulated was not understood, and we hypothesized that RNF113A phosphorylation could be critical for its E3 ligase activity. Because recombinant RNF113A produced in bacteria is inactive (19), further suggesting the importance of RNF113A posttranslational modifications, we purified RNF113A from engineered HeLa S3 cells stably expressing HA-FLAG-RNF113A at a level comparable to endogenous RNF113A (Supplementary Fig. S5A). We first confirmed by *in vitro* E3 ubiquitin ligase assays that RNF113A is able to efficiently form polyubiquitin chains and that pretreatment of the cells with the alkylating agent MMS efficiently stimulates this activity (Supplementary Fig. S5B). In addition, we observed that alkylation damage stimulates RNF113A activity using another method by monitoring RNF113A autoubiquitination (Fig. 5A). Specifically, isolation of endogenously ubiquitinated proteins using tandem ubiquitin-binding element (TUBE) beads to isolate endogenously ubiquitinated proteins from three different SCLC cell lines (DMS-114, H69, and H1048) showed increased RNF113A autoubiquitination during MMS (Fig. 5A) or 4H-CP (Supplementary Fig. S5C) alkylation stress. We confirmed that RNF113A alone mediates its ubiquitination, as deletion of the catalytic RING domain of RNF113A resulted in a loss of the autoubiquitination signal upon MMS treatment (Supplementary Fig. S5D). Finally, using exogenous His-tagged ubiquitin expression in cells followed by ubiquitinated protein enrichment using Ni-NTA beads also demonstrated the activation of RNF113A upon alkylation damage (Supplementary Fig. S5E).

The fact that SMYD3 inhibition induces alkylation but not cisplatin sensitivity suggested damaging agent selectivity of this pathway. Indeed, RNF113A is specifically activated by alkylating agents such as MMS, 4H-CP, and melphalan, but not by other DNA damage agents used in chemotherapy such as cisplatin, etoposide, or doxorubicin (Fig. 5B and C). Concordant with these results, DMS-114 cells engineered to overexpress RNF113A and SMYD3 did not gain resistance to cisplatin treatment (Supplementary Fig. S5F). Next, we analyzed the dynamics of MMS stimulation by performing *in vitro* E3 ubiquitin ligase assays with RNF113A purified from cells during a time course following alkylation damage. Strikingly, we observed an acute activation of RNF113A after 30 minutes of treatment followed by a significant decrease after 1 hour, and a return to basal activity after 2 hours of MMS treatment (Fig. 5D). Based on our hypothesis, we anticipated that RNF113A phosphorylation would follow the dynamics of RNF113A activity. Remarkably, the peak of RNF113A activity matched a significant increase of RNF113A phosphorylation at 30 minutes, followed by a decrease to basal level at 120 minutes, whereas the overall methylation of RNF113A remained unchanged (Fig. 5E).

The rapid inactivation and decrease in phosphorylation of RNF113A indicated a possible regulation by a phosphatase such as PP4. Therefore, we next aimed at deciphering if RNF113A activity is regulated by its phosphorylation status and if SMYD3 or PP4 could regulate RNF113A E3 activity. We used different phosphorylation mutants targeting the N-terminus of RNF113A (see Fig. 4G) and found that the RNF113A N5 mutant had significantly lower E3 ligase activity upon MMS stimulation (Fig. 5F). TUBE assays confirmed that this phospho-mutant was significantly less capable of autoubiquitination, confirming its reduced activity compared with WT RNF113A (Supplementary Fig. S5G). Furthermore, we took advantage of the RNF113A K20F mutant, which mimics RNF113A methylation by SMYD3 and blocks PP4 interaction, and anticipated that this mutant would be constitutively phosphorylated and activated once stimulated. Indeed, we observed that the RNF113 K20F mutant was more active and autoubiquitinated than WT RNF113A by TUBE and His-Ub pulldown assays, and a significantly higher phosphorylation of RNF113A K20F mutant was observed after MMS stimulation compared with WT RNF113A (Supplementary Fig. S5H–S5J). Of note, we observed that the phosphorylation level of RNF113A K20F mutant without MMS induction was increased at baseline compared with WT RNF113A, suggesting that PP4 dephosphorylation may actively participate in downregulating RNF113A in the absence of damage. Remarkably, we found that this difference of phosphorylation positively correlated with an increase in E3 ligase activity of RNF113A K20F mutant compared with WT upon MMS stimulation (Fig. 5G; wells 3 vs. 7). In addition, although purified PP4 was able to repress WT RNF113A E3 ligase activity *in vitro*, the phosphatase was relatively inefficient in inactivating RNF113A K20F, likely due to impaired binding to this mutant (Fig. 5G, compare wells 3 vs. 4 and 7 vs. 8). Finally, we confirmed that the phosphorylation level of RNF113A is ultimately responsible for its E3 ligase activity, as the K20F/N5 double mutant had a similar autoubiquitination activity compared with the N5 mutant (Supplementary Fig. S5K).

Altogether, these data demonstrate that RNF113A is a phospho-regulated E3 ligase that is specifically activated in response to alkylation damages. Furthermore, these data indicate that the E3 ligase activity of RNF113A is regulated by phosphorylation and that SMYD3-mediated methylation blocks PP4 and induces higher RNF113A phosphorylation levels.

### RNF113A Regulation Affects Its Function in DNA Dealkylation Repair

**Figure 6.**
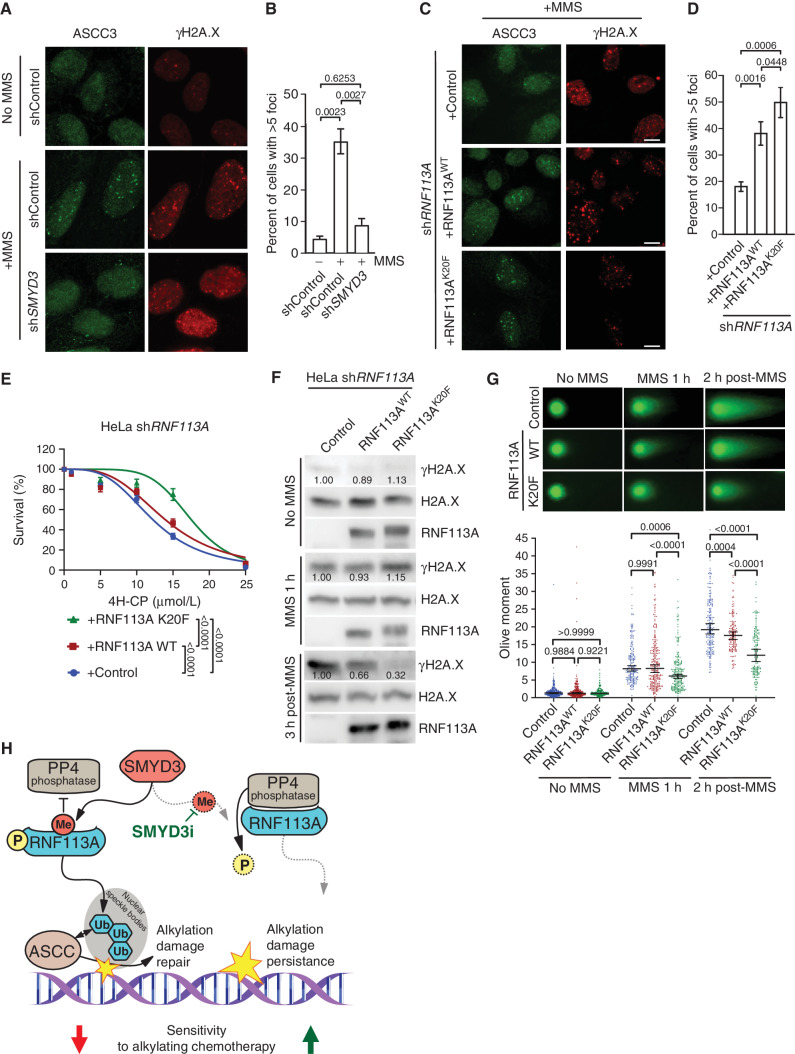
RNF113A regulation affects its function in DNA dealkylation repair. **A,** Representative images of MMS-induced ASCC3 foci in sh*SMYD3* or shControl U2OS cells with or without prior MMS. Foci were monitored by immunofluorescent staining of ASCC3 (left) and the DNA damage marker γH2A.X (right). **B,** Quantification of ASCC3 foci formation from **A**. A minimum of 100 cells were quantified for each experimental condition. *P* values were calculated by a two-tailed unpaired Student *t* test, and error bars represent mean ± SD. **C,** Representative images of MMS-induced ASCC3 foci as in **A** in U2OS cells reconstituted with either RNF113A WT or K20F mutant after endogenous RNF113A knockdown by shRNA (sh*RNF113A*). **D,** Quantification of ASCC3 foci formation from **C**. A minimum of 100 cells were counted for each experimental condition. *P* values were calculated by two-tailed unpaired Student *t* test, and error bars represent mean ± SD. **E,** Engineered HeLa cell viability assays using different concentrations of 4H-CP. Cells were stably transduced with inducible shRNA *RNF113A* (sh*RNF113A*) and reconstituted with either WT RNF113A or the K20F mutant. The percentage of viable cells under each condition was normalized to untreated cells. Each condition represents the mean of three technical replicates from two independent experiments. *P* values were calculated by two-way ANOVA with Tukey testing for multiple comparisons. Data are represented as nonlinear regression with mean ± SEM. **F,** Immunoblots with indicated antibodies of cell lysates as in **E** with or without MMS treatment for the indicated duration and with or without the indicated recovery duration. **G,** Neutral comet assays depicting DNA double-stranded break repair in engineered HeLa cells as in **F** with representative examples of comet tails (top) and Olive moment quantification (bottom). A minimum of 150 comets were analyzed for each condition. *P* values were calculated by two-way ANOVA with the Tukey test for multiple comparisons. Data are represented as median with 95% CI. **H,** Model of SMYD3 participation in coordinating SCLC response to alkylating therapy through RNF113A methylation. In SCLC overexpressing SMYD3 (left), RNF113A activation leads to efficient dealkylation repair by ASCC and loss of cancer sensitivity to alkylation-based chemotherapy. Specific SMYD3 inhibition allows for RNF113A inactivation by PP4 and prevents RNF113A-mediated alkylation damage response, leading to sustained tumor growth inhibition by alkylating chemotherapy (right). In all panels, representative of at least three independent experiments is shown unless stated otherwise. The numbers below the immunoblot lines represent the relative signal quantification (see also Supplementary Table S5).

Because RNF113A acts upstream of the alkylation damage repair pathway by recruiting the ASCC complex, we reasoned that an upregulated response to alkylation damage may promote cancer cell tolerance to alkylating agents and explained the impact of SMYD3 in our SCLC and xenograft models. Previous work suggested that the alkylating agent MMS induced ASCC complex recruitment to nuclear speckle bodies and that RNF113A E3 ligase activity was necessary for this process (19, 20). To investigate the functional link between the SMYD3–RNF113A regulation and the efficient recruitment of the ASCC complex, we examined the localization of the main subunit of the complex, ASCC3, within foci induced by alkylation damage. First, we validated that MMS induced recruitment of the ASCC complex in U2OS cells (Figs. 6A and B). Remarkably, we observed a significant decrease of ASCC3 foci number in U2OS cells repressed for SMYD3 (Fig. 6A and B). We should note that we used U2OS cells for ASCC foci analysis because they are significantly more adherent relative to SCLC cells and hence amenable to high-resolution microscopy. Notably, U2OS, HeLa, and H1048 SCLC cells have fairly comparable levels of SMYD3 and RNF113A (Supplementary Fig. S6A). We then generated U2OS cells depleted for endogenous RNF113A by shRNA and rescued with either WT RNF113A, phospho-mutants S6A and N5, or methyl-mimetic K20F (Supplementary Fig. S6B–S6D). Consistent with a loss of RNF113A E3 ligase activity previously observed, reconstitution with the RNF113A N5 mutant resulted in decreased ASCC3 foci formation upon alkylation stress, suggesting the importance of RNF113A phosphorylation for its function (Supplementary Fig. S6E and S6F). In contrast, the K20F mutant had increased ASCC3 foci formation, consistent with its increased activity as an E3 ligase (Fig. 6C and D). Moreover, we noticed that the intensity of ASCC3 foci formed upon MMS treatment was significantly higher for the RNF113A K20F mutant compared with its WT counterpart, suggesting a more robust response to alkylation damage (Supplementary Fig. S6G and S6H).

Alkylating agents are an important component of the chemotherapy repertoire for various cancers. Thus, we decided to examine the impact of RNF113A regulation on cell sensitivity to alkylating chemotherapy. First, we confirmed that HeLa cells treated with SMYD3i were sensitized to alkylation damage (Supplementary Fig. S6I). Next, we generated HeLa cells with sh*RNF113A* and reconstituted them with either RNF113A WT or K20F, and observed a clear increased resistance of RNF113A K20F–containing cells to both 4H-CP and MMS treatments (Fig. 6E; Supplementary Fig. S6J). Because our previous foci analyses indicated increased recruitment of the ASCC alkylation damage repair complex corresponding to a decrease of DNA double-stranded break signaling (see γH2AX foci in Fig. 6A and C), we next sought to determine cellular capacity to repair DNA damage by monitoring levels of γH2AX before, during, and after MMS treatment. In comparison with cells depleted of RNF113A, we observed a moderate reduction of γH2A.X 3 hours after MMS treatment in cells reconstituted with WT RNF113A, suggesting an increased efficacy of cells to repair alkylated damage (Fig. 6F). Remarkably, the RNF113A K20F was even more efficient, as suggested by the lower levels of γH2AX, concordant with a more active form of the protein and a better induction of the alkylation damage repair (Fig. 6F). Finally, we performed neutral comet assays to monitor DNA double-stranded breaks after induction of alkylation damage. Although no differences were observed without MMS treatment between the three conditions, we already noted a decreased overall “olive moment” (representative of the head to tail intensity ratio of the comet) with RNF113A K20F after alkylation compared with control and WT RNF113A (Fig. 6G). This phenotype was even more pronounced 2 hours after recovery, confirming the better efficacy of RNF113A K20F mutant to recruit the proper repair machinery upon MMS-induced alkylation damage (Fig. 6G).

Therefore, our data demonstrate that a more active form of RNF113A, notably the RNF113A mutant mimicking the methylation by SMYD3, leads to a better activation of the ASCC damage repair pathway and promotes increased cellular resistance to alkylating damage and DNA breaks. Altogether, our study depicts a model where overexpression of SMYD3 increases RNF113A E3 ligase function and DNA alkylation repair, and blocking SMYD3 methyltransferase activity using genetic or pharmacologic repression could sensitize cells to alkylation-based chemotherapy (Fig. 6H).

### SMYD3 Inhibition Sensitizes SCLC to Alkylating Agents *In Vivo*

**Figure 7.**
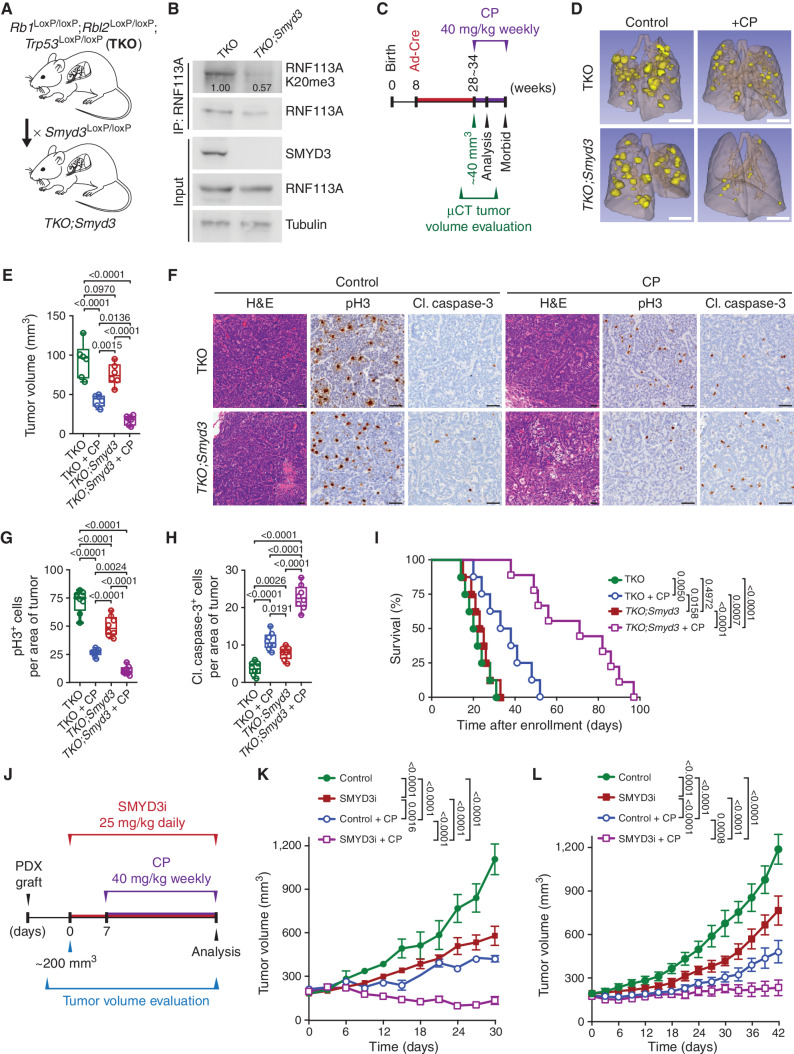
SMYD3 inhibition sensitizes SCLC to alkylating agents *in vivo*. **A,** Schematic of an SCLC mouse model with conditional deletion of *Rb1*, *Rbl2,* and *Trp53* (TKO) and generation of conditional *Smyd3* mutant in the TKO background (*TKO;Smyd3*). **B,** Immunoblot analysis of endogenous RNF113A K20me3 methylation following immunoprecipitation of total RNF113A in cell lines originating from TKO and *TKO;Smyd3*-mutant mice. SMYD3 is provided as a validation of successful *Smyd3* deletion in *TKO;Smyd3* mice. Tubulin was used as a loading control. **C,** Schematic of treatment procedures to induce SCLC in TKO and *TKO;Smyd3*-mutant mice followed by the evaluation of therapeutic response to CP. Tumor volume was evaluated by μCT. Animals were enrolled in the study once tumor volume reached approximately 40 mm^3^ for TKO control animals on average at 28 and *TKO;Smyd3* at 35 weeks after tumor induction. Mice cohorts were analyzed at 15 days after enrollment after receiving two rounds of CP or were continuously treated with CP or vehicle (control) until signs of morbidity to establish overall survival. **D,** Representative μCT scans at 15 days after enrollment in TKO and *TKO;Smyd3*-mutant mice treated with vehicle (control) or CP (representative of *n* = 6 mice for each experimental group). Scale bars, 1 cm. **E,** Quantification of tumor volume in TKO and *TKO;Smyd3*-mutant mice treated with vehicle (control) or CP. Boxes represent 25th to 75th percentiles; whiskers: min. to max.; center line: median. *P* values were calculated by two-way ANOVA with the Tukey test for multiple comparisons. **F,** Representative hematoxylin and eosin (H&E) and IHC staining for cell proliferation marker phospho-Histone 3 (pH3) and apoptosis maker cleaved caspase-3 (cl. caspase-3) of lung tissue from vehicle (control) and CP-treated TKO and *TKO;Smyd3*-mutant mice (representative of *n* = 6 mice for each experimental group). Scale bars, 50 μm. **G** and **H,** Quantification of proliferation (pH3-positive cells; **G**) and apoptosis (cl. caspase-3–positive cells; **H**) in samples as in **F**. Boxes represent 25th to 75th percentiles; whiskers: min. to max.; center line: median. *P* values were calculated by two-way ANOVA with the Tukey test for multiple comparisons. **I,** Kaplan–Meier survival curves of control *TKO* (med. survival post enrollment: 21 days, *n* = 8), control *TKO;Smyd3* (med. survival post enrollment: 24 days, *n* = 8), TKO + CP treatment (med. survival post enrollment: 35.5 days, *n* = 8) and *TKO;Smyd3* + CP treatment (med. survival post enrollment: 71 days, *n* = 9)*. P* values were calculated by the log-rank test. **J,** Schedule protocol for SCLC PDX treatment with CP and SMYD3 inhibitor EPZ031686 (SMYD3i). Mice undergoing monotherapy also received vehicle treatment. **K** and **L,** Tumor volume quantification for patient-derived SCLC xenografts obtained from therapy-naïve (**K**) and treated with standard chemotherapy (carboplatin and etoposide) patient (**L**) grafted subcutaneously to immunocompromised NSG mice (*n* = 6 mice, for each treatment group). *P* values were calculated by two-way ANOVA with the Tukey test for multiple comparisons. Data are represented as mean ± SEM. In all panels, representative of at least three independent experiments is shown unless stated otherwise. The numbers below the immunoblot lines represent the relative signal quantification (see also Supplementary Table S5).

We aimed to validate the efficacy of combining SMYD3i with alkylating chemotherapy in preclinical models of SCLC. To that end, we utilized a mouse model of SCLC driven by the conditional loss of genes commonly inactivated in human SCLC, specifically *Rb1*^LoxP/LoxP^, *Rbl2*^LoxP/LoxP^, and *Trp53*^LoxP/LoxP^ (referred to as triple knockout or TKO; Supplementary Fig. S7A; ref. 39). Tumorigenesis in TKO-mutant mice was induced by intratracheal administration of adenovirus expressing Cre-recombinase (Ad-Cre) at 8 weeks of age. As expected, 4 to 6 months after Ad-Cre induction, the control TKO mice developed morbid disease with large metastatic tumors that closely resembled human SCLC. Consistent with our observations in human SCLC, we noted significant elevation of SMYD3 expression in tumors from the TKO model—as well as in tumor samples from a second SCLC mouse model [ref. 40; *Rb*^LoxP/LoxP^;*Trp53*^LoxP/LoxP^;*H11*^LSL-MycT58A^ (RPM)]—compared with normal lung tissue (Supplementary Fig. S7B and S7C). To directly explore a role for SMYD3 in SCLC responses to chemotherapy, we generated conditional *Smyd3*^LoxP/LoxP^-mutant mice (18) crossed with the TKO cancer model (*TKO;Smyd3*; Fig. 7A; Supplementary Fig. S7D and S7E). Importantly, we confirmed that RNF113A is trimethylated at K20 in TKO but not in *TKO;Smyd3* tumors (Fig. 7B). Tumor burden in TKO and *TKO;Smyd3*-mutant mice was evaluated using microcomputed tomography (μCT), and when tumors reached a volume of approximately 40 mm^3^, animals were treated with CP. Animals were analyzed 15 days after enrollment to the treatment study (Fig. 7C–H). As expected, we observed that placebo-treated TKO and *TKO;Smyd3* animals showed rapid tumor growth and development of morbidity. CP treatment attenuated tumor growth in TKO control mice leading to the stabilization of tumor burden after 15 days of treatment, but eventually showed signs of progressive disease. In contrast, CP treatment of *TKO;Smyd3-*mutant mice triggered regression of disease with significantly reduced tumor volume (Fig. 7D and E), decrease in cell proliferation (Fig. 7G), and increase in cell apoptosis (Fig. 7H). Consistent with these observations, *TKO;Smyd3*-mutant mice treated with CP had significantly increased life span (Fig. 7I; median survival of 71 days after enrollment) relative to TKO control group treated with CP or placebo (Fig. 7I; median survival of 35.5 and 21 days, respectively). This observation of nearly 3-fold improvement of survival in CP-treated SMYD3-depleted SCLC mice over placebo-treated controls is particularly remarkable as our experimental design mimics the terminal stage of the disease. Additionally, the ablation of SMYD3 alone had only minimal effect on the life span of placebo-treated mice (Fig. 7I; median survival of 21 days for TKO vs. 24 days for *TKO;Smyd3* mice), suggesting a specific synergistic effect between CP and SMYD3 repression. Notably, analyses of tumor biopsy lysates showed that SMYD3 expression is higher in TKO-mutant mice treated with CP compared with naïve tumor samples (Supplementary Fig. S7F), suggesting that increased SMYD3 expression correlates with prolonged exposure to CP.

Finally, two independent patient-derived xenografts (PDX) samples were obtained from therapy-naïve (PDX#1) and previously treated with standard chemotherapy (carboplatin and etoposide; PDX#2) patients. Both PDX samples were grafted into immunocompromised *NOD.SCID-IL2Rg*^−/−^ (NSG) mice and monitored for tumor growth. Four different treatments were initiated when tumors reached ∼200 mm^3^ in size: (i) vehicle control, (ii) SMYD3 inhibitor EPZ031686, (iii) CP, and (iv) combination therapy of EPZ031686 + CP (Fig. 7J). Upon treatment, the SMYD3i modestly attenuated tumor growth compared with vehicle control in both chemo-naïve and previously treated PDX tumors (Fig. 7K and L). CP was partially effective in both the chemo-naïve and the previously treated PDX, and tumors started to regrow upon continued treatment, indicating the emergence of drug resistance (Fig. 7K and L). In contrast, combined SMYD3 inhibition and CP therapy significantly restrained tumor progression for the full duration of the treatment protocol, well after other treatment conditions had failed (Fig. 7K and L). Histopathologic analyses confirmed that the SMYD3 inhibitor and CP combination resulted in less proliferation and more apoptotic cells, without an observable effect on overall mouse weight, suggesting minimal toxicity of the combination therapy (Supplementary Fig. S8A–S8H). Taken together, combining a clinical-grade SMYD3 inhibitor with an alkylating chemotherapy is well tolerated and highly effective in SCLC.

## DISCUSSION

In a previous work, we characterized the first clearly defined mechanism of SMYD3 oncogenic activity in KRAS-induced lung and pancreatic ductal adenocarcinoma (18). In this context, overexpression of SMYD3 actively participates in cancer progression by synergizing with the RAS–ERK oncogenic pathway through MAP3K2 methylation. This methylation event impairs MAP3K2 kinase inactivation by blocking its interaction with the phosphatase complex PP2a, leading to a constitutively activated form of MAP3K2 and aberrant overstimulation of the downstream MAPK pathway. However, because SMYD3 is also overexpressed in various RAS-independent cancers, the lysine methyltransferase SMYD3 likely operates through other oncogenic mechanisms in different types of cancer.

In the present study, we find that SMYD3 is overexpressed in SCLC, a cancer type not associated with the alteration of the RAS pathway. We demonstrate that genetic or pharmacologic inhibition of SMYD3 significantly increases SCLC sensitivity to alkylation chemotherapy. We found that this effect is mediated by a novel mechanism in which SMYD3 methylation of RNF113A directly blocks binding of the multi-subunit PP4 phosphatase complex. We show that the E3 ubiquitin ligase activity of RNF113A is dependent on its phosphorylation level and that methylation by SMYD3 results in constitutive activation of RNF113A. RNF113A has been linked to alkylation damage repair through the ASCC repair complex. The recruitment of the ASCC3 helicase and the repair enzyme ALKBH3 is facilitated by the ASCC2 subunit, which recognizes RNF113A-mediated ubiquitination events in nuclear speckle bodies (19). Interestingly, RNF113A and this repair machinery appear to be selectively activated by alkylation damage. Thus, targeting this pathway may be of broad clinical use in tumors where such agents are utilized.

The use of alkylating antineoplastic agents remains one of the established treatments for various cancers. These alkylation therapies are based on the capacity to alkylate DNA and efficiently kill highly proliferative cancer cells by promoting nucleic acid damage (8). Unfortunately, their efficacy is limited because of toxicity and acquired resistance. Alkylating agents in combination with other drugs had been commonly used to treat SCLC until a less toxic option became available with the discovery of platinum-based therapies (41). However, there is no clear evidence of better efficacy, and several studies suggest that alkylation chemotherapy, and especially CP, can still be of use with optimized protocols or in combination with other therapies (3–5). Notably, current therapeutic options for SCLC have not evolved for decades and remain poorly effective and prone to resistance, leading to a less than 7% survival after 5 years (7, 9). Therefore, improving sensitivity and limiting acquired resistance to alkylating agents may prove to be highly beneficial for certain patients.

Such acquired resistance may originate from the abnormal regulation of RNF113A and the ASCC repair response. Because SMYD3 overexpression is frequent in cancer and its genetic depletion has no developmental consequences identified to date (ref. 19; International Knockout Mouse Consortium), an interesting possibility to optimize alkylation-based therapies would be to combine them with SMYD3 pharmacologic inhibition. Indeed, we show here that this combination has a dramatic effect, using both an SCLC mouse model and PDX tumor models. Notably, our data indicate a synergistic effect of these two agents *in vitro*. It would be interesting to determine if SMYD3 inhibition can allow for a dose reduction of alkylating agents to decrease toxicity for patients without affecting chemotherapy efficacy. Intriguingly, RNF113A has been recently linked to cisplatin resistance in lung adenocarcinoma (42). However, cisplatin is generally mislabeled as an alkylating agent, and we found that cisplatin does not directly activate RNF113A E3 ligase activity in SCLC and that SMYD3–RNF113A signaling does not affect SCLC cell sensitivity to cisplatin. Regardless of the mechanisms involved, RNF113A involvement in both cisplatin in lung adenocarcinoma and alkylation-based therapy resistance in SCLC is particularly attractive, as both agents are frequently used together in certain cancer combination treatments. For example, RNF113A inhibition could be highly beneficial in the context of therapy using the PCDE combination regimen (cisplatin, cyclophosphamide, doxorubicin, and etoposide; ref. 4). Furthermore, SMYD3 has been previously linked to cancer resistance to chemotherapy (43), and we previously showed that SMYD3 inhibition can potentiate the efficacy of a MEK inhibitor in the context of RAS-induced lung adenocarcinoma (18). Thus, both SMYD3 and RNF113A seem to be key proteins for tumor sensitivity and acquired resistance to various chemotherapeutic drugs.

Our present study focuses on the implication of SMYD3–RNF113A signaling in SCLC resistance to alkylation-based chemotherapy. Interestingly, we found that both SMYD3 and RNF113A are similarly expressed between the four recently characterized SCLC subtypes. Therefore, targeting this pathway may be applicable in all SCLC subtypes, and it is likely that the depicted pathway can participate in other tumor contexts where SMYD3 overexpression is observed. Due to the multiple escape pathways that cancers develop to resist antitumor treatments, a combination of cytotoxic chemotherapies with one or several targeted therapies is often required. The new mechanism identified here provides a rationale for the therapeutic use of SMYD3 inhibitors to mitigate the efficacy of alkylation chemotherapy in first- or second-line treatments for patients with SCLC.

## METHODS

### Ethics

Mice used in this study were housed in an American Association for Laboratory Animal Care (AALAC)–accredited animal facility at The University of Texas MD Anderson Cancer Center (MDACC). Mouse handling and care followed the NIH Guide for Care and Use of Laboratory Animals. All animal procedures followed the guidelines of and were approved by the MDACC Institutional Animal Care and Use Committee [IACUC protocol 00001636, principal investigator (PI): P.K. Mazur]. All tumor specimens were collected after written informed consent was obtained from the patients and in accordance with the institutional review board–approved protocols of the MDACC (PA19-0435, PI: P.K. Mazur). PDXs were obtained from the NCI Patient-Derived Models Repository (PDMR), NCI-Frederick, Frederick National Laboratory for Cancer Research (Specimen ID: 638129-119-R, 541946-237-B).

### Bioinformatics Analysis

The lung cancer transcriptomic data were obtained using Affymetrix Human Genome U133 Plus 2.0 Arrays, normalized and log_2_ transformed. The mean expression value of each lung cancer subtype was compared with the mean value of the normal lung tissue samples. Raw and normalized data are available on the Gene Expression Omnibus (GSE30219). For the SCLC subtype analysis, transcriptomic data were obtained from available RNA-seq data (NIHMS782739-Suppl_Table10; ref. 30) and classification in the NAPY 4 subtypes (NEUROD1^+^, ASCL1^+^, POU3F2^+^, and YAP1^+^) performed according to previous analysis (NIHMS1023395-Supplementary_Table_1; 29).

### Cell Culture, Transfections, Drug Screen, and Cell Viability Assays

HeLa (RRID:CVCL_0030), HeLa S3 (RRID:CVCL_0058), and 293T (RRID:CVCL_0063) cells were grown in DMEM (GIBCO) supplemented with 10% FBS (Dutcher) and 100 U/mL penicillin/streptomycin. DMS-114 (RRID:CVCL_1174), H1048 (RRID:CVCL_145), H1092 (RRID:CVCL_1454), H209 (RRID:CVCL_1525), H69 (RRID:CVCL_1579), H2171 (RRID:CVCL_1536), H82 (RRID:CVCL_1591), H211 (RRID:CVCL_1529), and H196 (RRID:CVCL_1509) cells were cultured in RPMI medium (Gibco) supplemented with 10% FBS (Dutscher), 100 U/mL penicillin/streptomycin. All cells were cultured at 37°C in a humidified incubator with 5% CO_2_. HeLa S3 cells were cultured under gentle agitation using a rotating platform. All cell lines were regularly checked for *Mycoplasma* contamination using the MycoAlert Mycoplasma Detection Kit (Lonza).

For transient expression, cells were transfected with the Mirus 293T transfection reagent and collected 36 hours after transfection. For cell transduction experiments, virus particles were produced by cotransfection of 293T cells with retroviral pMSCV (HA/FLAG-tagged shRNA-resistant RNF113A WT, K20F, S6A, N4 (S43/45/46/47A), N5 (S6/43/45/46/47A), ΔRING, pLentiCMV (SMYD3), and packaging pVSVg, pΔ8.2, and pUMCV plasmids. Viruses were then collected and filtrated and used for the infection of relevant cells, followed by 5 μg/mL blasticidin or 400 μg/mL neomycin selection for one week. For constitutive or inducible knockdown experiments, virus particles were produced by the cotransfection of 293T cells with pSicoR or pLKO-tetON vectors containing specific shRNA target sequences (18, 19), using the packaging plasmids pVSVg and pΔ8.2. After 48 hours of transfection, the supernatant containing virus was collected and filtrated and used to transduce target cells. Infected cells were selected 24 hours after media replacement with 2 μg/mL puromycin or 5 μg/mL blasticidin or 400 μg/mL neomycin for one week.

Cell drug screening was performed as previously described (44). In brief, H209 cells were seeded at 8 × 10^3^ cells/mL in 96-well plates. Cells were then subjected to treatment with cisplatin (1 μmol/L, Selleckchem) or preactivated form of cyclophosphamide 4-hydroperoxy-cyclophosphamide (4H-CP; 2.5 μmol/L final concentration, Cayman Chemicals) and drug library (1 μmol/L; see Supplementary Table S1) or DMSO (vehicle control). The viability of treated cells was measured using Alamar Blue (Invitrogen) after 120 hours.

To test cell survival upon treatment with DNA-damaging agents, cells were cultured overnight in a 96-well plate in 100 μL media. Cells were then treated with indicated concentration of MMS (Sigma-Aldrich), 4H-CP (Niomech), or cisplatin (Euromedex) for 24 hours at 37°C. Treatment media were then replaced with standard growth media, and cell viability was assessed 72 hours later using the PrestoBlue assay (Thermo Scientific).

### Animal Models

*Rb1* ^LoxP/LoxP^, *Rbl1*^LoxP/LoxP^, *Trp53*^LoxP/LoxP^, *H11*^LSL-MycT58A^, and *Erk5*^LoxP/LoxP^ have been described previously (39, 45–47). Reporter-tagged insertion with conditional potential *Smyd3*^tm1a(EUCOMM)^ mouse strain was obtained from the European Mouse Mutant Archive repository (48) and has been characterized previously (18). Briefly, the *Smyd3*^tm1a(EUCOMM)^ targeted knockin sequence includes the Neo-LacZ cassette flanked by *Frt* sites and exon 2 sequence flanked by *LoxP* sites. Founder mice (*Smdy3*^LacZ^) were confirmed as germline-transmitted via cross-breeding with C57BL/6N WT animals. Next, *Smyd3*^LacZ^ mice were crossed with *Rosa26*^FlpO^ deleter strain (49) to generate conditional allele *Smyd3*^LoxP/LoxP^. The *Rosa26-LSL-Mek5*^S311D/T315D^ model was generated by knockin of the CAG-LoxP-Stop-LoxP-V5-Mek5^S311D/T315D^ cDNA-polyA cassette into intron 1 of *Rosa26* using methods previously described (50). Founder animals were identified by PCR followed by sequence analysis, and germline transmission was confirmed by cross-breeding with C57BL/6N WT animals. All mice were maintained in a mixed C57BL/6;129/Sv background, and we systematically used littermates as controls in all the experiments. Immunocompromised NSG mice were used for tumor xenograft studies. All experiments were performed on balanced cohorts of male and female mice as our data did not indicate significant differences in disease progression or response to treatment between females and males. All animals were numbered and experiments were conducted in a blinded fashion. After data collection, genotypes were revealed and animals were assigned to groups for analysis. For treatment experiments, mice were randomized. None of the mice with the appropriate genotype were excluded from this study or used in any other experiments. Mice had not undergone prior treatment or procedures. All mice were cohoused with littermates (2–5 per cage) in a pathogen-free facility with a standard controlled temperature of 72°F, with humidity of 30% to 70%, and a light cycle of 12 hours on/12 hours off set from 7 a.m. to 7 p.m. and with unrestricted access to standard food and water under the supervision of veterinarians, in an AALAC-accredited animal facility at the MDACC. Mouse handling and care followed the NIH Guide for Care and Use of Laboratory Animals. All animal procedures followed the guidelines of and were approved by the MDACC IACUC (protocol 00001636, PI: P.K. Mazur).

### SCLC Mouse Models

To generate tumors in the lungs of *Rb1*^LoxP/LoxP^, *Rbl2*^LoxP/LoxP^; *Trp53*^LoxP/LoxP^ (*TKO*); *Rb1*^LoxP/LoxP^, *Rbl2*^LoxP/LoxP^; *Trp53*^LoxP/LoxP^; *Smyd3*^LoxP/LoxP^ (*TKO;Smyd3*); *Rb1*^LoxP/LoxP^, *Rbl2*^LoxP/LoxP^; *Trp53*^LoxP/LoxP^; *Erk5*^LoxP/LoxP^ (*TKO; Erk5*), *Rb1*^LoxP/LoxP^, *Rbl2*^LoxP/LoxP^; *Trp53*^LoxP/LoxP^; *Rosa26-LSL-Mek5*^S311D/T315D^ (*TKO;Mek5*^DD^), and *Rb*^LoxP/LoxP^;*Trp53*^LoxP/LoxP^;*H11*^LSL-MycT58A^ (RPM) mutant mice, we used replication-deficient adenoviruses expressing Cre-recombinase (Ad-Cre) as previously described (39). Briefly, 8-week-old mice were anesthetized by the continuous gaseous infusion of 2% isoflurane for at least 10 minutes using a veterinary anesthesia system (D19 Vaporizer, Vetland Medical). Ad-Cre was delivered to the lungs by intratracheal instillation. Prior to administration, Ad-Cre was precipitated with calcium phosphate to improve the delivery of Cre by increasing the efficiency of viral infection of the lung epithelium. Mice were treated with one dose of 5 × 10^6^ PFU of Ad-Cre (Baylor College of Medicine, Viral Vector Production Core). Mice were analyzed for tumor formation and progression at indicated times after infection.

Tumor size was measured using a digital caliper, and tumor volume was calculated using the formula: volume = (width)^2^ × length/2 where length represents the largest tumor diameter and width represents the perpendicular tumor diameter. The endpoint was defined as the time at which a progressively growing tumor reached 15 mm in its longest dimension, as approved by the MDACC IACUC protocol (00001636, PI: P.K. Mazur), and in no experiments was this limit exceeded.

For the CP treatment experiment, mice were monitored by μCT, as described below. When tumor volumes had reached approximately 40 mm^3^, mice were enrolled to treatment with CP [40 mg/kg once per week, intraperitoneally (i.p.)] in vehicle 0.9% saline. Control animals underwent the same procedure but received vehicle treatment. Two weeks after enrollment, the cohort of mice was sacrificed and tumors were analyzed. The second cohort of mice continued CP treatment until the development of morbid disease. Tumor biopsies were collected, and protein lysates were prepared to confirm the mutation of conditional alleles by immunoblotting.

### Microcomputed Tomography

μCT scans were performed on TKO and *TKO;Smyd3* tumor-bearing mice at approximately 28 weeks after Ad-Cre induction as previously described (51). In brief, mice were anesthetized by a continuous gaseous infusion of 2% isoflurane for at least 10 minutes using a veterinary anesthesia system. The mice were intubated using a 20 gauge × 1 inch catheter and were transferred onto the bed of an Explore Locus RS preclinical *in vivo* scanner (GE Medical Systems). The mice were mechanically ventilated in a small animal ventilator, and μCT images were captured at 80 kV and 450 microamperes. The X-ray source and CCD-based detector gantry were rotated around the subject in roughly 1.0-degree increments. The raw data were reconstructed to a final image volume of 875 × 875 × 465 slices at 93-μm^3^ voxel dimensions. The total chest space volume, including the heart, was selected using manual segmentation. An optimal threshold value was automatically determined using the function of the MicroView analysis software. Tumors formed in the lung can be distinguished from other soft tissue in a reconstructed 3-D image of the higher voxels; therefore, the tumor nodule structure was selected using a combination of manual segmentation and semiautomated contouring of the optimal threshold value. These analyses were consistent between two independent operators and were performed by a well-trained researcher in a blinded manner.

### Xenograft Models

PDXs were obtained from the NCI PDMR, NCI-Frederick, Frederick National Laboratory for Cancer Research (Specimen ID: 638129-119-R, 541946-237-B). Briefly, surgically resected tumor specimens were obtained from deidentified patients with histologically confirmed SCLC. PDX#1 (638129-119-R) was derived from patients who have not received any chemotherapy prior to biopsy. PDX#2 (541946-237-B) was derived from patients who received carboplatin and etoposide therapy for 3 months with partial response followed by disease progression. All tumor specimens were collected after written patient consent and in accordance with the institutional review board–approved protocols of the MDACC (PA19-0435, PI: P.K. Mazur). PDX tumors were generated and propagated by transplanting small tumor fragments isolated directly from surgical specimens subcutaneously into NSG mice as we established previously (44). Whole-exome sequencing was performed and cancer gene panel analysis revealed that PDXs are carrying mutations characteristic for SCLC, specifically PDX#1: *RB1*^p.X473_splice^; *TP53*^pX224_splice^; *CREBBP*^p.E371Rfs*56^; *MSH3*^p.A61Pfs*25^ and PDX#2: *RB1*^p.X738_splice^; *TP53*^pR249G^; *KMT2D*^p.A1390Qfs*27^; *MSH3*^p.V1192Cfs*2^. When tumors became palpable, they were calipered to monitor growth kinetics. For therapy studies, mice were treated as indicated with CP (40 mg/kg once per week, i.p.) in vehicle 0.9% saline and EPZ031686 (SMYD3i, 25 mg/kg daily, i.p.) in vehicle 10% (2-hydroxypropyl)-β-cyclodextrin. Control and monotherapy animals underwent the same procedure but received vehicle treatment.

For xenograft studies, the human SCLC cell line NCI-H1092 was transduced with lentivirus expressing sgRNA/Cas9 targeting SMYD3 or MAP3K2 and selected with puromycin. The cells were trypsinized and singularized. The trypsin was washed with an excess growth medium, and the cells were counted. The cells were then resuspended in PBS and mixed with matrigel (1:1) at a density of 2 × 10^7^ cells per mL and kept on ice until injection. Next, 100 μL of the cell suspension was injected subcutaneously into the hind flanks of NSG mice. When tumors became palpable, they were calipered to monitor growth kinetics. For therapy studies, mice were treated as indicated with CP (40 mg/kg once per week, i.p.) in vehicle 0.9% saline. Control animals received vehicle treatment.

### Histology and IHC

Tissue specimens were fixed in 4% buffered formalin for 24 hours and stored in 70% ethanol until paraffin embedding. Sections (3 μm) were stained with hematoxylin and eosin or used for IHC studies. Human tissue sections were collected in accordance with the institutional review board–approved protocols of the MDACC (PA19-0435, PI: P.K. Mazur), and written informed consent was obtained from the patients. IHC was performed on formalin-fixed, paraffin-embedded mouse and human tissue sections using a biotin–avidin method as described previously (18). The following antibodies were used (at the indicated dilutions): cleaved caspase-3 (RRID:AB_2070042, Cell Sig­naling Technology, 1:100), phosphor-Histone 3 (RRID:AB_331535, Cell Signaling Technology, 1:1,000), SMYD3 (RRID:AB_2682458, Sigma-Aldrich 1:300). Sections were developed with DAB and counterstained with hematoxylin. Pictures were taken using a PreciPoint M8 microscope equipped with PointView software. Analysis of the tumor area and IHC analysis were done using ImageJ software. IHC was quantified using the H-score metric that ranges from 0 to 300 and integrates IHC staining intensity and area, performed as previously described (35).

### Methylation Assay

ProtoArray version 5.0 (Invitrogen) was incubated overnight with either recombinant GST-control or GST-SMYD3 and the tritium radiolabeled cofactor ^3^H-SAM, as detailed in ref. 31. Methylation was then revealed by autoradiography. *In vitro* methylation assays were completed using 1 to 2 mg of recombinant proteins or peptides, which were incubated with 1 mg of recombinant SMYD3 and 0.1 mmol/L S-adenosyl-methionine (SAM; Sigma-Aldrich) or 0.1 mmol/L S-adenosyl-l-methionine-d3 tetra (p-toluenesulfonate) salt (deuterated SAM, CDN isotope) or 2 μCi SAM[^3^H] (IsoBio) in buffer containing 250 mmol/L Tris-HCl (pH 8.0), 50% glycerol, 100 mmol/L KCl, 25 mmol/L MgCl_2_ at 30°C overnight. The reaction was analyzed by SDS-PAGE, followed by autoradiography, Coomassie stain, or mass spectrometry analyses.

### Mass Spectrometry–Based Proteomic Analysis to Identify Methylation Sites

For LC/MS-MS analysis of recombinant RNF113A methylation, deuterated SAM was used to rule out possible artifactual chemical methylation *in vitro*, shifting the mass of one methyl group from 14.016 to 17.034 Da. After SDS-PAGE separation and Coomassie (GelCode Blue, Thermo Scientific), recombinant RNF113A was sliced from gels and digested with trypsin (Promega). Resulting peptides were analyzed by online nano LC/MS-MS (UltiMate 3000 RSLCnano and Q-Exactive Plus, Thermo Scientific). To that end, peptides were sampled on a 300 μm × 5 mm PepMap C18 precolumn (Thermo Scientific) and separated on a 75 μm × 250 mm C18 columns (Reprosil-Pur 120 C18-AQ, 1.9 μm, Dr. Maisch). MS and MS-MS data were acquired using Xcalibur (Thermo Scientific). Mascot Distiller (Matrix Science) was used to produce mgf files before the identification of peptides and proteins using Mascot (version 2.7; RRID:SCR_014322) through concomitant searches against in-house databases containing the sequences of proteins of interest, standard contaminants database, and the corresponding reversed databases. The Proline software (52) was used to filter the results with the following settings: conservation of rank 1 peptides, peptide length ≥6 amino acids, identity threshold of peptide-spectrum-match <0.01, minimum peptide-spectrum-match score of 25, and minimum of 1 specific peptide per identified protein group. Peptides of interest were subsequently targeted by LC-Parallel Reaction Monitoring using an UltiMate 3000 RSLCnano coupled to a Q-Exactive HF (Thermo Scientific). Candidate methylation sites were verified by manual inspection.

### Expression and Purification of Recombinant Proteins

Recombinant proteins were purified from *Escherichia coli* BL21 bacteria cells transformed with vectors (pGex6.1) expressing respective cDNA. Cells were resuspended in lysis buffer containing 50 mmol/L Tris pH 7.5, 150 mmol/L NaCl, 0.05% NP-40, 0.25 mg/mL lysozyme, 0.5 mmol/L PMSF and protease inhibitors, and additionally sonicated. GST-tagged proteins were purified using Glutathione Sepharose 4B beads (GE Healthcare) and eluted with 10 mmol/L reduced L-glutathione (Sigma-Aldrich) or cleaved from the beads using purified PreScission protease.

### Peptide Pulldown, Dimethyl Labeling, and Mass Spectrometry Analysis of Methyl-Sensitive Binders

To perform peptide pulldown, 10 μL of streptavidin Sepharose beads (GE Healthcare) were saturated with 7.5 μg of specific biotinylated peptides in peptide buffer (50 mmol/L Tris pH 8, 150 mmol/L NaCl, 0.5% NP40, 0.5 mmol/L DTT, 10% glycerol, complete protease inhibitors; Roche) for 2 hours at 4°C with rotation. Next, beads were washed in the peptide buffer and incubated with either 2 μg of recombinant proteins or 1 mg of whole-cell extract in peptide buffer for 4 hours at 4°C with rotation. Beads were then washed 3 times in peptide buffer. For direct identification, beads were then eluted in Laemmli buffer and analyzed by immunoblotting. For mass spectrometry analyses, proteins still bound to beads were denatured and disulfide bonds reduced in digestion buffer (2 M urea, 10 mmol/L DTT, and 100 mmol/L Tris pH 8), after which cysteines were alkylated using 50 mmol/L iodoacetamide. Then proteins were digested overnight using trypsin. Each digested sample (i.e., RNF113A me0 and RNF113A me3 peptides interactors) was loaded on StageTip for purification, followed by differential dimethyl labeling with either light (CH_2_O) or heavy (CD_2_O) label. Each pair of corresponding peptide pulldowns (forward = RNF113A me0/CH20 vs. RNF113A me3/CD20; reverse = RNF113A me3/CD20 vs. RNF113A me0/CH20) were then pooled and analyzed using reverse-phase Easy-nLC 1,000 coupled online to a Thermo Fisher Orbitrap Fusion Tribrid mass spectrometer using a 140-minute gradient of buffer B (80% acetonitrile, 0.1% TFA). Raw data were analyzed using MaxQuant (ref. 53; RRID:SCR_014485) to quantify the ratio of each potential binder to the K20me0 and K20me3 peptides and further filtered for contaminants and reverse hits using Perseus (54). Proteins identified as outliers in both experiments are assigned as significant interactors and written in either blue (methyl interactors) or red (methyl-repelled interactors).

RNF113A peptides used for pulldown were purchased from Caslo: Biotin—Ahx—DQVCTFLF Kme0 KPGRKG—CONH2, Biotin—Ahx—DQVCTFLF Kme1 KPGRKG—CONH2, Biotin—Ahx—DQVCTFLF Kme2 KPGRKG—CONH2, Biotin—Ahx—DQVCTFLF Kme3 KPGRKG—CONH2, and Biotin—Ahx—DQVCTFLFK Kme3 PGRKG—CONH2.

### Immunoprecipitations and Protein Purification

For GST pulldown, GST Recombinant proteins (PPP4R3a and RNF113A mutants) were purified as described above. GST tag from PPP4R3a was cleaved by PreScission protease and dialyzed. Excess GST was captured with Glutathione Sepharose 4B beads (GE Healthcare) followed by overnight dialysis. GST-tagged RNF113A mutants were bound to Glutathione Sepharose beads for a minimum of 1 hour and afterward washed three times in the wash buffer. Purified PPP4R3a was then added to RNF113A bound to GST beads and incubated overnight at 4°C. After three washes in wash buffer, proteins were eluted in Laemmli buffer. Dialysis and washes were completed in buffer containing 150 mmol/L NaCl, 50 mmol/L Tris pH 8, 0.1% NP40, 0.5 mmol/L EDTA, and 0.5 mmol/L DTT. Direct interaction between proteins was analyzed by immunoblotting using mouse monoclonal anti-GST antibody (RRID:AB_627677, Santa Cruz Biotechnology).

For the coimmunoprecipitation of ectopic proteins, the immunoprecipitation of HA-tagged RNF113A WT, K20A, and K20F was completed after transient expression in 293T cells for 36 hours. The cells were resuspended in lysis buffer (50 mmol/L Tris-HCl pH 8.0, 137 mmol/L NaCl, 1 mmol/L EDTA, 0.5% NP40, 10% glycerol, protease and phosphatase inhibitors, 1 mmol/L PMSF). The cell lysate was cleared by centrifugation and added to previously washed anti-HA resin in the same buffer. After overnight incubation at 4°C with rotation, HA resin with bound proteins was washed three times in the same buffer. Proteins were eluted with Laemmli buffer and analyzed by immunoblotting using anti-HA tag antibody (AB_1549585, Cell Signaling Technology).

Immunoprecipitation under denaturing conditions was performed as previously described (19). Briefly, 293T cells were transfected with a plasmid expressing His-tagged ubiquitin and FLAG-HA-tagged RNF113A. Cells were treated for 1 hour with 1 mmol/L MMS, and then media were replaced with media containing the proteasome inhibitor MG-132. After 4 hours, a fraction of the cells was collected before addition of the lysis buffer to analyze the protein expression level (input). Cells were resuspended in lysis buffer (6 M guanidinium-HCl, 0.1 M Na_2_HPO4/NaH_2_PO4, 10 mmol/L Tris-HCl pH 8.0, 5 mmol/L imidazole, 10 mmol/L β-mercaptoethanol) and sonicated. After centrifugation, cell lysate was added to previously equilibrated Ni_2+_-NTA agarose beads (Qiagen). Cell lysate was incubated with beads overnight at 4°C with rotation. Beads with bound proteins were extensively washed as follows: once in lysis buffer, once in wash buffer (8 M urea, 0.1 M Na_2_HPO4/NaH2PO4, 10 mmol/L Tris-HCl pH 6.8, 5 mmol/L imidazole, 10 mmol/L β-mercaptoethanol), and twice in wash buffer with 0.1% Triton X-100. Proteins were eluted by incubation with elution buffer (0.2 M imidazole, 0.15 M Tris-HCl pH 6.8, 30% glycerol, 0.72 M β-mercaptoethanol, 5% SDS) for 45 minutes at room temperature with agitation. Laemmli buffer was added, and immunoprecipitation was analyzed by immunoblotting.

RNF113A purification for *in vitro* ubiquitination assay: RNF113A WT or variants used in ubiquitination ligase assays were purified from HeLa stably expressing FLAG/HA-tagged RNF113A. Cell pellet was resuspended in FLAG lysis buffer (50 mmol/L Tris-HCl pH 8.0, 137 mmol/L NaCl, 1 mmol/L EDTA, 1% Triton X 100, 10% glycerol, 0.1 mmol/L PMSF, protease and phosphatase inhibitors) and lysed for 30 minutes at 4°C. After centrifugation, clear cell lysate was added to Anti-FLAG-M2 affinity gel (Sigma-Aldrich). Binding of the protein was allowed for 4 hours or overnight at 4°C. Afterward, beads were extensively washed with cold wash buffer (50 mmol/L Tris-HCl pH 8.0, 450 mmol/L NaCl, 1 mmol/L EDTA, 1% Triton X 100, 10% glycerol, 0.1 mmol/L PMSF), FLAG lysis buffer and TAP buffer (50 mmol/L Tris pH 7.9, 100 mmol/L KCl, 5 mmol/L MgCl_2_, 0.2 mmol/L EDTA, 0.1% NP-40, 10% glycerol, 0.2 mmol/L PMSF). Proteins were eluted with TAP buffer and 0.5 mg/mL 3× FLAG peptide (Sigma-Aldrich) for 4 hours or overnight.

Purification of PP4 from mammalian cells: Purification of the PP4 catalytic subunit was previously described (55). Briefly, 293T cells were transfected with the vector expressing HA-tagged PPP4c subunit. Transfected cells were lysed in immunoprecipitation buffer (0.5% NP-40, 50 mmol/L HEPES-KOH pH 8.0, 100 mmol/L KCl, 2 mmol/L EDTA, 10% glycerol, 1 mmol/L PMSF, 1 mmol/L DTT, protease and phosphatase inhibitors). After centrifugation, cleared cell lysate was incubated with anti-HA resin for 5 hours at 4°C with rotation. Beads with bound proteins were washed twice in immunoprecipitation buffer and twice in wash buffer (50 mmol/L Tris-HCl pH 7.2, 100 mmol/L NaCl, 0.4 mmol/L EDTA, 1 mmol/L DTT). Phosphatase was eluted by three elutions, each 1 hour at 4°C in wash buffer with 0.5 mg/mL HA peptide. Final elutions were supplemented with glycerol to a final concentration of 50%, snap-frozen, and stored at −80°C.

### Immunoblot Analysis

Proteins were separated by SDS-PAGE, transferred to PVDF membrane, and analyzed by immunoblot. The following antibodies were used: RNF113A (RRID:AB_1079821, Sigma-Aldrich), MAP3K2 (RRID:AB_2798822, CST), β-actin (RRID:AB_2223172, CST), streptavidin (RRID:AB_261531, Sigma-Aldrich), PPP4R3 (RRID:AB_597904, Bethyl), GST (RRID:AB_627677, Santa Cruz Biotechnology), HA (RRID:AB_1549585, CST), ubiquitin (RRID:AB_2180538, CST), His (RRID:AB_2115720, CST), pH2A.X (RRID:AB_2891851, Bethyl); H2A.X (RRID:AB_2891857, Bethyl), tubulin RRID:AB_2288090, Santa Cruz Biotechnology), Ku-80 (RRID:AB_2218736, CST), phospho-CDK substrate motif (K/H)pSP (RRID:AB_2714143, CST), RNF113A K20me3 (generated by Eurogentec, speedPTM protocol), and SMYD3 (developed in house, as previously described; ref. 18).

### E3 Ubiquitin Ligase Assay

Alkylation damage was induced with MMS treatment for 30 minutes or indicated time. *In vitro* assays were completed in reaction buffer 25 mmol/L Tris-HCl pH 7.6, 5 mmol/L MgCl_2_, 100 mmol/L NaCl, 1 mmol/L DTT containing 2 mmol/L ATP and 10 μmol/L ubiquitin in a final volume of 30 μL. Ubiquitin activating enzyme 1 (UBE1; Boston Biochemicals) was used at 0.6 μmol/L and ubiquitin-conjugating enzyme (UBCH5c/Ube2D3; Boston Biochemicals) 0.5–1 μmol/L. Purified HA-FLAG-tagged-RNF113A proteins (see below) were added to each reaction mixture. Samples were incubated at 37°C for 2 hours, and reactions were stopped by the addition of Laemmli buffer and analyzed by Western blot.

### TUBE Pulldown

Alkylation damage was induced with MMS treatment for 4 hours or indicated time. Cells were collected and lysed in TUBE lysis buffer (50 mmol/L Tris-HCl pH 7.5, 1 mmol/L EGTA, 1 mmol/L EDTA, 1% Triton X-100, 0.27 M Sucrose, 0.2 mmol/L PMSF, 100 mmol/L iodoacetamide, protease, and phosphatase inhibitors) for 60 minutes at 4°C. Prior to this step, a fraction of the cell pellet was used to confirm DNA damage by pH2A.X immunoblotting. Whole-cell lysate was then added to TUBE2 beads (Recombinant Human Ubiquitin 1 Tandem UBA Agarose, R&D Systems). Samples were incubated overnight at 4°C. Beads were washed 3 times in high salt TAP buffer (50 mmol/L Tris-HCl pH 7.9, 300 mmol/L KCl, 5 mmol/L MgCl_2_, 0.2 mmol/L EDTA, 0.1% NP-40, 10% glycerol, 2 mmol/L β-mercaptoethanol, 0.2 mmol/L PMSF) and twice in low-salt TAP buffer (50 mmol/L Tris-HCl pH 7.9, 0 mmol/L KCl, 5 mmol/L MgCl_2_, 0.2 mmol/L EDTA, 0.1% NP-40, 10% glycerol, 2 mmol/L β-mercaptoethanol, 0.2 mmol/L PMSF). Proteins were eluted in Laemmli buffer, and TUBE pulldowns were analyzed by western blot.

### *In Vitro* Dephosphorylation Assay

*In vitro* dephosphorylation of RNF113A using FastAP thermosensitive alkaline phosphatase (Thermo Fisher) was completed in reaction mixture containing FastAP reaction buffer, 10 U of FastAP phosphatase on whole-cell lysate or immunoprecipitated proteins. Reaction mixture was incubated for 1 hour at 37°C. Similar protocol was used for λ phosphatase (NEB). For dephosphorylation by PP4 phosphatase, purified PPP4c was first incubated in reaction buffer (50 mmol/L Tris-HCl pH 7.2, 0.1 mmol/L CaCl_2_, 5 mmol/L MnCl_2_, and 0.2 mg/mL BSA) for 10 minutes at 30°C. This reaction was then added to WT RNF113A purified as described above. Phosphatase reactions were incubated at 30°C for 1 hour. Changes in the phosphorylation level were analyzed by immunoblotting.

### Immunofluorescence Microscopy

For MMS-induced foci analysis, U2OS cells were treated with 500 μmol/L MMS in the complete medium at 37°C for 6 hours, washed with cold PBS, and then extracted with PBS containing 0.2% Triton X-100 and protease inhibitor cocktail (Pierce) for 20 minutes. After washing again with cold PBS, cells were fixed with 3.2% paraformaldehyde in PBS. Cells were then washed extensively with IF Wash Buffer (PBS, 0.5% NP-40, and 0.02% NaN_3_), then blocked with IF Blocking Buffer (IF Wash Buffer with 10% FBS) for at least 30 minutes. Primary antibodies were diluted in IF Blocking Buffer overnight at 4°C. After staining with secondary antibodies (conjugated with Alexa Fluor 488 or 594; Millipore), samples were mounted using the Prolong Gold mounting medium (Invitrogen). Epifluorescence microscopy was performed using an Olympus fluorescence microscope (BX-53) with an ApoN 60X/1.49 NA or a UPlanS-Apo 100×/1.4 oil immersion lenses and cellSens Dimension software. Foci were quantified for minimum 100 cells in three biological replicates. For foci intensity quantification, original images were imported into Adobe Photoshop and quantified manually, using at least 50 foci from each sample.

### Neutral Comet Assay

HeLa cells expressing doxycycline-inducible shRNA *RNF113A* were seeded 48 hours before treatment with 0.5 mmol/L MMS for 1 hour. Media were replaced with fresh media and incubated for a further 2 hours when indicated. Cells were then washed once with PBS and trypsinized. Cells were resuspended in ice-cold PBS at a concentration of 1 × 10^5^ cells/mL. Then, a neutral comet assay was performed using the CometAssay (Trevigen) kit, according to the manufacturer's protocol. Briefly, cells were mixed with low melting point agarose at a ratio of 1:10, and then 80 μL of this mixture was spread onto a comet slide and incubated at 4°C for half an hour. Slides were immersed in ice-cold lysis buffer (Trevigen) for 1 hour at 4°C, then in 1× TBE buffer (0.1M TrisBase, 0.1M boric acid, 2.5 mmol/L EDTA) for 30 minutes at 4°C. After lysis, cells were electrophoresed in 1× TBE buffer at 20 V for 30 minutes at 4°C. Slides were rinsed with distilled water and incubated with DNA precipitation solution (1M ammonium acetate in 95% ethanol) for 30 minutes at room temperature. Slides were then incubated with 70% ethanol for 30 minutes and dried overnight at room temperature in the dark. DNA staining was done using 1× SYBR Gold (Thermo Fisher) at room temperature for 30 minutes. After drying the slides, images were acquired with an epifluorescence microscope (Zeiss; 10X; AxioVision control software, RRID:SCR_002677). A minimum of 100 comets were scored for each condition using the OpenComet plugin in ImageJ (RRID:SCR_003070).

### Data Availability

All gene-expression data utilized in the study are publicly available. This study did not generate any unpublished code, software, or algorithm. All utilized codes are publicly available as of the date of publication. Any additional information required to reanalyze the data reported in this paper is available from the lead contact, N. Reynoird, upon request. Unique materials created for this study will be available from the lead contact upon request.

## Supplementary Material

Supplementary Data

Supplementary Data

Supplementary Data

Supplementary Data

Supplementary Data

Supplementary Data

Supplementary Data

Supplementary Data

Supplementary Data

Supplementary Table
