# Supplementary material for: SMYD3 Impedes Small Cell Lung Cancer Sensitivity to Alkylation Damage through RNF113A Methylation–Phosphorylation Cross-talk
Source: Cancer Discov. 2022 Jul 12;12(9):2158–79. doi: 10.1158/2159-8290.CD-21-0205 (PMC9437563; doi:10.1158/2159-8290.CD-21-0205)
Supplement: Supplementary Data [file cd-21-0205_supplementary_data_1_suppsd1.pdf]

Supplementary Figure 1

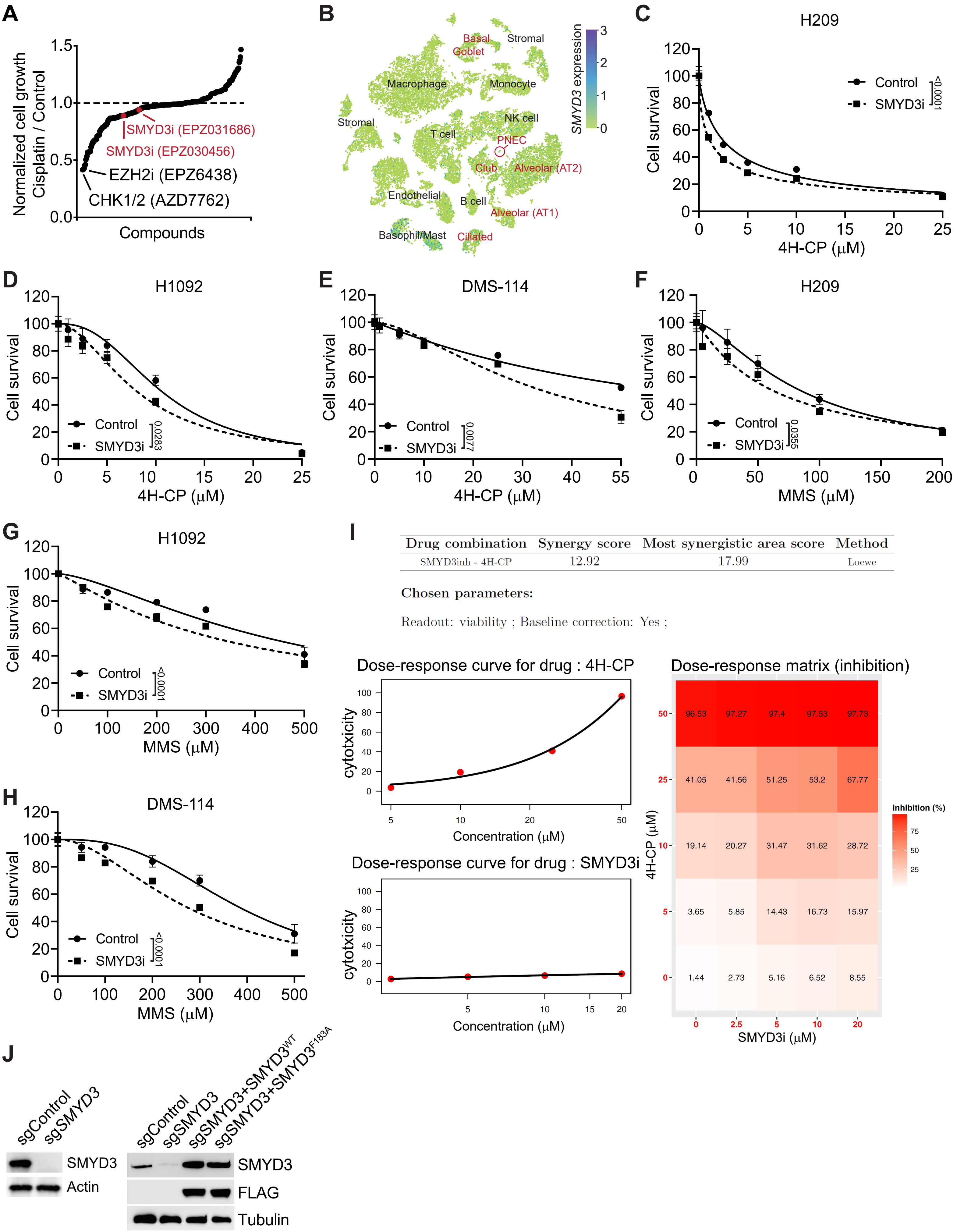

Supplementary Figure S1. SMYD3 is a candidate regulator of SCLC susceptibility to alkylating chemotherapy

A, Synthetic lethality screening using a library comprised of 285 characterized inhibitors, testing H209 SCLC cells sensitivity to cisplatin genotoxicity. Data represent relative growth of H209 cells treated with a combination of cisplatin (1 μM) and different inhibitors (1 μM each) compared to cisplatin alone. **B**, Analysis of normal human lung single-cell RNA sequencing data reveals low *SMYD3* expression in pulmonary neuroendocrine cells (PNEC); clusters of cell types are labeled; lung epithelial cell types in red (Human Lung Cell Atlas (41)). **C-H**, H209, H1092 and DMS-114 SCLC cell viability assays using different concentrations of either 4H-CP (C-E) or MMS (F-H) with or without SMYD3i (EPZ031686). Percentage of viable cells under each condition was normalized to untreated cells. *P-value* were calculated by two-way ANOVA with Tukey's testing for multiple comparisons. Data are represented as non-linear regression with mean ± SEM. **I**, Loewe synergy score calculated by SynergyFinder 2.0, with individual dose-response curves (left) and dose-response matrix (right) for 4H-CP and SMYD3i. **J**, Immunoblot analyses were performed using the indicated antibodies with lysates of H1092 engineered cells used in xenograft assays presented in Figures 1F-H. Actin or Tubulin are shown as a loading control.
