# Supplementary material for: SMYD3 Impedes Small Cell Lung Cancer Sensitivity to Alkylation Damage through RNF113A Methylation–Phosphorylation Cross-talk
Source: Cancer Discov. 2022 Jul 12;12(9):2158–79. doi: 10.1158/2159-8290.CD-21-0205 (PMC9437563; doi:10.1158/2159-8290.CD-21-0205)
Supplement: Supplementary Data [file cd-21-0205_supplementary_data_2_suppsd2.pdf]

Supplementary Figure 2

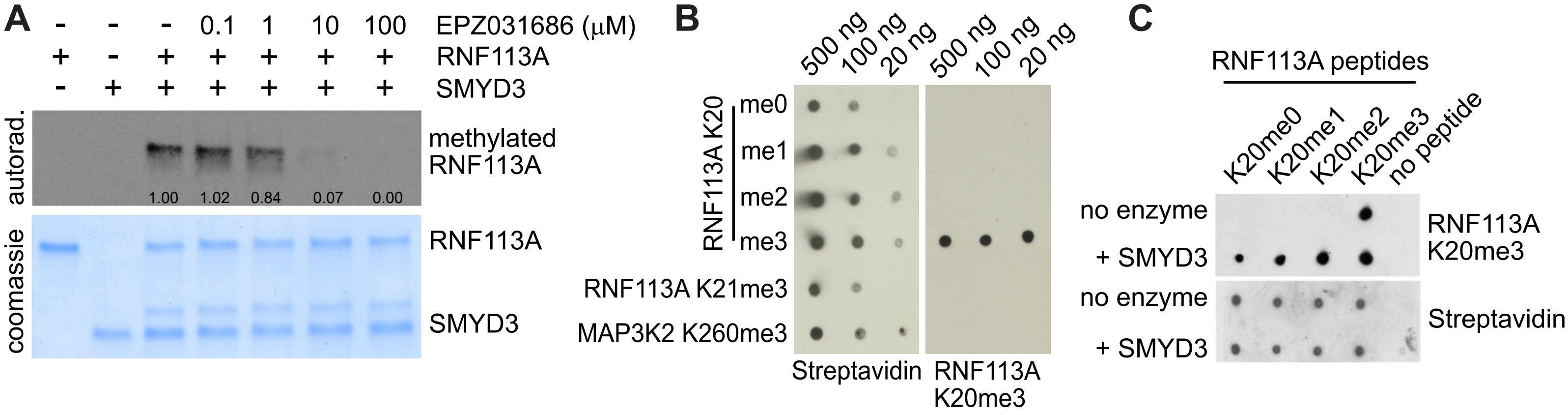

Supplementary Figure S2. Identification of RNF113A as a novel methylated substrate of SMYD3

A. *In vitro* methylation assay were performed using radiolabeled S-adenosylmethionine and recombinant RNF113A and SMYD3, with increasing concentrations of SMYD3 inhibitor (EPZ031686) at the indicated concentrations. Top panel, autoradiogram of methylation assay. Bottom panel, Coomassie stain of proteins in the reaction. **B**, Specific recognition of RNF113A K20me3 peptides by the anti- RNF113A-K20me3 antibody by dot blot analysis using the indicated biotinylated peptides. Streptavidin is shown as the loading control. **C**, Non-radiolabeled *in vitro* methylation assay using recombinant SMYD3 and RNF113A biotinylated peptides with different K20 methylation states as staring material. Methylation events are detected using dot blot and immunodetection by RNF113A K20me3 antibody. Streptavidin is shown as the loading control.

In all panels, representative of at least three independent experiments is shown unless stated otherwise. The numbers below the immunoblot lines represent the relative signal quantification (see also Supplemental Table 5).
