# Supplementary material for: SMYD3 Impedes Small Cell Lung Cancer Sensitivity to Alkylation Damage through RNF113A Methylation–Phosphorylation Cross-talk
Source: Cancer Discov. 2022 Jul 12;12(9):2158–79. doi: 10.1158/2159-8290.CD-21-0205 (PMC9437563; doi:10.1158/2159-8290.CD-21-0205)
Supplement: Supplementary Data [file cd-21-0205_supplementary_data_3_suppsd3.pdf]

Supplementary Figure 3

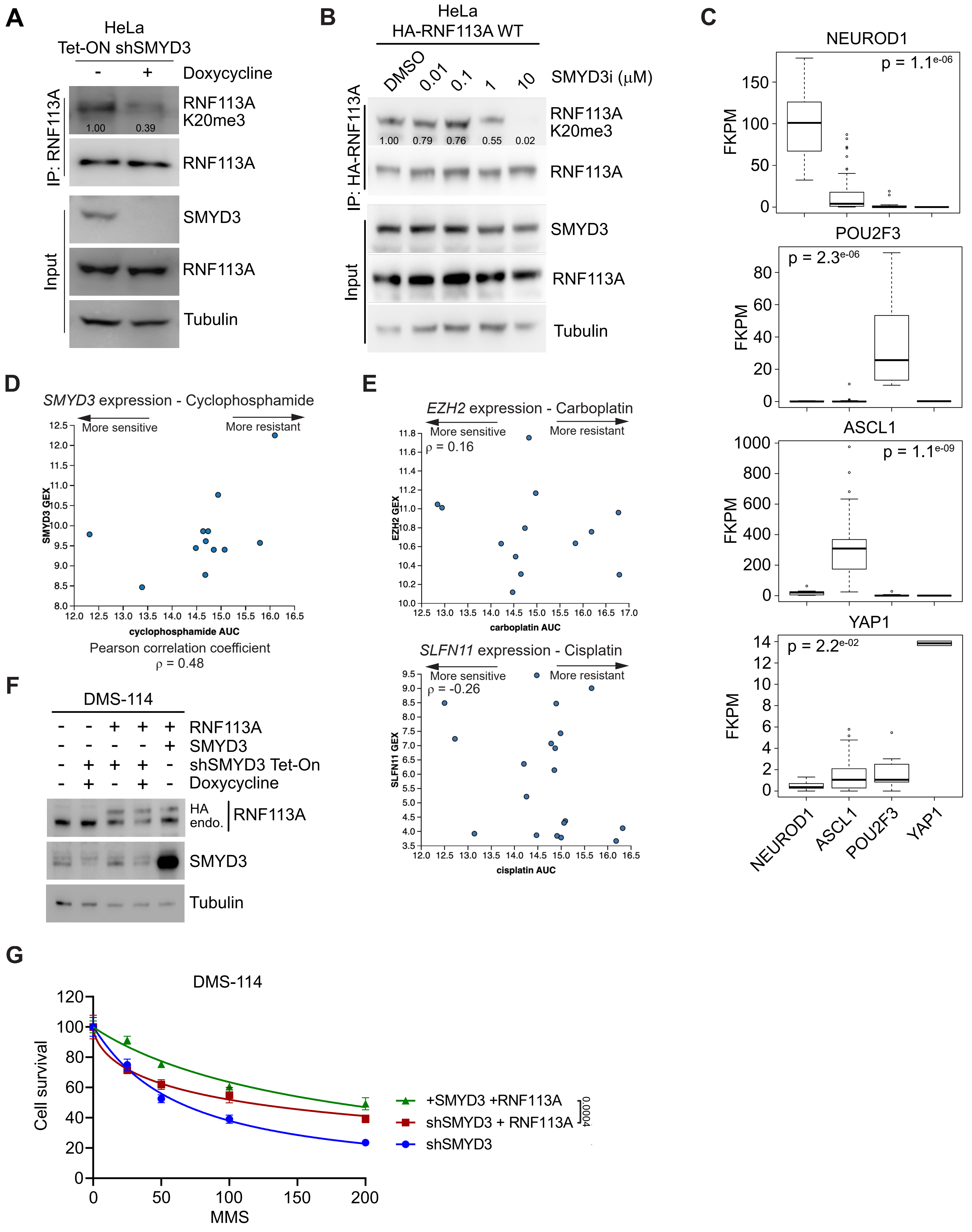

Supplementary Figure S3. Characterization of RNF113A methylation in SCLC cell lines

A, Immunoblot analysis with the indicated antibodies of endogenous RNF113A K20me3 methylation following immunoprecipitation of total RNF113A from HeLa cells expressing doxycycline-inducible shRNA against SMYD3. Tubulin is shown as a loading control. **B**. Immunodetection of RNF113A K20me3 following immunoprecipitation of stably expressed HA-RNF113A in HeLa cells after treatment with different concentrations of SMYD3i. Tubulin is shown as a loading control. **C**, Related to Figure 3D, validation of NAPY markers expressions (NEUROD1; ASCL1; POU2F3; YAP1) in classified SCLC subtypes of human lung cancer. Boxes represent 25th to 75th percentile, whiskers: 10% to 90%, center line: median. *P-value* were calculated by Kruskal-Wallis test. Analysis was performed using FPKM data for each specified gene obtained from NIHMS782739-Suppl_Table10 (34). NAPY SCLC subclassification was based on the original classification by Rudin et al., presented in NIHMS1023395-Supplementary_Table_1 (32). **D-E**, Pearson correlation analyses of SMYD3 expression and SCLC cell lines resistance to cyclophosphamide (D, $\rho = 0.48$) and of EZH2 and SLFN11 expressions and SCLC cell lines resistance to platinum-based therapy (E, $\rho = 0.16$ and $\rho = -0.26$ respectively). The area under curve (AUC) was calculated by integration under the 16-point concentration-response curves, using the Broad Institute and NCI's Cancer Target Discovery and Development Network: Cancer Therapy Response Portal (CTRP). Pearson correlation coefficient (ρ) was calculated between gene expression and AUC. **F-G**, Immunoblot analysis was performed with indicated antibodies using lysates of engineered DMS-114 cells (F) which were then used in cell survival assays using different concentrations of MMS (G). Percentage of living cells under each condition was normalized to untreated cells. *P-value* were calculated by two-way ANOVA with Tukey's testing for multiple comparisons. Data are represented as non-linear regression with mean ± SEM.

In all panels, representative of at least three independent experiments is shown unless stated otherwise. The numbers below the immunoblot lines represent the relative signal quantification (see also Supplemental Table 5).
