# Supplementary material for: SMYD3 Impedes Small Cell Lung Cancer Sensitivity to Alkylation Damage through RNF113A Methylation–Phosphorylation Cross-talk
Source: Cancer Discov. 2022 Jul 12;12(9):2158–79. doi: 10.1158/2159-8290.CD-21-0205 (PMC9437563; doi:10.1158/2159-8290.CD-21-0205)
Supplement: Supplementary Data [file cd-21-0205_supplementary_data_4_suppsd4.pdf]

Supplementary Figure 4

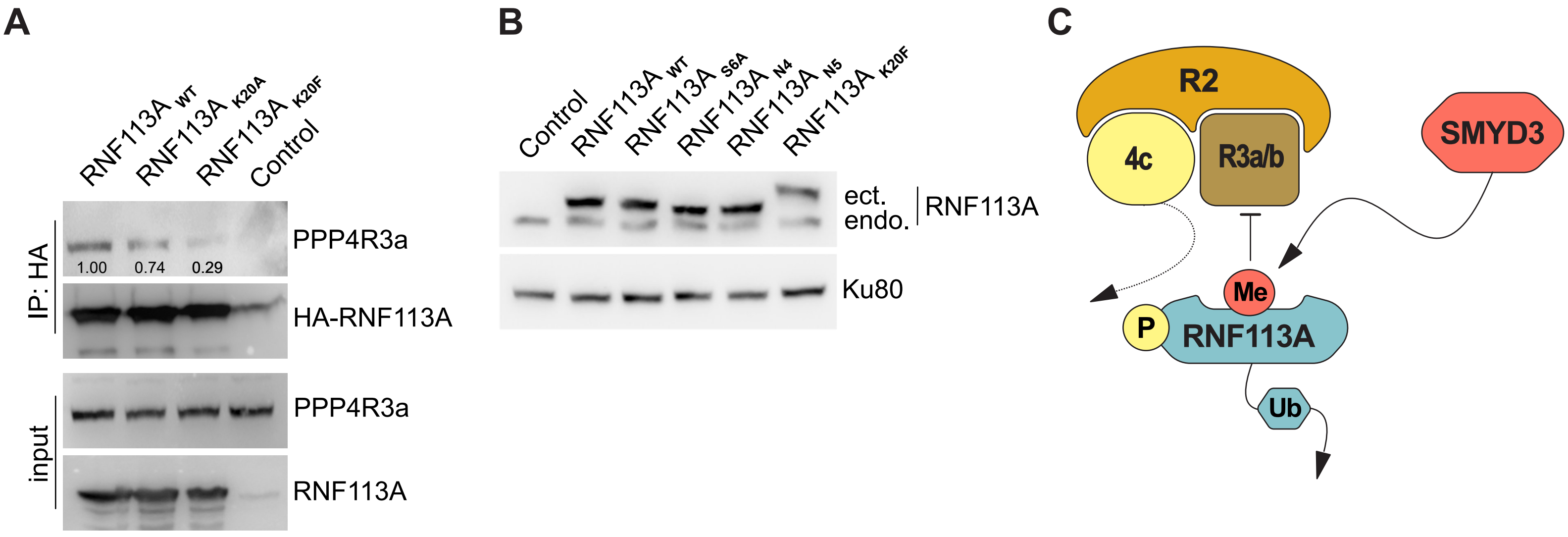

Supplementary Figure S4. RNF113A is a phosphoprotein and its methylation repels the phosphatase PP4

A, Immunoblot analysis was performed with the indicated antibodies after co-immunoprecipitation of endogenous PPP4R3a from 293T cell extracts expressing HA-RNF113A WT, K20A or K20F mutants. **B**, Immunoblot analysis of RNF113A migration using engineered HeLa extracts with stable expression of RNF113A wildtype, S6A, N4, N5 and K20F mutants. Ku80 is shown as a loading control. **C**, Model of RNF113A regulation by the crosstalk of post-translational modifications induced by PP4 and SMYD3.

In all panels, representative of at least three independent experiments is shown unless stated otherwise. The numbers below the immunoblot lines represent the relative signal quantification (see also Supplemental Table 5).
