# Supplementary material for: SMYD3 Impedes Small Cell Lung Cancer Sensitivity to Alkylation Damage through RNF113A Methylation–Phosphorylation Cross-talk
Source: Cancer Discov. 2022 Jul 12;12(9):2158–79. doi: 10.1158/2159-8290.CD-21-0205 (PMC9437563; doi:10.1158/2159-8290.CD-21-0205)
Supplement: Supplementary Data [file cd-21-0205_supplementary_data_5_suppsd5.pdf]

Supplementary Figure 5

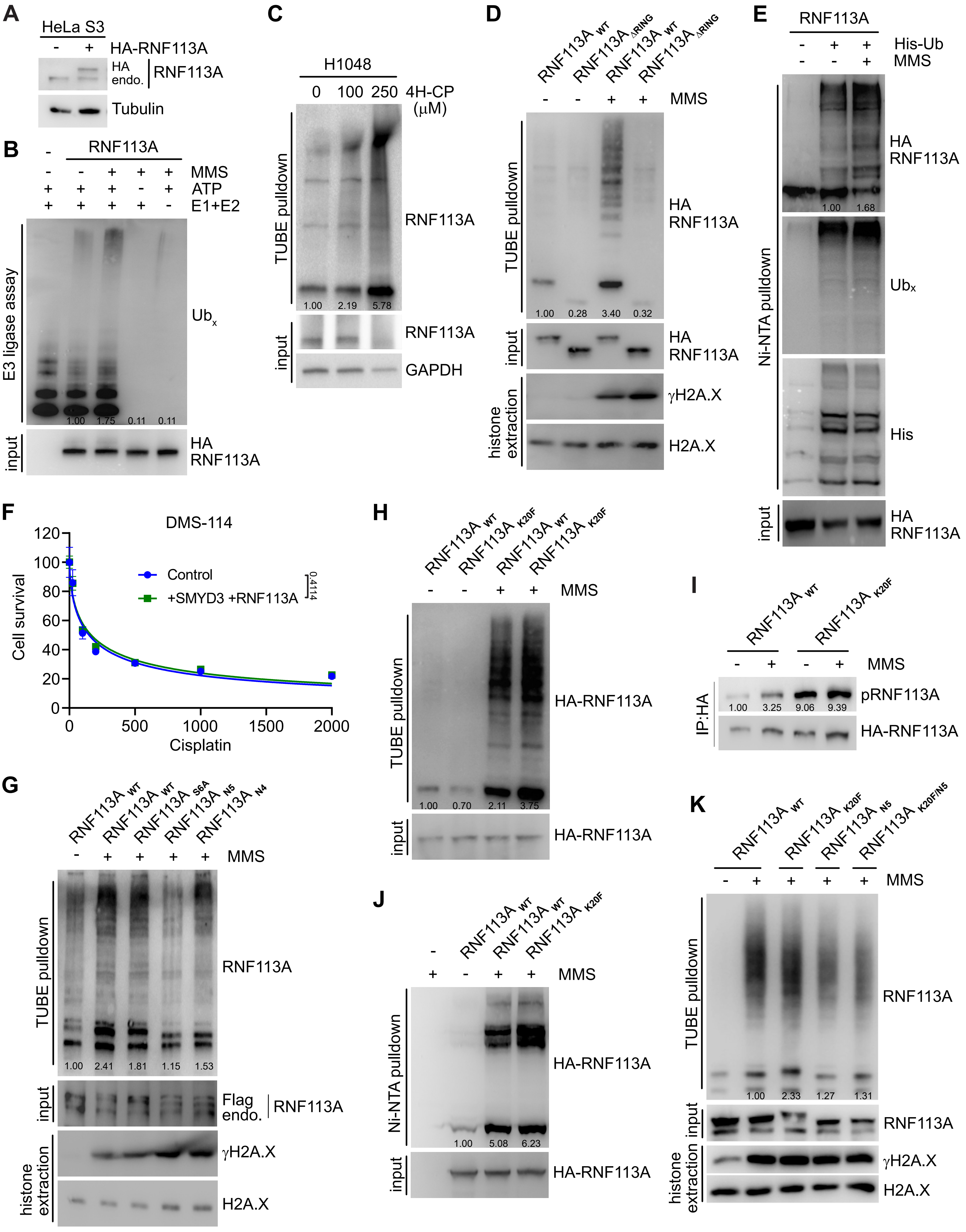

Supplementary Figure S5. Methylation-phosphorylation crosstalk regulation of RNF113A impacts its E3 ligase activity

A, Immunoblot analysis was performed using the indicated antibodies with lysates of HeLa S3 engineered cells. Tubulin is shown as a loading control. **B**, Immunoblot analysis was performed with the indicated antibodies after *in vitro* E3 ubiquitin ligase activity assays using HA-RNF113A purified from HeLa S3 cells, with or without prior alkylating agent (MMS) treatment. ATP and E1/E2 enzymes were added as shown. **C**, Immunodetection of auto-ubiquitinated RNF113A after TUBE (tandem ubiquitin binding element) pulldowns using H1048 SCLC cells extracts following treatment with 4H-CP. **D**, Immunoblot analysis was performed with the indicated antibodies after TUBE pulldowns from HeLa cells stably expressing RNF113A wildtype or catalytically inactive RNF113A ΔRING mutant, with or without MMS-induced alkylation damage. DNA damage marker γH2A.X is shown as a control of damage induction. **E**, Immunoblot analysis was performed with the indicated antibodies demonstrating RNF113A auto-ubiquitination after Ni-NTA pulldown from 293T cells with or without His-Ub ectopic expression and MMS treatment as shown. **F**, Cell survival assays using increasing concentrations of cisplatin in control and engineered DMS-114 SCLC cells with stable expression of SMYD3 and RNF113A. Percentage of living cells under each condition was normalized to untreated cells. *P-value* were calculated by two-way ANOVA with Tukey's testing for multiple comparisons. Data are represented as non-linear regression with mean ± SEM. **G**, Immunoblot analysis was performed with the indicated antibodies after TUBE pulldowns from HeLa cells stably expressing HA-RNF113A wildtype, S6A, N4 or N5 mutants, with or without MMS treatment. **H**, Immunoblot analysis was performed with the indicated antibodies after TUBE pulldowns from HeLa cells stably expressing HA-RNF113A wildtype or K20F mutant, with or without MMS treatment. **I**, Immunoblot analysis was performed with the indicated antibodies using HeLa cells stably expressing either RNF113A wildtype or K20F mutant, with or without MMS treatment. **J**, Immunoblot analysis of auto-ubiquitinated RNF113A after Ni-NTA pulldown from 293T cells with ectopic expression of His-Ub, HA-RNF113A wildtype or K20F mutant and MMS treatment; where indicated. **K**, Immunoblot analysis was performed with the indicated antibodies after TUBE pulldowns from HeLa cells stably expressing HA-RNF113A wildtype, K20F, N5 or K20F/N5 mutants, with or without MMS treatment.

In all panels, representative of at least three independent experiments is shown unless stated otherwise. The numbers below the immunoblot lines represent the relative signal quantification (see also Supplemental Table 5).
