# Supplementary material for: SMYD3 Impedes Small Cell Lung Cancer Sensitivity to Alkylation Damage through RNF113A Methylation–Phosphorylation Cross-talk
Source: Cancer Discov. 2022 Jul 12;12(9):2158–79. doi: 10.1158/2159-8290.CD-21-0205 (PMC9437563; doi:10.1158/2159-8290.CD-21-0205)
Supplement: Supplementary Data [file cd-21-0205_supplementary_data_6_suppsd6.pdf]

Supplementary Figure 6

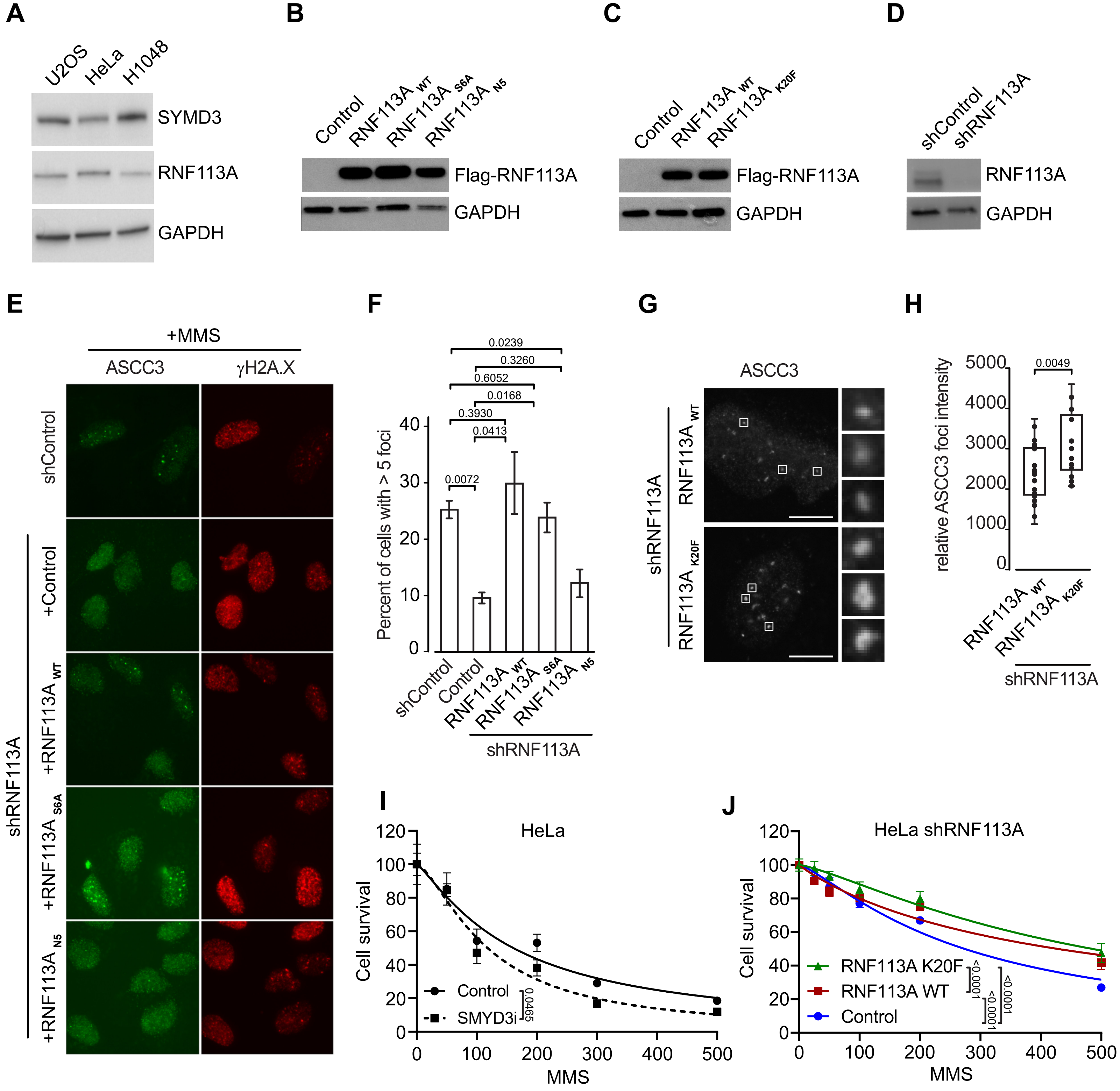

Supplementary Figure S6 (related to Figure 6). RNF113A regulation impacts its function in DNA dealkylation repair

A, Immunoblot analysis with indicated antibodies for comparison of SMYD3 and RNF113A expression levels in HeLa, U20S and H1048 SCLC cells. **B**, Immunoblot analysis was performed using the indicated antibodies with lysates of U2OS cells expressing the indicated vectors. **C**, Immunoblot analysis was performed as in (G) using lysates of U2OS cells expressing the indicated vectors. **D**, Immunoblot analysis of shRNA control (shControl) or shRNA RNF113A knockdown (shRNF113A) in U2OS cells. GAPDH is shown as a loading control. **E**, Representative images of MMS-induced ASCC3 foci in U2OS cells reconstituted with either RNF113A wildtype, S6A or N5 mutants after shRNA knockdown of endogenous RNF113A. Foci were monitored by immunofluorescent staining of ASCC3 (left panels) and the DNA damage marker γH2A.X (right panels). **F**, Quantification of U2OS cells from (E) with five or more MMS-induced ASCC3 foci. At least 100 cells were counted for each experimental condition. *P-value* were calculated by two-tailed unpaired Student's t test, error bars represent mean ± SD. **G**, Representative images of immunofluorescent staining signal intensity of MMS-induced ASCC3 foci in U2OS cells related to Figure 4D. **H**, Quantification of immunofluorescent staining signal intensity of individual ASCC3 foci from RNF113A wildtype (n = 26 foci) and RNF113A K20F mutant (n = 18 foci) expressing U2OS cells as shown in (G). *P-value* were calculated by two-tailed unpaired Student's t test, error bars represent mean ± SEM. **I-J**, Cell survival assays with the indicated concentrations of MMS in HeLa cells with or without SMYD3i (I) or in engineered HeLa cells stably expressing either control vector, RNF113A WT or K20F mutant (J). Percentage of living cells under each condition was normalized to untreated cells. *P-value* were calculated by two-way ANOVA with Tukey's testing for multiple comparisons. Data are represented as non-linear regression of the mean ± SEM.

In all panels, representative of at least three independent experiments is shown unless stated otherwise.
