# Supplementary material for: SMYD3 Impedes Small Cell Lung Cancer Sensitivity to Alkylation Damage through RNF113A Methylation–Phosphorylation Cross-talk
Source: Cancer Discov. 2022 Jul 12;12(9):2158–79. doi: 10.1158/2159-8290.CD-21-0205 (PMC9437563; doi:10.1158/2159-8290.CD-21-0205)
Supplement: Supplementary Data [file cd-21-0205_supplementary_data_7_suppsd7.pdf]

Supplementary Figure 7

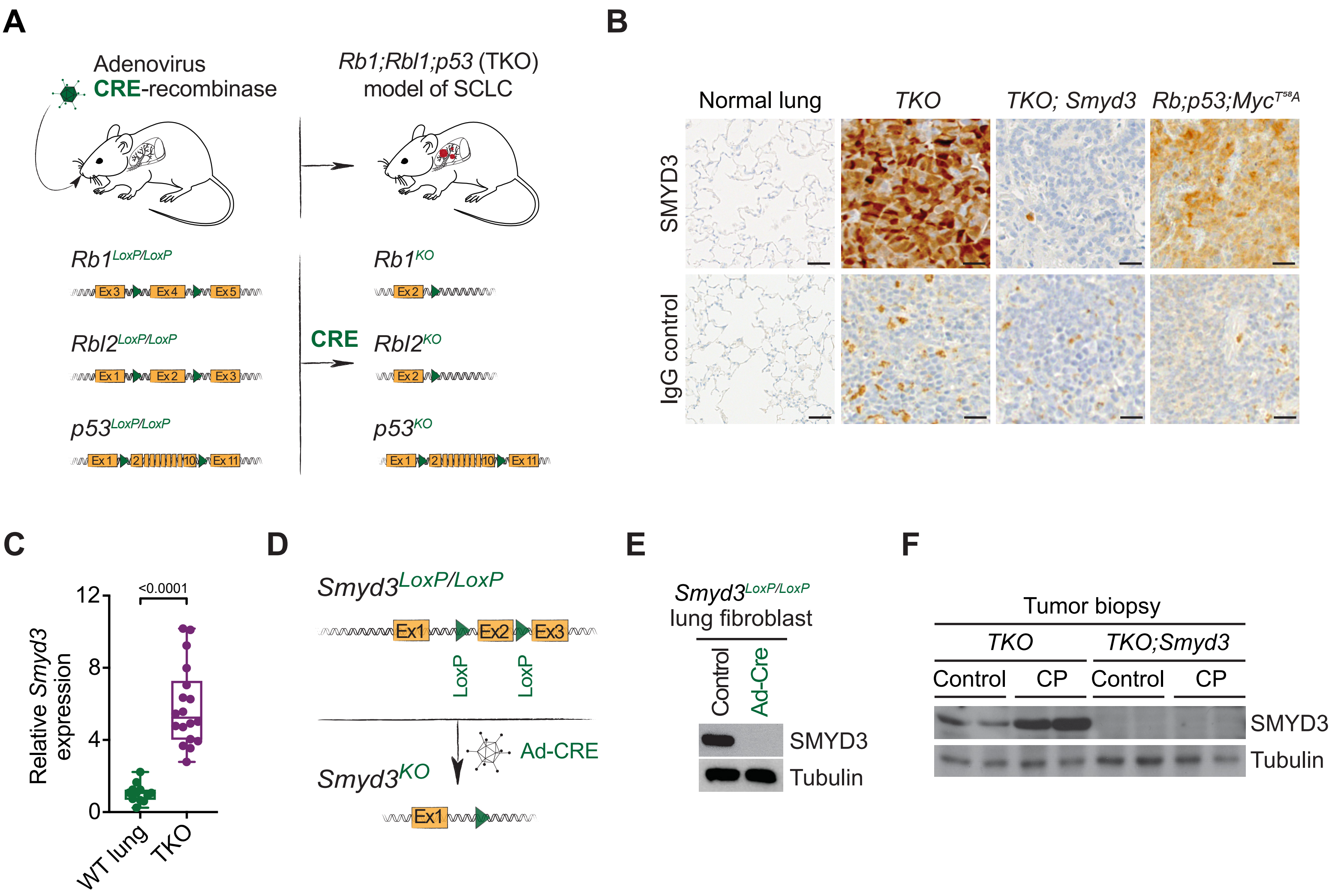

Supplementary Figure S7. SMYD3 inhibition sensitizes SCLC to alkylating agents *in vivo*

A, Schematic of the SCLC mouse model that recapitulates canonical genetic alterations that co-occur in human disease. The triple knockout (TKO) model was generated by breeding mice that carry conditional deletion of *Rb1*$^{LoxP/LoxP}$, *Rbl2*$^{LoxP/LoxP}$ and *Tp53* $^{LoxP/LoxP}$. Tumorigenesis in mice is induced by intratracheal installation of adenovirus expressing Cre-recombinase (Ad-Cre). **B**, Representative IHC staining of normal lung tissue and TKO and RPM (*Rb*$^{LoxP/LoxP}$*;p53*$^{LoxP/LoxP}$*;H11*$^{LSL\text{-}MycT58A}$) SCLC mouse models (representative of n = 12 samples for each group). Of note all analyzed *TKO* and *RPM* samples showed nuclear and cytoplasmic SMYD3 expression with H-score >150. Tumors in *TKO;Smyd3* mutant mice were negative for SMYD3 expression which confirms correct Cre-recombination of mutant allele. Scale bars, 50 µm. **C**, *Smyd3* expression in wildtype lung and *TKO* tumor samples by RTq-PCR, n = 12 samples for each group. *P-values* were calculated by two-tailed unpaired t-test. **D,** Schematic of the *Smyd3* conditional allele. In the presence of Cre recombinase, exon 2 is deleted to disrupt *Smyd3* expression. **E**, Immunoblot analysis with the indicated antibodies of lysates from *Smyd3*$^{LoxP/LoxP}$ lung fibroblasts transduced with Ad-Cre or vehicle (control). Tubulin is shown as a loading control. **F**, Immunoblot analysis with the indicated antibodies of tumor biopsy lysates from *TKO and TKO;Smyd3* mutant mice treated with cyclophosphamide (CP) or vehicle (control). Two independent and representative samples are shown for each condition. Tubulin is shown as a loading control.
