# Supplementary material for: SMYD3 Impedes Small Cell Lung Cancer Sensitivity to Alkylation Damage through RNF113A Methylation–Phosphorylation Cross-talk
Source: Cancer Discov. 2022 Jul 12;12(9):2158–79. doi: 10.1158/2159-8290.CD-21-0205 (PMC9437563; doi:10.1158/2159-8290.CD-21-0205)
Supplement: Supplementary Data [file cd-21-0205_supplementary_data_8_suppsd8.pdf]

Supplementary Figure 8

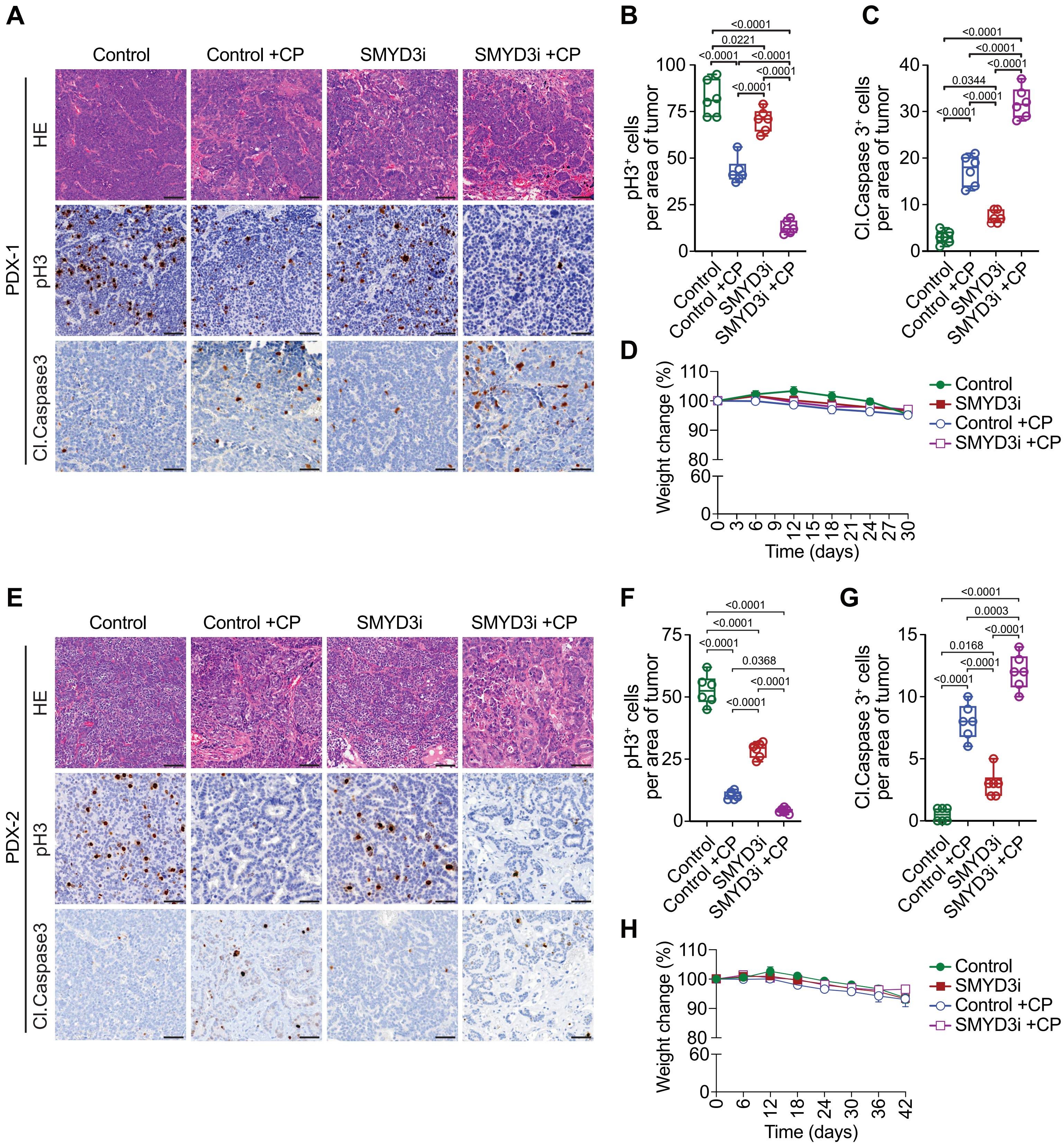

Supplementary Figure S8. SMYD3 inhibition sensitizes SCLC PDX to alkylating agents.

A, Representative H&E and IHC staining for cell proliferation marker phospho-histone 3 (pH3) and apoptosis maker cleaved Caspase 3 (cl. Caspase 3) in biopsies collected from therapy naïve SCLC patient derived xenografts (PDX-1) treated with SMYD3 inhibitor EPZ031686 (SMYD3i) and cyclophosphamide (CP). Representative of n = 6 mice for each experimental group. Scale bars, 50 μm. **B-C**, Quantification of phospho-Histone 3 (pH3) (B) and cleaved Caspase 3 (cl. Caspase 3) positive cells (C) in PDX samples as in (A). Boxes represent 25th to 75th percentile, whiskers: min. to max., center line: median; *P-value* were calculated by two-way ANOVA with Tukey's testing for multiple comparisons. **D**, Weight analysis over time of mouse groups from the PDX-1 study. **E**, Representative H&E and IHC staining for cell proliferation marker phospho-histone 3 (pH3) and apoptosis maker cleaved Caspase 3 (cl. Caspase 3) in biopsies collected from chemotherapy (Carboplatin and Etoposide) relapsed SCLC patient-derived xenografts (PDX-2) treated with SMYD3 inhibitor EPZ031686 (SMYD3i) and cyclophosphamide (CP). Representative of n = 6 mice for each experimental group. Scale bars, 50 μm. **F-G**, Quantification of phospho-Histone 3 (pH3) (F) and cleaved Caspase 3 (cl. Caspase 3) positive cells (G) in PDX samples as in (E). Boxes represent 25th to 75th percentile, whiskers: min. to max., center line: median; *P-value* were calculated by two-way ANOVA with Tukey's testing for multiple comparisons. **H**, Weight analysis over time of mouse groups from the PDX-2 study.
