# Supplementary material for: SMYD3 Impedes Small Cell Lung Cancer Sensitivity to Alkylation Damage through RNF113A Methylation–Phosphorylation Cross-talk
Source: Cancer Discov. 2022 Jul 12;12(9):2158–79. doi: 10.1158/2159-8290.CD-21-0205 (PMC9437563; doi:10.1158/2159-8290.CD-21-0205)
Supplement: Supplementary Data [file cd-21-0205_supplementary_data_suppsm1.docx]

***Lukinović et al. Supplementary Information***

**SMYD3 impedes small cell lung cancer sensitivity to alkylation damage through RNF113A methylation-phosphorylation crosstalk**

**Supplementary Figure Legends**

**Supplementary Figure S1. SMYD3 is a candidate regulator of SCLC susceptibility to alkylating chemotherapy**

**A**, Synthetic lethality screening using a library comprised of 285 characterized inhibitors, testing H209 SCLC cells sensitivity to cisplatin genotoxicity. Data represent relative growth of H209 cells treated with a combination of cisplatin (1 μM) and different inhibitors (1 μM each) compared to cisplatin alone. **B**, Analysis of normal human lung single-cell RNA sequencing data reveals low *SMYD3* expression in pulmonary neuroendocrine cells (PNEC); clusters of cell types are labeled; lung epithelial cell types in red (Human Lung Cell Atlas (41)). **C-H**, H209, H1092 and DMS-114 SCLC cell viability assays using different concentrations of either 4H-CP (C-E) or MMS (F-H) with or without SMYD3i (EPZ031686). Percentage of viable cells under each condition was normalized to untreated cells. *P-value* were calculated by two-way ANOVA with Tukey’s testing for multiple comparisons. Data are represented as non-linear regression with mean ± SEM. **I**, Loewe synergy score calculated by SynergyFinder 2.0, with individual dose-response curves (left) and dose-response matrix (right) for 4H-CP and SMYD3i. **J**, Immunoblot analyses were performed using the indicated antibodies with lysates of H1092 engineered cells used in xenograft assays presented in Figures 1F-H. Actin or Tubulin are shown as a loading control.

**Supplementary Figure S2. Identification of RNF113A as a novel methylated substrate of SMYD3**

**A**. *In vitro* methylation assay were performed using radiolabeled S-adenosylmethionine and recombinant RNF113A and SMYD3, with increasing concentrations of SMYD3 inhibitor (EPZ031686) at the indicated concentrations. Top panel, autoradiogram of methylation assay. Bottom panel, Coomassie stain of proteins in the reaction. **B**, Specific recognition of RNF113A K20me3 peptides by the anti- RNF113A-K20me3 antibody by dot blot analysis using the indicated biotinylated peptides. Streptavidin is shown as the loading control. **C**, Non-radiolabeled *in vitro* methylation assay using recombinant SMYD3 and RNF113A biotinylated peptides with different K20 methylation states as staring material. Methylation events are detected using dot blot and immunodetection by RNF113A K20me3 antibody. Streptavidin is shown as the loading control.

In all panels, representative of at least three independent experiments is shown unless stated otherwise. The numbers below the immunoblot lines represent the relative signal quantification (see also Supplemental Table 5).

**Supplementary Figure S3. Characterization of RNF113A methylation in SCLC cell lines**

**A**, Immunoblot analysis with the indicated antibodies of endogenous RNF113A K20me3 methylation following immunoprecipitation of total RNF113A from HeLa cells expressing doxycycline-inducible shRNA against SMYD3. Tubulin is shown as a loading control. **B**. Immunodetection of RNF113A K20me3 following immunoprecipitation of stably expressed HA-RNF113A in HeLa cells after treatment with different concentrations of SMYD3i. Tubulin is shown as a loading control. **C**, Related to Figure 3D, validation of NAPY markers expressions (NEUROD1; ASCL1; POU2F3; YAP1) in classified SCLC subtypes of human lung cancer. Boxes represent 25th to 75th percentile, whiskers: 10% to 90%, center line: median. *P-value* were calculated by Kruskal-Wallis test. Analysis was performed using FPKM data for each specified gene obtained from NIHMS782739-Suppl_Table10 (34). NAPY SCLC subclassification was based on the original classification by Rudin et al., presented in NIHMS1023395-Supplementary_Table_1 (32).

**D-E**, Pearson correlation analyses of SMYD3 expression and SCLC cell lines resistance to cyclophosphamide (D, ρ = 0.48) and of EZH2 and SLFN11 expressions and SCLC cell lines resistance to platinum-based therapy (E, ρ = 0.16 and ρ = -0.26 respectively). The area under curve (AUC) was calculated by integration under the 16-point concentration-response curves, using the Broad Institute and NCI's Cancer Target Discovery and Development Network: Cancer Therapy Response Portal (CTRP). Pearson correlation coefficient (ρ) was calculated between gene expression and AUC.

**F-G**, Immunoblot analysis was performed with indicated antibodies using lysates of engineered DMS-114 cells (F) which were then used in cell survival assays using different concentrations of MMS (G). Percentage of living cells under each condition was normalized to untreated cells. *P-value* were calculated by two-way ANOVA with Tukey’s testing for multiple comparisons. Data are represented as non-linear regression with mean ± SEM.

In all panels, representative of at least three independent experiments is shown unless stated otherwise. The numbers below the immunoblot lines represent the relative signal quantification (see also Supplemental Table 5).

**Supplementary Figure S4.** **RNF113A is a phosphoprotein and its methylation repels the phosphatase PP4**

**A**, Immunoblot analysis was performed with the indicated antibodies after co-immunoprecipitation of endogenous PPP4R3a from 293T cell extracts expressing HA-RNF113A WT, K20A or K20F mutants. **B**, Immunoblot analysis of RNF113A migration using engineered HeLa extracts with stable expression of RNF113A wildtype, S6A, N4, N5 and K20F mutants. Ku80 is shown as a loading control. **C**, Model of RNF113A regulation by the crosstalk of post-translational modifications induced by PP4 and SMYD3.

In all panels, representative of at least three independent experiments is shown unless stated otherwise. The numbers below the immunoblot lines represent the relative signal quantification (see also Supplemental Table 5).

**Supplementary Figure S5. Methylation-phosphorylation crosstalk regulation of RNF113A impacts its E3 ligase activity**

**A**, Immunoblot analysis was performed using the indicated antibodies with lysates of HeLa S3 engineered cells. Tubulin is shown as a loading control. **B**, Immunoblot analysis was performed with the indicated antibodies after *in vitro* E3 ubiquitin ligase activity assays using HA-RNF113A purified from HeLa S3 cells, with or without prior alkylating agent (MMS) treatment. ATP and E1/E2 enzymes were added as shown. **C**, Immunodetection of auto-ubiquitinated RNF113A after TUBE (tandem ubiquitin binding element) pulldowns using H1048 SCLC cells extracts following treatment with 4H-CP. **D**, Immunoblot analysis was performed with the indicated antibodies after TUBE pulldowns from HeLa cells stably expressing RNF113A wildtype or catalytically inactive RNF113A ΔRING mutant, with or without MMS-induced alkylation damage. DNA damage marker γH2A.X is shown as a control of damage induction. **E**, Immunoblot analysis was performed with the indicated antibodies demonstrating RNF113A auto-ubiquitination after Ni-NTA pulldown from 293T cells with or without His-Ub ectopic expression and MMS treatment as shown. **F**, Cell survival assays using increasing concentrations of cisplatin in control and engineered DMS-114 SCLC cells with stable expression of SMYD3 and RNF113A. Percentage of living cells under each condition was normalized to untreated cells. *P-value* were calculated by two-way ANOVA with Tukey’s testing for multiple comparisons. Data are represented as non-linear regression with mean ± SEM. **G**, Immunoblot analysis was performed with the indicated antibodies after TUBE pulldowns from HeLa cells stably expressing HA-RNF113A wildtype, S6A, N4 or N5 mutants, with or without MMS treatment. **H**, Immunoblot analysis was performed with the indicated antibodies after TUBE pulldowns from HeLa cells stably expressing HA-RNF113A wildtype or K20F mutant, with or without MMS treatment. **I**, Immunoblot analysis was performed with the indicated antibodies using HeLa cells stably expressing either RNF113A wildtype or K20F mutant, with or without MMS treatment. **J**, Immunoblot analysis of auto-ubiquitinated RNF113A after Ni-NTA pulldown from 293T cells with ectopic expression of His-Ub, HA-RNF113A wildtype or K20F mutant and MMS treatment; where indicated. **K**, Immunoblot analysis was performed with the indicated antibodies after TUBE pulldowns from HeLa cells stably expressing HA-RNF113A wildtype, K20F, N5 or K20F/N5 mutants, with or without MMS treatment.

In all panels, representative of at least three independent experiments is shown unless stated otherwise. The numbers below the immunoblot lines represent the relative signal quantification (see also Supplemental Table 5).

**Supplementary Figure S6 (related to Figure 6). RNF113A regulation impacts its function in DNA dealkylation repair**

**A**, Immunoblot analysis with indicated antibodies for comparison of SMYD3 and RNF113A expression levels in HeLa, U20S and H1048 SCLC cells. **B**, Immunoblot analysis was performed using the indicated antibodies with lysates of U2OS cells expressing the indicated vectors. **C**, Immunoblot analysis was performed as in (G) using lysates of U2OS cells expressing the indicated vectors. **D**, Immunoblot analysis of shRNA control (shControl) or shRNA RNF113A knockdown (shRNF113A) in U2OS cells. GAPDH is shown as a loading control. **E**, Representative images of MMS-induced ASCC3 foci in U2OS cells reconstituted with either RNF113A wildtype, S6A or N5 mutants after shRNA knockdown of endogenous RNF113A. Foci were monitored by immunofluorescent staining of ASCC3 (left panels) and the DNA damage marker γH2A.X (right panels). **F**, Quantification of U2OS cells from (E) with five or more MMS-induced ASCC3 foci. At least 100 cells were counted for each experimental condition. *P-value* were calculated by two-tailed unpaired Student’s t test, error bars represent mean ± SD. **G**, Representative images of immunofluorescent staining signal intensity of MMS-induced ASCC3 foci in U2OS cells related to Figure 4D. **H**, Quantification of immunofluorescent staining signal intensity of individual ASCC3 foci from RNF113A wildtype (n = 26 foci) and RNF113A K20F mutant (n = 18 foci) expressing U2OS cells as shown in (G). *P-value* were calculated by two-tailed unpaired Student’s t test, error bars represent mean ± SEM. **I-J**, Cell survival assays with the indicated concentrations of MMS in HeLa cells with or without SMYD3i (I) or in engineered HeLa cells stably expressing either control vector, RNF113A WT or K20F mutant (J). Percentage of living cells under each condition was normalized to untreated cells. *P-value* were calculated by two-way ANOVA with Tukey’s testing for multiple comparisons. Data are represented as non-linear regression of the mean ± SEM.

In all panels, representative of at least three independent experiments is shown unless stated otherwise.

**Supplementary Figure S7. SMYD3 inhibition sensitizes SCLC to alkylating agents *in vivo***

**A**, Schematic of the SCLC mouse model that recapitulates canonical genetic alterations that co-occur in human disease. The triple knockout (TKO) model was generated by breeding mice that carry conditional deletion of *Rb1^LoxP/LoxP^*, *Rbl2^LoxP/LoxP^* and *Tp53 ^LoxP/LoxP^*. Tumorigenesis in mice is induced by intratracheal installation of adenovirus expressing Cre-recombinase (Ad-Cre). **B**, Representative IHC staining of normal lung tissue and TKO and RPM (*Rb^LoxP/LoxP^;p53^LoxP/LoxP^;H11^LSL-MycT58A^*) SCLC mouse models (representative of n = 12 samples for each group). Of note all analyzed *TKO* and *RPM* samples showed nuclear and cytoplasmic SMYD3 expression with H-score >150. Tumors in *TKO;Smyd3* mutant mice were negative for SMYD3 expression which confirms correct Cre-recombination of mutant allele. Scale bars, 50 µm. **C**, *Smyd3* expression in wildtype lung and *TKO* tumor samples by RTq-PCR, n = 12 samples for each group. *P-values* were calculated by two-tailed unpaired t-test. **D,** Schematic of the *Smyd3* conditional allele. In the presence of Cre recombinase, exon 2 is deleted to disrupt *Smyd3* expression. **E**, Immunoblot analysis with the indicated antibodies of lysates from *Smyd3^LoxP/LoxP^* lung fibroblasts transduced with Ad-Cre or vehicle (control). Tubulin is shown as a loading control. **F**, Immunoblot analysis with the indicated antibodies of tumor biopsy lysates from *TKO and TKO;Smyd3* mutant mice treated with cyclophosphamide (CP) or vehicle (control). Two independent and representative samples are shown for each condition. Tubulin is shown as a loading control.

**Supplementary Figure S8.** **SMYD3 inhibition sensitizes SCLC PDX to alkylating agents.**

**A**, Representative H&E and IHC staining for cell proliferation marker phospho-histone 3 (pH3) and apoptosis maker cleaved Caspase 3 (cl. Caspase 3) in biopsies collected from therapy naïve SCLC patient derived xenografts (PDX-1) treated with SMYD3 inhibitor EPZ031686 (SMYD3i) and cyclophosphamide (CP). Representative of n = 6 mice for each experimental group. Scale bars, 50 μm. **B-C**, Quantification of phospho-Histone 3 (pH3) (B) and cleaved Caspase 3 (cl. Caspase 3) positive cells (C) in PDX samples as in (A). Boxes represent 25th to 75th percentile, whiskers: min. to max., center line: median; *P-value* were calculated by two-way ANOVA with Tukey’s testing for multiple comparisons. **D**, Weight analysis over time of mouse groups from the PDX-1 study. **E**, Representative H&E and IHC staining for cell proliferation marker phospho-histone 3 (pH3) and apoptosis maker cleaved Caspase 3 (cl. Caspase 3) in biopsies collected from chemotherapy (Carboplatin and Etoposide) relapsed SCLC patient-derived xenografts (PDX-2) treated with SMYD3 inhibitor EPZ031686 (SMYD3i) and cyclophosphamide (CP). Representative of n = 6 mice for each experimental group. Scale bars, 50 μm. **F-G**, Quantification of phospho-Histone 3 (pH3) (F) and cleaved Caspase 3 (cl. Caspase 3) positive cells (G) in PDX samples as in (E). Boxes represent 25th to 75th percentile, whiskers: min. to max., center line: median; *P-value* were calculated by two-way ANOVA with Tukey’s testing for multiple comparisons. **H**, Weight analysis over time of mouse groups from the PDX-2 study.

**Supplementary Tables**

**Supplementary Table S1.** **List of compounds used in cell growth inhibition screen with 4-hydroperoxy-cyclophosphamide (4H-CP) and cisplatin.** Data represents ratio of 4H-CP or cisplatin to DMSO cell growth ± SD.

| **Compound** | **Supplier** | **Relative growth 4H-CP / DMSO** | **SD** | **Relative growth Cisplatin / DMSO** | **SD** | **Target** |
| --- | --- | --- | --- | --- | --- | --- |
| Niraparib | Selleckchem | 0.322 | 0.004 | 0.451 | 0.015 | PARP |
| EPZ031686 | MedChemExpress | 0.334 | 0.008 | 0.905 | 0.003 | SMYD3 |
| CM10 | Selleckchem | 0.344 | 0.004 | 0.788 | 0.002 | Aldehyde dehydrogenase 1A family |
| NU1025 | Selleckchem | 0.357 | 0.005 | 0.525 | 0.003 | PARP |
| EPZ030456 | SGC | 0.394 | 0.003 | 0.849 | 0.011 | SMYD3 |
| KU-60019 | Selleckchem | 0.416 | 0.006 | 0.903 | 0.011 | ATM |
| 5-Aza-2′-deoxycytidine | Sigma | 0.431 | 0.003 | 0.417 | 0.002 | 5 aza 2 deoxycytidine |
| Buthionine sulfoximine (BSO) | Selleckchem | 0.432 | 0.004 | 0.677 | 0.001 | Glutathione (GSH) synthesis |
| AZD6738 | Selleckchem | 0.474 | 0.004 | 0.494 | 0.004 | ATR |
| Etoposide | Selleckchem | 0.495 | 0.004 | 0.560 | 0.016 | DNA topoisomerase II |
| Pemetrexed Disodium | Selleckchem | 0.523 | 0.003 | 0.647 | 0.006 | Antifolate and antimetabolite |
| AZD7762 | Selleckchem | 0.535 | 0.005 | 0.404 | 0.012 | Chk1/2 |
| BAY-876 | Selleckchem | 0.562 | 0.003 | 0.712 | 0.004 | GLUT1 |
| NU7441 (KU-57788) | Selleckchem | 0.598 | 0.004 | 0.445 | 0.008 | DNA-PK inhibitor |
| J4 | Selleckchem | 0.609 | 0.007 | 0.568 | 0.016 | JMJD3 and UTX |
| Topotecan | Selleckchem | 0.656 | 0.003 | 0.562 | 0.013 | Topoisomerase I |
| GSK690693 | Selleckchem | 0.720 | 0.005 | 0.805 | 0.013 | Akt1, Akt2 and Akt3 |
| Decitabine | Selleckchem | 0.728 | 0.007 | 0.660 | 0.016 | DNA methyltransferases |
| A-366 | Selleckchem | 0.754 | 0.007 | 0.711 | 0.006 | G9a/GLP |
| Dinaciclib (SCH727965) | Selleckchem | 0.756 | 0.012 | 0.648 | 0.010 | CDK2, CDK5, CDK1 and CDK9 |
| CFI-400945 | Selleckchem | 0.784 | 0.001 | 0.667 | 0.001 | Plk4 |
| RGFP966 | Selleckchem | 0.784 | 0.010 | 0.749 | 0.004 | HDAC3i |
| SGC2085 | Selleckchem | 0.789 | 0.001 | 0.510 | 0.010 | CARM1 |
| JQ1 | Selleckchem | 0.794 | 0.007 | 0.678 | 0.006 | BETi bromodomain |
| EPZ-6438 | Selleckchem | 0.806 | 0.009 | 0.409 | 0.004 | EZH2 |
| LDC4297 (LDC044297) | Selleckchem | 0.815 | 0.004 | 0.870 | 0.001 | CDK7 |
| Trichostatin A | Sigma-Aldrich | 0.829 | 0.011 | 1.260 | 0.016 | HDACs |
| Paclitaxel | Selleckchem | 0.834 | 0.001 | 0.671 | 0.014 | Microtubule polymer stabilizer |
| UNC0638 | Selleckchem | 0.844 | 0.004 | 0.704 | 0.010 | G9a/GLP |
| OTSSP167 | Selleckchem | 0.854 | 0.008 | 0.782 | 0.013 | MELK |
| EPZ015666 (GSK3235025) | Selleckchem | 0.869 | 0.005 | 0.832 | 0.003 | PRMT5 |
| Ganetespib (STA-9090) | Selleckchem | 0.878 | 0.010 | 0.890 | 0.002 | Hsp90 |
| Fingolimod (FTY720) | Selleckchem | 0.882 | 0.007 | 0.982 | 0.004 | S1P receptor agonist |
| Doxorubicin | Selleckchem | 0.894 | 0.004 | 0.867 | 0.007 | DNA topoisomerase II |
| PF-3758309 | Selleckchem | 0.905 | 0.003 | 0.881 | 0.015 | PAK |
| Omipalisib (GSK2126458) | Selleckchem | 0.906 | 0.012 | 0.890 | 0.001 | p110α/β/δ/γ, mTORC1/2 |
| Capecitabine | Selleckchem | 0.907 | 0.011 | 0.778 | 0.008 | Prodrug of 5-fluorouracil |
| Afatinib (BIBW2992) | Selleckchem | 0.909 | 0.014 | 0.983 | 0.006 | EGFR/HER2 |
| SIS3 HCl | Selleckchem | 0.910 | 0.016 | 0.987 | 0.006 | Smad3 |
| PAC-1 | Selleckchem | 0.915 | 0.001 | 0.562 | 0.013 | Procaspase-3 activator |
| Ponatinib (AP24534) | Selleckchem | 0.917 | 0.006 | 0.863 | 0.013 | Multi-target kinase inhibitor |
| NMS-873 | Selleckchem | 0.920 | 0.016 | 0.994 | 0.006 | p97 |
| Autophinib | Selleckchem | 0.921 | 0.014 | 0.923 | 0.001 | Autophagy |
| Trametinib (GSK1120212) | Selleckchem | 0.927 | 0.009 | 0.888 | 0.005 | MEK1/2 |
| BI-847325 | Selleckchem | 0.935 | 0.001 | 0.865 | 0.005 | Dual MEK1/2 and Aurora |
| Dabrafenib (GSK2118436) | Selleckchem | 0.943 | 0.013 | 0.886 | 0.016 | BRAF-V600 |
| OTS964 | Selleckchem | 0.951 | 0.007 | 0.975 | 0.003 | TOPK |
| Cisplatin | Selleckchem | 0.957 | 0.012 | N/A | N/A | DNA adduct |
| NSC 319726 | Selleckchem | 0.959 | 0.006 | 1.004 | 0.011 | p53(R175) mutant reactivator |
| YU238259 | Selleckchem | 0.962 | 0.008 | 0.869 | 0.003 | Homology-dependent DNA repair |
| Streptozotocin (STZ) | Selleckchem | 0.963 | 0.008 | 1.034 | 0.005 | Streptozotocin |
| NCB-0846 | Selleckchem | 0.968 | 0.007 | 0.992 | 0.004 | TNIK |
| SB202190 (FHPI) | Selleckchem | 0.973 | 0.011 | 1.007 | 0.007 | p38α/β |
| Tanzisertib(CC-930) | Selleckchem | 0.973 | 0.014 | 0.863 | 0.004 | JNK |
| Telmisartan | Selleckchem | 0.974 | 0.001 | 1.041 | 0.014 | Angiotensin II receptor antagonist |
| Temozolomide | Selleckchem | 0.974 | 0.006 | 1.072 | 0.013 | Alkylates and cross-links DNA |
| Vismodegib (GDC-0449) | Selleckchem | 0.975 | 0.007 | 1.291 | 0.015 | Hedgehog |
| DDR1 Inhibitor | Sigma-Aldrich | 0.976 | 0.013 | 0.566 | 0.017 | DDR1/2 |
| BAY598 | Bayer | 0.977 | 0.011 | 0.971 | 0.016 | SMYD2 |
| LY2584702 | Selleckchem | 0.980 | 0.007 | 0.989 | 0.004 | p70S6K |
| Lifirafenib (BGB-283) | Selleckchem | 0.981 | 0.009 | 0.921 | 0.004 | RAF family kinases and EGFR |
| Oltipraz | Selleckchem | 0.981 | 0.011 | 0.980 | 0.011 | Nrf2 activator |
| GW0742 | Selleckchem | 0.981 | 0.021 | 0.942 | 0.003 | PPARβ/δ agonist |
| Vorasidenib (AG-881) | Selleckchem | 0.981 | 0.010 | 0.965 | 0.008 | IDH1 and IDH2 |
| Chloroquine Phosphate | SCBT | 0.982 | 0.011 | 0.870 | 0.009 | Autophagy |
| Ruxolitinib (INCB018424) | Selleckchem | 0.983 | 0.002 | 0.930 | 0.016 | JAK1/2 |
| AS2444697 | Sigma-Aldrich | 0.983 | 0.002 | 0.985 | 0.003 | IRAK4 |
| HJC0152 | Selleckchem | 0.983 | 0.008 | 0.991 | 0.011 | Stat3 |
| PX-12 | Selleckchem | 0.983 | 0.008 | 1.039 | 0.011 | Thioredoxin-1 (Trx-1) |
| Tianeptine sodium | Selleckchem | 0.983 | 0.002 | 1.061 | 0.017 | Serotonin reuptake enhancer |
| APS-2-79 | Selleckchem | 0.983 | 0.010 | 0.896 | 0.004 | RAF |
| NSC87877 | Selleckchem | 0.983 | 0.008 | 0.917 | 0.006 | SHP-1 and SHP-2 |
| AZD7545 | Selleckchem | 0.983 | 0.010 | 0.970 | 0.005 | PDHK |
| A-196 | Selleckchem | 0.983 | 0.010 | 1.049 | 0.004 | SUV420H1 and SUV420H2 |
| CA-4948 | Selleckchem | 0.984 | 0.008 | 0.885 | 0.016 | IRAK4 |
| TG003 | Selleckchem | 0.984 | 0.011 | 1.011 | 0.006 | Cdc2-like kinase (Clk) |
| CHIR-99021 | Selleckchem | 0.985 | 0.012 | 0.790 | 0.012 | GSK-3α and GSK-3β |
| GSK2801 | Selleckchem | 0.985 | 0.011 | 0.888 | 0.006 | Bromodomains BAZ2A/B |
| Salirasib (FTS) | Selleckchem | 0.985 | 0.012 | 0.992 | 0.003 | Prenylated protein methyltransferase |
| K02288 | Selleckchem | 0.985 | 0.009 | 1.098 | 0.015 | ALK2, ALK1 , ALK3 and ALK6 |
| Enzastaurin (LY317615) | Selleckchem | 0.985 | 0.006 | 0.940 | 0.011 | PKCβ |
| PFI-2 HCl | Selleckchem | 0.985 | 0.004 | 0.965 | 0.013 | SETD7 |
| BIBR 1532 | Selleckchem | 0.985 | 0.011 | 0.981 | 0.009 | Telomerase |
| Pevonedistat (MLN4924) | Selleckchem | 0.986 | 0.008 | 0.984 | 0.014 | Nedd8 activating enzyme (NAE) |
| WNK463 | Selleckchem | 0.986 | 0.006 | 0.986 | 0.002 | pan-WNK-kinase |
| Reparixin (Repertaxin) | Selleckchem | 0.986 | 0.015 | 0.991 | 0.004 | CXCR1/R2 |
| SRPIN340 | Selleckchem | 0.986 | 0.013 | 0.991 | 0.008 | SRPK |
| Pirfenidone | Selleckchem | 0.986 | 0.011 | 0.848 | 0.013 | TGF-β |
| LB-100 | Selleckchem | 0.986 | 0.006 | 0.906 | 0.013 | PP2A |
| Quizartinib (AC220) | Selleckchem | 0.986 | 0.008 | 0.991 | 0.005 | FLT3 |
| NVP-BHG712 | Selleckchem | 0.987 | 0.008 | 0.965 | 0.009 | EphB4 |
| iCRT3 | Selleckchem | 0.987 | 0.008 | 0.992 | 0.013 | Antagonist of Wnt/β-catenin |
| Idasanutlin (RG-7388) | Selleckchem | 0.987 | 0.011 | 1.002 | 0.015 | p53-MDM2 interaction |
| BIX 02189 | Selleckchem | 0.987 | 0.011 | 1.035 | 0.013 | MEK5 and ERK5 |
| TH287 | Selleckchem | 0.987 | 0.006 | 1.089 | 0.004 | MTH1 (NUDT1) |
| COTI-2 | Selleckchem | 0.987 | 0.014 | 0.903 | 0.016 | Activator of mutant p53 |
| LGK-974 | Selleckchem | 0.987 | 0.010 | 0.930 | 0.016 | PORCN |
| Salubrinal | Selleckchem | 0.987 | 0.007 | 0.970 | 0.004 | eIF2α |
| LDC1267 | Selleckchem | 0.987 | 0.011 | 0.983 | 0.001 | Mer, Tyro3, and Axl |
| Birinapant | Selleckchem | 0.987 | 0.007 | 0.985 | 0.011 | SMAC mimetic antagonist |
| Resiquimod | Selleckchem | 0.987 | 0.007 | 1.001 | 0.015 | TLR 7/8 agonist |
| Navitoclax (ABT-263) | Selleckchem | 0.988 | 0.006 | 0.893 | 0.001 | Bcl-xL, Bcl-2 and Bcl-w |
| BI-9564 | Selleckchem | 0.988 | 0.002 | 0.906 | 0.001 | BRD9 and BRD7 bromodomains |
| UNC0379 | Selleckchem | 0.988 | 0.004 | 1.011 | 0.006 | SETD8 |
| LY2409881 | Selleckchem | 0.988 | 0.007 | 0.975 | 0.016 | IKK2 |
| PYR-41 | Selleckchem | 0.988 | 0.003 | 0.978 | 0.015 | Ubiquitin-activating enzyme E1 |
| LDC000067 | Selleckchem | 0.988 | 0.008 | 0.993 | 0.001 | CDK9 |
| STF-62247 | Selleckchem | 0.988 | 0.013 | 0.999 | 0.004 | Inhibitor of renal cells lacking VHL |
| Lonidamine | Selleckchem | 0.988 | 0.003 | 1.012 | 0.013 | Hexokinase |
| Tasisulam (LY573636) | Selleckchem | 0.989 | 0.001 | 0.763 | 0.007 | Apoptosis inducer |
| Montelukast | Selleckchem | 0.989 | 0.006 | 0.956 | 0.011 | Leukotriene D 4 (LTD4) antagonist |
| CC-292 (AVL-292) | Selleckchem | 0.989 | 0.008 | 1.080 | 0.002 | BTK |
| YM155 | Selleckchem | 0.989 | 0.005 | 1.201 | 0.008 | Survivin promoter |
| Diacerein | Selleckchem | 0.989 | 0.007 | 0.963 | 0.002 | Interleukin-1B (IL-1B) |
| EPZ020411 | Selleckchem | 0.989 | 0.011 | 0.984 | 0.005 | PRMT6 |
| Sonidegib (NVP-LDE225) | Selleckchem | 0.989 | 0.013 | 0.989 | 0.015 | Smoothened (Smo) antagonist |
| AT13148 | Selleckchem | 0.989 | 0.004 | 0.990 | 0.003 | Multi-AGC kinase inhibitor |
| PF 670462 | Sigma-Aldrich | 0.989 | 0.011 | 1.013 | 0.016 | Casein kinase 1ε and 1δ |
| AZD3965 | Selleckchem | 0.989 | 0.001 | 1.284 | 0.005 | Monocarboxylate transporter |
| S63845 | Selleckchem | 0.990 | 0.002 | 0.716 | 0.007 | MCL1 |
| AMD3465 hexahydrobromide | Selleckchem | 0.990 | 0.001 | 1.038 | 0.004 | CXCR4 antagonist |
| KRpep-2d | Selleckchem | 0.990 | 0.011 | 1.080 | 0.006 | K-Ras(G12D) |
| LY3039478 | Selleckchem | 0.990 | 0.002 | 1.104 | 0.005 | Notch cleavage inhibitor |
| Pioglitazone | Selleckchem | 0.990 | 0.007 | 0.912 | 0.013 | PPARγ agonist |
| Ispinesib (SB-715992) | Selleckchem | 0.990 | 0.006 | 0.981 | 0.014 | Kinesin spindle protein (KSP) |
| BI-D1870 | Selleckchem | 0.990 | 0.008 | 0.988 | 0.013 | RSK1/2/3/4 |
| SF1670 | Selleckchem | 0.990 | 0.001 | 1.005 | 0.010 | PTEN |
| Desloratadine | Selleckchem | 0.990 | 0.001 | 1.117 | 0.001 | Histamine H1 receptor antagonist |
| 10058-F4 | Selleckchem | 0.991 | 0.006 | 0.795 | 0.003 | c-Myc |
| Ipatasertib (GDC-0068) | Selleckchem | 0.991 | 0.009 | 0.846 | 0.013 | Akt1, Akt2, and Akt3 |
| Dovitinib | Selleckchem | 0.991 | 0.008 | 0.906 | 0.008 | Multi-targeted RTK inhibitor |
| Momelotinib (CYT387) | Selleckchem | 0.991 | 0.005 | 0.911 | 0.006 | TBK1, JAK1/JAK2 |
| Nintedanib (BIBF 1120) | Selleckchem | 0.991 | 0.005 | 0.983 | 0.014 | VEGFR, FGFR1/2/3,PDGFRα/β |
| Triptolide (PG490) | Selleckchem | 0.991 | 0.009 | 1.013 | 0.006 | NF-κB |
| BI665915 | Boehringer Ingelheim | 0.991 | 0.009 | 1.037 | 0.006 | FLAP |
| WZ811 | Selleckchem | 0.991 | 0.011 | 0.916 | 0.003 | CXCR4 antagonist |
| ML323 | Selleckchem | 0.991 | 0.006 | 0.963 | 0.012 | USP1/UAF1 |
| Isoprenaline HCl | Selleckchem | 0.991 | 0.013 | 0.975 | 0.005 | β-adrenergic receptor agonist |
| GSK583 | Selleckchem | 0.991 | 0.007 | 1.004 | 0.011 | RIP2 |
| XAV-939 | Selleckchem | 0.991 | 0.001 | 1.013 | 0.007 | Tankyrase1/2 |
| Deltarasin | Selleckchem | 0.991 | 0.001 | 1.173 | 0.002 | KRAS-PDEδ interaction |
| Irinotecan | Selleckchem | 0.992 | 0.005 | 0.408 | 0.009 | Topoisomerase I |
| SGC-CBP30 | Selleckchem | 0.992 | 0.006 | 0.863 | 0.013 | CREBBP/EP300 |
| XL413 (BMS-863233) | Selleckchem | 0.992 | 0.001 | 0.937 | 0.006 | CDC7 |
| CPI-613 | Selleckchem | 0.992 | 0.012 | 0.973 | 0.014 | Pyruvate dehydrogenase |
| GNF-6231 | Selleckchem | 0.992 | 0.004 | 0.977 | 0.013 | Porcupine |
| Galunisertib (LY2157299) | Selleckchem | 0.992 | 0.006 | 0.984 | 0.006 | TGFβ receptor I |
| Necrosulfonamide | Selleckchem | 0.992 | 0.002 | 1.013 | 0.006 | MLKL |
| MK-1775 | Selleckchem | 0.992 | 0.006 | 1.311 | 0.015 | Wee1 |
| EHT 1864 | Selleckchem | 0.992 | 0.003 | 0.866 | 0.011 | Rac1, Rac1b, Rac2 and Rac3 |
| PFI-3 | Selleckchem | 0.992 | 0.013 | 0.944 | 0.014 | SMARCA bromodomain inhibitor |
| HA15 | Selleckchem | 0.992 | 0.008 | 0.963 | 0.011 | BiP/GRP78/HSPA5 |
| LTX-315 | Selleckchem | 0.992 | 0.001 | 0.964 | 0.011 | Bax/Bak-oncolytic peptide |
| CB-839 | Selleckchem | 0.992 | 0.003 | 0.970 | 0.007 | Glutaminase |
| AZ191 | Selleckchem | 0.992 | 0.001 | 0.975 | 0.004 | DYRK1B |
| CCG-203971 | Sigma-Aldrich | 0.992 | 0.013 | 0.978 | 0.001 | Rho/MKL1/SRF |
| Tofacitinib (CP-690550) | Selleckchem | 0.992 | 0.004 | 1.003 | 0.006 | JAK3 |
| GSK2334470 | Selleckchem | 0.992 | 0.004 | 1.014 | 0.008 | PDK1 |
| BI639667 | Boehringer Ingelheim | 0.992 | 0.007 | 1.085 | 0.003 | CCR1 antagonist |
| Pazopanib | Selleckchem | 0.993 | 0.006 | 0.878 | 0.006 | Multi-target kinase inhibitor |
| Regorafenib | Selleckchem | 0.993 | 0.005 | 0.887 | 0.006 | KIT, PDGFRβ, RAF, RET, VEGFR |
| GSK2830371 | Selleckchem | 0.993 | 0.005 | 0.954 | 0.005 | Wip1 phosphatase |
| Super-TDU | Selleckchem | 0.993 | 0.006 | 0.966 | 0.008 | YAP-TEAD inhibitory peptide |
| 4EGI-1 | Selleckchem | 0.993 | 0.005 | 0.970 | 0.002 | eIF4E/eIF4G interaction |
| ROC-325 | Selleckchem | 0.993 | 0.006 | 1.010 | 0.006 | Lysosomal-mediated autophagy |
| Tiplaxtinin (PAI-039) | Selleckchem | 0.993 | 0.001 | 0.959 | 0.004 | PAI-1 |
| Metformin | Sigma-Aldrich | 0.993 | 0.007 | 0.979 | 0.017 | AMPK activator |
| OICR-9429 | Selleckchem | 0.993 | 0.003 | 0.986 | 0.011 | Antagonist of WDR5-MLL-Histone 3 |
| PFK15 | Selleckchem | 0.993 | 0.006 | 0.988 | 0.008 | 6-phosphofructo-2-kinase (PFKFB3) |
| PRI-724 | Selleckchem | 0.993 | 0.003 | 0.989 | 0.012 | Wnt signaling |
| SGC707 | Selleckchem | 0.993 | 0.008 | 1.004 | 0.015 | PRMT3 |
| Liproxstatin-1 | Selleckchem | 0.993 | 0.003 | 1.203 | 0.016 | Ferroptosis inhibitor |
| Pinometostat (EPZ5676) | Selleckchem | 0.994 | 0.004 | 0.808 | 0.008 | DOT1L |
| GSK1904529A | Selleckchem | 0.994 | 0.004 | 0.868 | 0.016 | IGF-1R and IR |
| AZD1208 | Selleckchem | 0.994 | 0.004 | 0.890 | 0.011 | Pim1, Pim2, and Pim3 |
| C646 | Selleckchem | 0.994 | 0.006 | 0.984 | 0.011 | p300 histone acetyltransferase |
| GSK2656157 | Selleckchem | 0.994 | 0.008 | 1.089 | 0.017 | PERK |
| EED226 | Selleckchem | 0.994 | 0.003 | 0.605 | 0.013 | PRC2 |
| Triapine | Selleckchem | 0.994 | 0.001 | 0.981 | 0.017 | Ribonucleotide reductase inhibitor |
| PNU-74654 | Selleckchem | 0.995 | 0.005 | 1.003 | 0.012 | β-catenin and Tcf4 interaction |
| Sunitinib | Selleckchem | 0.995 | 0.001 | 1.033 | 0.012 | Multi-targeted RTK inhibitor |
| RXDX-106 (CEP-40783) | Selleckchem | 0.995 | 0.006 | 0.888 | 0.008 | TYRO3, AXL, MER and MET |
| Crenolanib (CP-868596) | Selleckchem | 0.995 | 0.001 | 0.974 | 0.001 | PDGFRα/β |
| Silmitasertib (CX-4945) | Selleckchem | 0.995 | 0.003 | 0.977 | 0.016 | CK2 (casein kinase 2) |
| BLZ945 | Selleckchem | 0.995 | 0.003 | 1.051 | 0.004 | CSF-1R |
| Thalidomide | Selleckchem | 0.996 | 0.008 | 0.922 | 0.003 | E3 ubiquitin ligase |
| CCG 50014 | Selleckchem | 0.996 | 0.008 | 0.977 | 0.008 | RGS4 |
| ON123300 | Selleckchem | 0.997 | 0.001 | 0.901 | 0.004 | Multi-targeted kinase |
| RK-33 | Selleckchem | 0.997 | 0.005 | 0.986 | 0.012 | DDX3 (a RNA helicase) |
| TIC10 Analogue | Selleckchem | 0.997 | 0.001 | 1.017 | 0.006 | TRAIL |
| Verteporfin | Selleckchem | 0.997 | 0.001 | 0.880 | 0.005 | TEAD–YAP association |
| Sotrastaurin | Selleckchem | 0.997 | 0.001 | 0.997 | 0.006 | pan-PKC |
| ML264 | Selleckchem | 0.998 | 0.006 | 0.981 | 0.014 | Kruppel-like factor 5 (KLF5) |
| BI3802 | Boehringer Ingelheim | 0.999 | 0.011 | 0.869 | 0.004 | BCL6 degrader |
| Crizotinib | Selleckchem | 1.000 | 0.007 | 0.938 | 0.012 | ALK, MET, ROS1 |
| Ferrostatin-1 (Fer-1) | Selleckchem | 1.002 | 0.001 | 1.277 | 0.014 | Ferroptosis inhibitor |
| ORY-1001 | Selleckchem | 1.003 | 0.011 | 0.666 | 0.017 | LSD1/KDM1A |
| BI9627 | Boehringer Ingelheim | 1.003 | 0.006 | 1.003 | 0.006 | NHE1 |
| PF-3644022 | Sigma-Aldrich | 1.004 | 0.003 | 0.988 | 0.005 | MAPK-activated protein kinase-2 |
| BI3812 | Boehringer Ingelheim | 1.004 | 0.007 | 0.990 | 0.004 | BCL6 inhibitor |
| Dipyridamole | Selleckchem | 1.006 | 0.005 | 0.992 | 0.014 | Phosphodiesterase |
| BMS-345541 | Selleckchem | 1.006 | 0.013 | 1.010 | 0.006 | IKK-2 and IKK-1 |
| RBC8 | Selleckchem | 1.006 | 0.007 | 0.973 | 0.006 | RalA and RalB |
| Mechlorethamine | Selleckchem | 1.007 | 0.008 | 0.983 | 0.013 | Alkylating agent |
| Aripiprazole | Selleckchem | 1.007 | 0.005 | 1.039 | 0.003 | 5-HT receptor partial agonist |
| Epacadostat | Selleckchem | 1.007 | 0.013 | 1.159 | 0.016 | IDO1 |
| GSK269962A | Selleckchem | 1.009 | 0.004 | 1.086 | 0.002 | ROCK |
| BI99179 | Boehringer Ingelheim | 1.009 | 0.001 | 1.006 | 0.016 | FAS |
| SF2523 | Selleckchem | 1.010 | 0.010 | 0.993 | 0.006 | PI3Kα/γ, DNA-PK, BRD4, mTOR |
| Staurosporine | Selleckchem | 1.011 | 0.004 | 0.981 | 0.011 | PKCα, PKCγ and PKCη |
| Brigatinib (AP26113) | Selleckchem | 1.011 | 0.012 | 1.023 | 0.002 | ALK and ROS1 |
| RSL3 | Selleckchem | 1.012 | 0.001 | 0.873 | 0.012 | Ferroptosis activator |
| Wortmannin | Sigma-Aldrich | 1.013 | 0.006 | 0.977 | 0.006 | PI3K |
| Vinorelbine Tartrate | Selleckchem | 1.015 | 0.004 | 1.381 | 0.001 | Tubulin |
| OTX015 | Selleckchem | 1.019 | 0.011 | 0.890 | 0.016 | BETi bromodomain |
| LOXO-101 | Selleckchem | 1.019 | 0.001 | 0.884 | 0.014 | pan-TRK |
| SN-38 | Selleckchem | 1.021 | 0.001 | 1.048 | 0.011 | DNA topoisomerase I |
| Fenofibrate | Selleckchem | 1.024 | 0.005 | 0.949 | 0.001 | PPAR agonist |
| XMD8-92 | Selleckchem | 1.037 | 0.008 | 0.913 | 0.006 | ERK5 |
| AI-10-49 | Selleckchem | 1.038 | 0.002 | 1.019 | 0.004 | CBFβ-SMMHC/RUNX1 binding inhibitor |
| JWG-071 | Gray Lab | 1.038 | 0.005 | 1.053 | 0.006 | ERK5 |
| SP600125 | Selleckchem | 1.039 | 0.008 | 0.867 | 0.006 | JNK |
| CPI-0610 | Selleckchem | 1.040 | 0.003 | 0.785 | 0.013 | BETi bromodomain |
| Palbociclib (PD-0332991) | Selleckchem | 1.045 | 0.004 | 1.170 | 0.011 | CDK4/6 |
| Linsitinib (OSI-906) | Selleckchem | 1.047 | 0.008 | 0.868 | 0.005 | IGF-1R |
| BI1347 | Boehringer Ingelheim | 1.048 | 0.001 | 1.093 | 0.016 | CDK8 |
| PTC-209 | Selleckchem | 1.048 | 0.008 | 1.101 | 0.004 | BMI1 |
| Everolimus | Selleckchem | 1.048 | 0.007 | 1.248 | 0.009 | mTOR |
| Poziotinib | Selleckchem | 1.051 | 0.004 | 0.920 | 0.006 | HER1/2/4 |
| Celecoxib | Selleckchem | 1.055 | 0.014 | 0.988 | 0.015 | COX2 inhibitor |
| TH588 | Selleckchem | 1.057 | 0.004 | 1.084 | 0.002 | MTH1 (NUDT1) |
| Nutlin-3 | Selleckchem | 1.064 | 0.004 | 0.997 | 0.001 | MDM2 |
| BGJ398 (NVP-BGJ398) | Selleckchem | 1.064 | 0.008 | 1.012 | 0.014 | FGFR1/2/3 |
| Plinabulin (NPI-2358) | Selleckchem | 1.064 | 0.001 | 1.102 | 0.009 | VDA |
| Defactinib (PF-04554878) | Selleckchem | 1.066 | 0.002 | 0.961 | 0.002 | FAK |
| MK-2206 | Selleckchem | 1.066 | 0.004 | 1.012 | 0.007 | Akt1, Akt2 and Akt3 |
| MRT68921 | Selleckchem | 1.066 | 0.001 | 1.105 | 0.013 | ULK1/2 |
| GSK481 | Selleckchem | 1.069 | 0.006 | 1.123 | 0.008 | RIP1 |
| JIB-04 | Selleckchem | 1.072 | 0.011 | 0.633 | 0.012 | pan-Jumonji histone demethylase |
| Selinexor (KPT-330) | Selleckchem | 1.074 | 0.016 | 1.016 | 0.013 | CRM1/Xpo1 |
| Dactolisib (BEZ235) | Selleckchem | 1.085 | 0.007 | 0.896 | 0.008 | PI3K and mTOR |
| SBI-0640756 | Selleckchem | 1.089 | 0.006 | 0.961 | 0.015 | eIF4G1 |
| BY1002494 | Boehringer Ingelheim | 1.103 | 0.004 | 1.047 | 0.005 | SYK |
| GMX1778 (CHS828) | Selleckchem | 1.106 | 0.008 | 1.157 | 0.001 | NAMPT |
| Dorsomorphin | Selleckchem | 1.109 | 0.002 | 1.085 | 0.004 | AMPK |
| Milciclib (PHA-848125) | Selleckchem | 1.114 | 0.009 | 1.219 | 0.011 | CDK2 |
| Tipiracil hydrochloride | Selleckchem | 1.114 | 0.010 | 1.200 | 0.015 | Thymidine phosphorylase |
| Luminespib (AUY-922) | Selleckchem | 1.114 | 0.013 | 1.301 | 0.014 | HSP90α/β |
| Entinostat (MS-275) | Selleckchem | 1.128 | 0.004 | 0.753 | 0.011 | HDAC1 and HDAC3 |
| LY3214996 | Selleckchem | 1.129 | 0.013 | 1.112 | 0.007 | ERK1/2 |
| VLX1570 | Selleckchem | 1.148 | 0.013 | 1.011 | 0.010 | USP14 |
| eFT-508 | Selleckchem | 1.149 | 0.008 | 1.153 | 0.003 | MNK1/2 |
| SHP099 dihydrochloride | Selleckchem | 1.150 | 0.002 | 0.965 | 0.009 | SHP2 |
| Daporinad (FK866) | Selleckchem | 1.150 | 0.002 | 1.147 | 0.015 | NMPRT |
| Hydroxychloroquine | Selleckchem | 1.154 | 0.011 | 1.039 | 0.012 | Autophagy |
| URMC-099 | Selleckchem | 1.158 | 0.001 | 1.003 | 0.011 | MLK |
| SB743921 | Selleckchem | 1.160 | 0.001 | 0.706 | 0.012 | Kinesin spindle protein (KSP) |
| Bortezomib | Selleckchem | 1.163 | 0.012 | 0.767 | 0.001 | 20S proteasome |
| Entrectinib (RXDX-101) | Selleckchem | 1.163 | 0.010 | 0.989 | 0.003 | pan-TrkA/B/C, ROS1 and ALK |
| CBL0137 | Selleckchem | 1.169 | 0.007 | 1.119 | 0.013 | NF-kB |
| MRT67307 | Selleckchem | 1.173 | 0.012 | 1.107 | 0.015 | IKKϵ and TBK1 |
| Barasertib (AZD1152) | Selleckchem | 1.173 | 0.004 | 1.109 | 0.016 | Aurora B |
| Erlotinib | Selleckchem | 1.173 | 0.001 | 1.116 | 0.012 | EGFR |
| Vorinostat | Selleckchem | 1.181 | 0.011 | 0.805 | 0.007 | HDAC |
| Alisertib (MLN8237) | Selleckchem | 1.181 | 0.004 | 1.102 | 0.004 | Aurora A |
| Doramapimod | Selleckchem | 1.181 | 0.006 | 1.267 | 0.015 | MAPK14 |
| PLX7904 | Selleckchem | 1.182 | 0.007 | 1.203 | 0.016 | RAF |
| BAY 11-7082 | Selleckchem | 1.183 | 0.004 | 1.305 | 0.008 | NF-κB |
| Dasatinib | Selleckchem | 1.203 | 0.006 | 0.856 | 0.012 | Abl, Src and c-Kit |
| LY3009120 | Selleckchem | 1.205 | 0.002 | 1.100 | 0.013 | pan-Raf |
| Ulixertinib (BVD-523) | Selleckchem | 1.213 | 0.013 | 1.017 | 0.017 | ERK1/ERK2 |
| Fluorouracil (5-FU) | Selleckchem | 1.222 | 0.011 | 1.363 | 0.016 | Thymidylate synthase |
| SCH772984 | Selleckchem | 1.234 | 0.005 | 1.123 | 0.001 | ERK1/2 |
| AZD5153 | Selleckchem | 1.263 | 0.001 | 0.819 | 0.003 | BETi bromodomain |
| MSC2530818 | Selleckchem | 1.275 | 0.008 | 1.227 | 0.001 | CDK8 |
| XMD16-5 | Selleckchem | 1.280 | 0.021 | 1.104 | 0.004 | TNK2 |
| BQU57 | Selleckchem | 1.285 | 0.020 | 1.167 | 0.016 | GTPase Ral |
| CPI-455 HCl | Selleckchem | 1.295 | 0.013 | 1.237 | 0.006 | KDM5 |
| BI 2536 | Selleckchem | 1.317 | 0.006 | 0.561 | 0.016 | PLK1 |
| Erastin | Selleckchem | 1.322 | 0.005 | 1.197 | 0.009 | Ferroptosis activator |
| Vildagliptin (LAF-237) | Selleckchem | 1.340 | 0.021 | 1.122 | 0.007 | DPP−4 |
| Volasertib (BI 6727) | Selleckchem | 1.361 | 0.021 | 0.863 | 0.011 | PLK1, PLK2 and PLK3 |
| BI2536 | Boehringer Ingelheim | 1.377 | 0.014 | 0.613 | 0.006 | PLK1 |
| XMU-MP-1 | Selleckchem | 1.391 | 0.004 | 1.411 | 0.005 | MST1/2 |
| Cyclophosphamide | Selleckchem | N/A | N/A | 1.478 | 0.005 | Alkylating agent |

**Supplementary Table S2. List of potential SMYD3 substrates identified by biochemical protein array screen.** Proteins in grey are predicted false positives with potential auto-methylation activity.

| **Gene ID** | **Official Symbol** | **Official Full Name** |
| --- | --- | --- |
| 55114 | ARHGAP17 | Rho GTPase activating protein 17 |
| 136991 | ASZ1 | Ankyrin repeat, SAM and basic leucine zipper domain-containing protein 1 |
| 138199 | CARNMT1 | Carnosine N-methyltransferase 1 |
| 23059 | CLUAP1 | Clusterin associated protein 1 |
| 1312 | COMT | Catechol-O-methyltransferase |
| 10987 | COPS5 | COP9 signalosome subunit 5 |
| 151194 | METTL21a | Methyltransferase like 21A |
| 4000 | LMNA | Lamin A/C |
| 51451 | LCMT1 | Leucine carboxyl methyltransferase 1 |
| 10746 | MAP3K2 | Mitogen-activated protein kinase kinase kinase 2 |
| 26155 | NOC2L | NOC2 like nucleolar associated transcriptional repressor |
| 4869 | NPM1 | Nucleophosmin 1 |
| 27445 | PCLO | Piccolo presynaptic cytomatrix protein |
| 5110 | PCMT1 | Protein-L-isoaspartate(D-aspartate) O-methyltransferase |
| 5303 | PIN4 | Peptidylprolyl cis/trans isomerase, NIMA-interacting 4 |
| 3276 | PRMT1 | Protein arginine N-methyltransferase 1 |
| 55170 | PRMT6 | Protein arginine N-methyltransferase 6 |
| 56341 | PRMT8 | Protein arginine N-methyltransferase 8 |
| 83732 | RIOK1 | RIO kinase 1 |
| 7737 | RNF113A | Ring finger protein 113A |
| 56950 | SMYD2 | SET and MYND domain-containing protein 2 |
| 6672 | SP100 | SP100 nuclear antigen |

**Supplementary Table S3: List of identified proteins binding to RNF113A-K20me0 and RNF113A-K20me3 peptides in the peptide-pulldown quantitative proteomics analysis.** Two independent experiments using forward (Heavy: RNF113A-K20me3, Light: RNF113A-K20me0) and reverse (Heavy: RNF113A-K20me0, Light: RNF113A-K20me3) labeling were performed.

*Table provided in a separate .xls file.*

**Supplementary Table S4. List of RNF113A phosphorylated sites collected from PhosphoSitePlus database and in-house identification by 2 independent mass spectrometry analyses of RNF113A purified from HeLa cells.** Sites in grey are potential substrates for the Serine/Threonine PP4 phosphatase.

| **RNF113A phosphorylated sites** | **PhosphoSitePlus**  **HTP records** | **Peptide sequences identified by LC-MS in this study** |
| --- | --- | --- |
| S6 | 14 | EPIQSTGSMAEQLSPGK |
| S43 | 2 |  |
| S45 | 4 |  |
| S46 | 3 | RPACDPEPGESGSSSDEGCTVVRPEK |
| S47 | 2 |  |
| Y80 | 9 |  |
| S84 | 50 | AAYGDLSSEEEEENEPESLGVVYK |
| S85 | 50 |  |
| Y120 | 10 |  |
| T124 | 1 |  |
| Y153 | 52 |  |
| Y159 | 4 |  |
| Y162 | 1 |  |
| T168 | 2 |  |
| S169 | 3 |  |
| S174 | 1 |  |
| S175 | 2 |  |
| T192 | 1 |  |
| Y244 | 1 |  |
| Y249 | 4 |  |
| S253 | 52 | YGVYEDENYEVGSDDEEIPFK |
| S268 | 1 |  |
| T323 | 1 |  |
| S329 | 6 | ATGEGGASDLPEDPDEDAIPIT |

**Supplementary Table S5. Graphical representations of immunoblots quantification from the study.** Calculation was performed using Image J software comparing the integrated density of immunoblot signals using at least three different exposures. Background was subtracted and when relevant, signal was normalized with proper references (total protein level, level of immunoprecipitated protein, control, untreated condition, …).

**Figure 3**

Panel A Panel B

**Figure 4**

****Panel E Panel I

**Figure 5**

****Panel A Panel B

****Panel C Panel D

****Panel E Panel F

****Panel G

**Figure 6**

****Panel F

**Figure 7**

Panel B

**Figure S2**

Panel A

**Figure S3**

Panel A Panel B

**Figure S4**

Panel A

**Figure S5**

Panel B Panel C

Panel D Panel E

Panel G Panel H

Panel I Panel J

Panel K

**Supplementary References**

Bouyssié D, Hesse A-M, Mouton-Barbosa E, Rompais M, Macron C, Carapito C, Gonzalez de Peredo A, Couté Y, Dupierris V, Burel A, *et al* (2020) Proline: an efficient and user-friendly software suite for large-scale proteomics. *Bioinformatics* 36: 3148–3155

Brickner JR, Soll JM, Lombardi PM, Vågbø CB, Mudge MC, Oyeniran C, Rabe R, Jackson J, Sullender ME, Blazosky E, *et al* (2017) A ubiquitin-dependent signalling axis specific for ALKBH-mediated DNA dealkylation repair. *Nature* 551: 389–393

Chu VT, Weber T, Graf R, Sommermann T, Petsch K, Sack U, Volchkov P, Rajewsky K & Kühn R (2016) Efficient generation of Rosa26 knock-in mice using CRISPR/Cas9 in C57BL/6 zygotes. *BMC Biotechnol* 16: 4

Cox J & Mann M (2008) MaxQuant enables high peptide identification rates, individualized p.p.b.-range mass accuracies and proteome-wide protein quantification. *Nat Biotechnol* 26: 1367–1372

Fushiki H, Kanoh-Azuma T, Katoh M, Kawabata K, Jiang J, Tsuchiya N, Satow A, Tamai Y & Hayakawa Y (2009) Quantification of mouse pulmonary cancer models by microcomputed tomography imaging. *Cancer Sci* 100: 1544–1549

Jonkers J, Meuwissen R, van der Gulden H, Peterse H, van der Valk M & Berns A (2001) Synergistic tumor suppressor activity of BRCA2 and p53 in a conditional mouse model for breast cancer. *Nat Genet* 29: 418–425

Levy D, Liu CL, Yang Z, Newman AM, Alizadeh AA, Utz PJ & Gozani O (2011) A proteomic approach for the identification of novel lysine methyltransferase substrates. *Epigenetics Chromatin* 4: 19

Lim JS, Ibaseta A, Fischer MM, Cancilla B, O’Young G, Cristea S, Luca VC, Yang D, Jahchan NS, Hamard C, *et al* (2017) Intratumoural heterogeneity generated by Notch signalling promotes small-cell lung cancer. *Nature* 545: 360–364

Liu S, Hausmann S, Carlson SM, Fuentes ME, Francis JW, Pillai R, Lofgren SM, Hulea L, Tandoc K, Lu J, *et al* (2019) METTL13 Methylation of eEF1A Increases Translational Output to Promote Tumorigenesis. *Cell* 176: 491-504.e21

Mazur PK, Reynoird N, Khatri P, Jansen PWTC, Wilkinson AW, Liu S, Barbash O, Van Aller GS, Huddleston M, Dhanak D, *et al* (2014) SMYD3 links lysine methylation of MAP3K2 to Ras-driven cancer. *Nature* 510: 283–287

Nakada S, Chen GI, Gingras A-C & Durocher D (2008) PP4 is a gamma H2AX phosphatase required for recovery from the DNA damage checkpoint. *EMBO Rep* 9: 1019–1026

Raymond CS & Soriano P (2007) High-efficiency FLP and PhiC31 site-specific recombination in mammalian cells. *PLoS ONE* 2: e162

Schaffer BE, Park K-S, Yiu G, Conklin JF, Lin C, Burkhart DL, Karnezis AN, Sweet-Cordero EA & Sage J (2010) Loss of p130 accelerates tumor development in a mouse model for human small-cell lung carcinoma. *Cancer Res* 70: 3877–3883

Skarnes WC, Rosen B, West AP, Koutsourakis M, Bushell W, Iyer V, Mujica AO, Thomas M, Harrow J, Cox T, *et al* (2011) A conditional knockout resource for the genome-wide study of mouse gene function. *Nature* 474: 337–342

Tyanova S, Temu T, Sinitcyn P, Carlson A, Hein MY, Geiger T, Mann M & Cox J (2016) The Perseus computational platform for comprehensive analysis of (prote)omics data. *Nat Methods* 13: 731–740

Wang X, Finegan KG, Robinson AC, Knowles L, Khosravi-Far R, Hinchliffe KA, Boot-Handford RP & Tournier C (2006) Activation of extracellular signal-regulated protein kinase 5 downregulates FasL upon osmotic stress. *Cell Death Differ* 13: 2099–2108
